# Aberrant outputs of cerebellar nuclei and targeted rescue of social deficits in an autism mouse model

**DOI:** 10.1093/procel/pwae040

**Published:** 2024-07-27

**Authors:** Xin-Yu Cai, Xin-Tai Wang, Jing-Wen Guo, Fang-Xiao Xu, Kuang-Yi Ma, Zhao-Xiang Wang, Yue Zhao, Wei Xie, Martijn Schonewille, Chris De Zeeuw, Wei Chen, Ying Shen

**Affiliations:** Center for Brain Health, The Fourth Affiliated Hospital of School of Medicine, and International School of Medicine, International Institutes of Medicine, Zhejiang University, Yiwu 322000, China; Department of Physiology and Department of Psychiatry, Sir Run Run Shaw Hospital, Zhejiang University School of Medicine, Hangzhou 310058, China; Institute of Life Sciences, College of Life and Environmental Sciences, Hangzhou Normal University, Hangzhou 311121, China; Center for Brain Health, The Fourth Affiliated Hospital of School of Medicine, and International School of Medicine, International Institutes of Medicine, Zhejiang University, Yiwu 322000, China; Department of Physiology and Department of Psychiatry, Sir Run Run Shaw Hospital, Zhejiang University School of Medicine, Hangzhou 310058, China; Department of Physiology and Department of Psychiatry, Sir Run Run Shaw Hospital, Zhejiang University School of Medicine, Hangzhou 310058, China; Department of Physiology and Department of Psychiatry, Sir Run Run Shaw Hospital, Zhejiang University School of Medicine, Hangzhou 310058, China; Zhejiang Lab, Hangzhou 311500, China; Department of Physiology and Department of Psychiatry, Sir Run Run Shaw Hospital, Zhejiang University School of Medicine, Hangzhou 310058, China; The Key Laboratory of Developmental Genes and Human Disease of the Ministry of Education, School of Life Science and Technology, Southeast University, Nanjing 210096, China; Department of Neuroscience, Erasmus University Medical Center, 3000 CA Rotterdam, The Netherlands; Department of Neuroscience, Erasmus University Medical Center, 3000 CA Rotterdam, The Netherlands; The Netherlands Institute for Neuroscience, Royal Dutch Academy of Arts & Science, 1105 BA Amsterdam, The Netherlands; Department of Physiology and Department of Psychiatry, Sir Run Run Shaw Hospital, Zhejiang University School of Medicine, Hangzhou 310058, China; Center for Brain Health, The Fourth Affiliated Hospital of School of Medicine, and International School of Medicine, International Institutes of Medicine, Zhejiang University, Yiwu 322000, China; Department of Physiology and Department of Psychiatry, Sir Run Run Shaw Hospital, Zhejiang University School of Medicine, Hangzhou 310058, China; Key Laboratory of Medical Neurobiology of Zhejiang Province, Zhejiang University School of Medicine, Hangzhou 310058, China

**Keywords:** cerebellum, thalamus, midbrain, large-scale tracing, autism

## Abstract

The cerebellum is heavily connected with other brain regions, sub-serving not only motor but also nonmotor functions. Genetic mutations leading to cerebellar dysfunction are associated with mental diseases, but cerebellar outputs have not been systematically studied in this context. Here, we present three dimensional distributions of 50,168 target neurons of cerebellar nuclei (CN) from wild-type mice and *Nlgn3*^R451C^ mutant mice, a mouse model for autism. Our results derived from 36 target nuclei show that the projections from CN to thalamus, midbrain and brainstem are differentially affected by *Nlgn3*^R451C^ mutation. Importantly, *Nlgn3*^R451C^ mutation altered the innervation power of CN→zona incerta (ZI) pathway, and chemogenetic inhibition of a neuronal subpopulation in the ZI that receives inputs from the CN rescues social defects in *Nlgn3*^R451C^ mice. Our study highlights potential role of cerebellar outputs in the pathogenesis of autism and provides potential new therapeutic strategy for this disease.

## Graphical Abstract

**Graphical Abstract:**
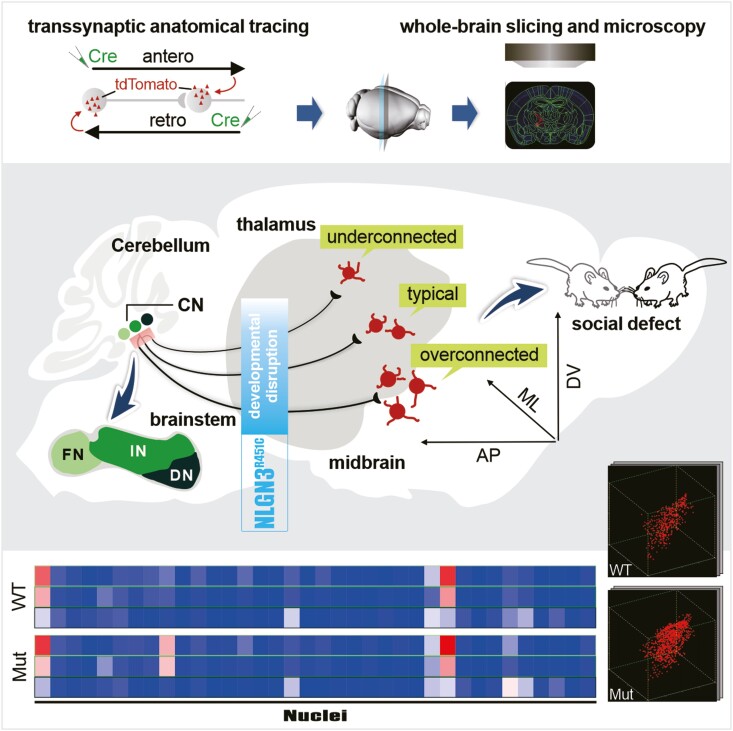

## Introduction

Over the past few decades, the relevance of the “cerebellar connectome” is emerging (Sathyanesan et al., 2019). Indeed, the cerebellum is heavily connected with other brain regions, including the thalamus and the cerebral cortex, sub-serving not only motor but also nonmotor functions. For example, cerebellar nuclei (CN) neurons can actively participate in decision-making (Chabrol et al., 2019; Deverett et al., 2019; Gao et al., 2018) as well as regulating reward, saliency and satiation (Carta et al., 2019; Low et al., 2021). Accordingly, genetic mutations affecting neurons in the cerebellar cortex can result in mental diseases, such as autism (Cupolillo et al., 2016; Peter et al., 2016; Piochon et al., 2014; Reith et al., 2013; Stoodley et al., 2017; Tsai et al., 2012). Given that many mutations that lead to autism affect the development of the cerebellum from early on (Su et al., 2020; Wang et al., 2014), it has been hypothesized that the connectivity of CN neurons with cerebral cortex and subcortical systems may be affected in autism (Peter et al., 2017). This possibility is in line with the finding that the dysfunctional projections from the cerebellum to the prefrontal cortex, one of which passes through the ventromedial thalamus (VM), may contribute to social deficits in tuberous sclerosis (Kelly et al., 2020; Stoodley et al., 2017; Tsai et al., 2012).

We propose that mutations in autism-related genes result in abnormalities in the cerebello-cerebral circuits, leading to autistic-like behaviors. Therefore, it is crucial to understand the impact of these genetic mutations on brain circuits. To elucidate how genetic mutations that affect cerebellar development may lead to autism, we set out to investigate cerebellar outputs in a mouse model with autistic-like behaviors, neuroligin 3-R451C KI (*Nlgn3*^R451C^) mice (Tabuchi et al., 2007). We took advantage of a whole-brain transsynaptic tracing technique to analyze 36 thalamic, midbrain and brainstem nuclei that are innervated by CN outputs in 36 control and *Nlgn3*^R451C^ mice, and created a database containing three-dimensional (3D) coordinates of 50,168 neurons. Our results reveal that *Nlgn3*^R451C^ mutation distinctly changes CN (including fastigial nucleus, FN; interposed nucleus, IN; and dentate nucleus, DN) outputs to the nuclei in the thalamus, midbrain and brainstem. In addition, we show that chemogenetic inhibition of a subpopulation of neurons in the zona incerta (ZI) that receives inputs from the CN is sufficient to rescue social deficits in *Nlgn3*^R451C^ mice, highlighting the possibility that a disbalance in cerebellar outputs contributes to autism. In short, our work suggests that structural connectivity of the cerebellum with other brain regions is affected in autism and partly explain its symptoms.

## Results

### Large-scale transsynaptic tracing reveals differential outputs of FN, IN, and DN onto thalamus, midbrain, and brainstem

**Figure 1.**
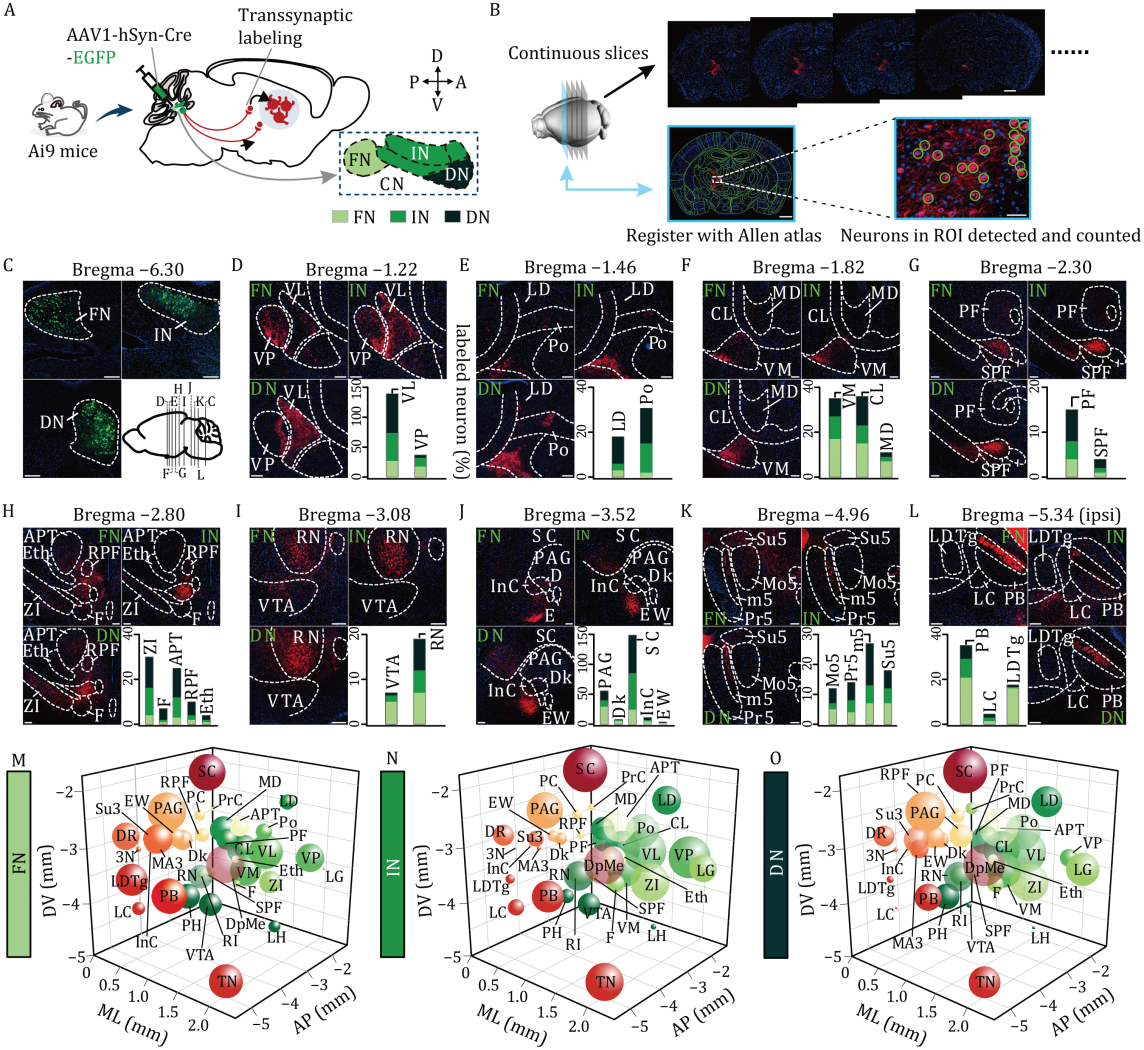
Distinct innervation powers of FN, IN, and DN outputs. (A) Schematic of the injection of anterograde transsynaptic tracer AAV1-Cre-EGFP into FN, IN, or DN in Ai9 mice. (B) Continuous slices (20 μm) were vertically cut in the direction from the rostral to the caudal, and investigators annotated each slice and counted labeled neurons within ROI by referencing to 2D atlas. (C) FN, IN, or DN (bregma −6.30) neurons were labeled by EGFP. Their innervations were examined at annotated bregma levels (D–L) approximately illustrated by the sagittal view of the brain. (D–K) Contralateral neurons innervated by FN, IN, and DN outputs at bregma −1.22 to −4.96. Bar graph shows percentages of neuron counts, relative to injection site, innervated in the annotated nuclei. (L) Ipsilateral neurons innervated by FN, IN, and DN outputs at bregma −5.34. Bottom right: percentages of neuron counts innervated in the PB, LC, and LDTg nuclei. Scale bars: 200 μm. (M–O) Investigated nuclei, as annotated, were depicted according to their 3D coordinates (AP, ML, and DV). The bubble volume indicates the relative number of labeled neurons in individual nucleus. (M) FN injection. (N) IN injection. (O) DN injection. For the statistics, see Table S2.

In order to identify CN-output receiving neurons, we utilized AAV1-hSyn-Cre-EGFP (enhanced green fluorescent protein) virus and Ai9 reporter mice, which express red tdTomato fluorescence in the presence of Cre (Madisen et al., 2010). Infected neurons in the FN, IN, or DN were labeled with EGFP and tdTomato (Fig. 1A). AAV1 can move along axons and infect postsynaptic neurons across monosynaptic connections (Ma et al., 2021; Zingg et al., 2017), turning them red (Fig. 1A). AAV1 was injected into the CN of 30 mouse brains (FN: *n *= 10; IN: *n *= 10; DN: *n *= 10), which were collected 4–5 weeks after the injection. Mouse brains were continuously sectioned at 20-μm interval and registered to the mouse brain atlas (Fig. 1B), and neurons within observed nuclei were identified based on tdTomato-positive cell bodies (Pisano et al., 2021). The specificity of viral injections and the normality of acquired data were determined in two ways (Fig. 1C). First, slices were acquired at five bregma levels for each injection and EGFP fluorescence was examined by registering to the brain atlas (Fig. S1). In all slices (*n *= 150 from 30 mice), only minimal leakage was found in adjacent regions for an injection into a single nucleus (Fig. S2A). Second, the normality of the numbers of infected cells obtained from different experimental mice was confirmed by the Shapiro–Wilk test and quantile (*Q*)-quantile (*Q*) plots (Fig. S2B).

It has been shown that the CN axons project to ipsilateral and contralateral hemispheres (Kebschull et al., 2020; Pisano et al., 2021) and that the projections from parts of the CN to the nuclei in the brain stem show variations (Teune et al., 2000). However, the difference in the FN, IN, and DN-targeted neurons in the thalamus, brain stem, and midbrain has not been quantitatively examined yet. Therefore, we performed continuous slicing and counted neurons in thalamic, midbrain, and brainstem nuclei that were transsynaptically traced from the FN, IN, and DN (Fig. 1D–I). To reduce data variation, the number of target neurons in each nucleus was normalized to the number of starter neurons in the CN. Our results demonstrated that: (i) some thalamic nuclei such as VM, CL, VL, and VPL (for all abbreviations, see Table S1) contained the majority of labeled neurons, consistent with previous work (Pisano et al., 2021); (ii) the numbers of neurons innervated by the FN, IN, and DN outputs were different among nuclei; (iii) innervations by the FN, IN, and DN outputs were remarkably distinct among nuclei; (iv) usually the nuclei contralateral to injection site exhibited more traced neurons, but for some nuclei, such as PB, LC, and LDTg, the ipsilateral side showed more traced neurons (Fig. 1l). For an overview of all innervations, neuronal counts in thalamic, midbrain, and brainstem nuclei were geographically depicted in 3D space (Fig. 1M–O). Taken together, our results reveal remarkable variations of synaptic connections among the FN, IN, and DN and the thalamic, midbrain, and brainstem nuclei.

### Impacts of *Nlgn3*^R451C^ mutation on CN projections to thalamus, midbrain, and brainstem

**Figure 2.**
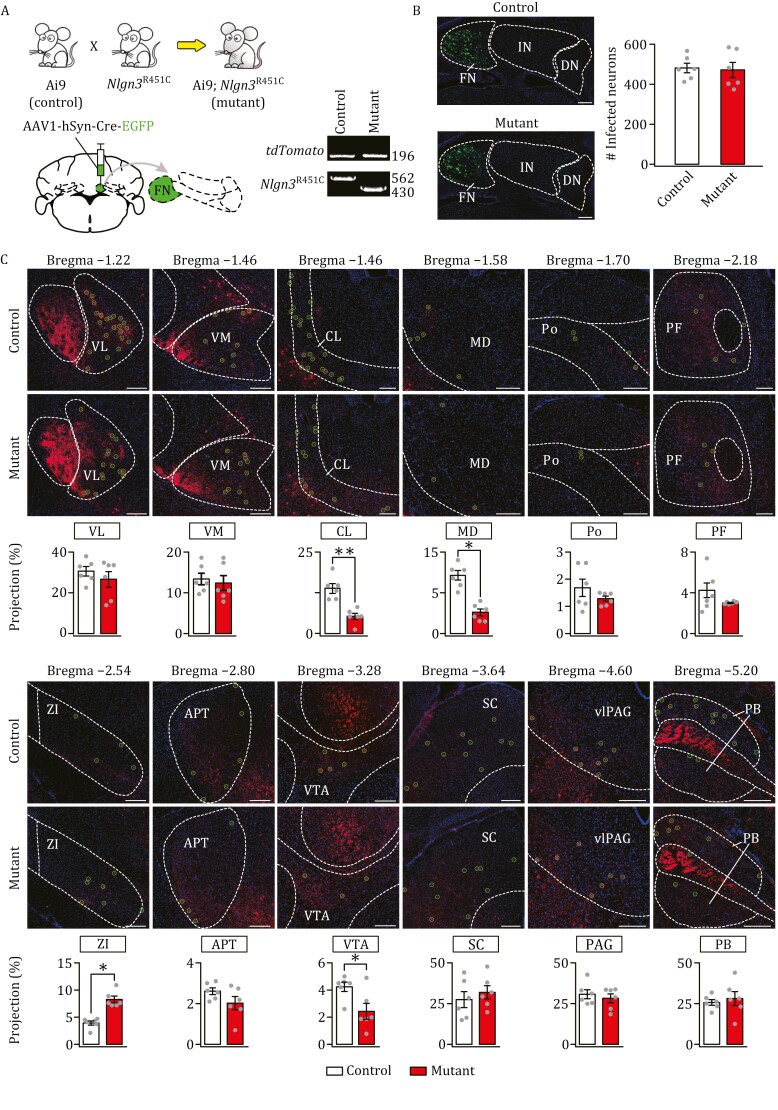
**Differential impact of *Nlgn3*^R451C^ mutation on FN-targeted neurons.** (A) Upper: the production of Ai9;*Nlgn3*^R451C^ (mutant) mice, which were confirmed by PCR detection of *tdTomato* and *Nlgn3*^R451C^. Lower: the injection of AAV1-hSyn-Cre-EGFP in FN. (B) FN neurons were selectively labeled with EGFP in control and mutant mice. Scale bars: 200 μm. Numbers of labeled neurons: control: 475 ± 41 (*n *= 6); mutant: 471 ± 84 (*n* = 6); *F *= 5.09, *t* = 0.095, *P *= 0.93, unpaired *t* test. (C) Images acquired from 12 annotated bregma levels showing FN-recipient zones in VL, VM, CL, MD, Po, PF, ZI, APT, VTA, SC, PAG, and PB. Projection rate was calculated as the ratio of traced neuron count in each nucleus divided by GFP-labeled neuron count in FN. For statistics, see Table S3. *P < 0.05, unpaired *t* test.

The impact of mutations in autism-related genes on cerebellar outputs is largely unclear (Su et al., 2020). We sought to investigate how cerebellar outputs are affected in *Nlgn3*^R451C^ mice, a typical autism model showing defective social interaction (Tabuchi et al., 2007) and cerebellar dysfunctions (Baudouin et al., 2012; Zhang et al., 2015). *Nlgn3*^R451C^ mice were crossed with Ai9 mice to obtain Ai9;*Nlgn3*^R451C^ (mutant) mice, in which neurons expressed native tdTomato upon Cre induction (Fig. 2A). Mutant mice did not show any difference in the intake of either regular or sweet food compared to control mice (Fig. S3A and S3B). Body weight of mutant mice was also normal (Fig. S3C). To examine whether the cross affects autistic phenotype, mutant and control (Ai9) mice were tested in three-chamber interaction task (Zhou et al., 2017). We found that both control and mutant mice exhibited a preference to stranger mouse (S1) (Fig. S4A–C and Table S9). Next, social novelty was examined with the introduction of a second stranger mouse (S2). In this paradigm, control mice exhibited an increased preference for S2, whereas mutant mice showed no preference between S1 and S2 (Fig. S4D–F and Table S9), indicating social impairment in mutant mice. These observations were corroborated by resident-intruder test, showing a significant reduction in the exploration time for familiar mouse of control mice, but not mutant mice. In comparison, both control and mutant mice showed no differences in exploring the novel mouse (Fig. S5A, S5B and Table S10). However, we did not observe difference between control and mutant mice in grooming test (Xu et al., 2023) (Fig. S4G and Table S9).

To determine if *Nlgn3*^R451C^ mutation affects the development of the CN, we performed H&E staining, and found no difference between control and mutant mice in cell numbers of the CN (Fig. S6A and Table S11). There was also no difference between control and mutant mice in vGluT2^+^ and GABA^+^ cells (Fig. S6B, S6C and Table S11), the major types of neurons in the CN (Leto et al., 2006). Therefore, *Nlgn3*^R451C^ mutation does not affect the number of CN neurons. Moreover, Golgi staining and Sholl analysis showed that both intersection number and spine number of Purkinje cells (PCs) and CN neurons were normal in mutant mice (Fig. S7A–D and Table S11), indicating that *Nlgn3*^R451C^ mutation does not affect cell morphology in the cerebellum. In addition, the expression of critical synaptic proteins, including GluA1, GluA2, GluN1, GluN2A, GluN2B, mGlu1, mGlu5, and PSD95, were unchanged in the CN of mutant mice (Fig. S7E and Table S11).

Next, we injected AAV1-hSyn-Cre-EGFP into the FN of control (*n *= 6) and mutant (*n* = 6) mice to drive tdTomato expression (Fig. 2A). Almost no leakage was found in the IN or DN (Fig. S8A) and the numbers of infected cells followed normal distributions according to the Shapiro–Wilk test and *Q*–*Q* plots (Fig. S8B). There was no difference in total infected neurons between control and mutant mice (Fig. 2B). Under these conditions, we counted the neurons that were labeled by anterograde transsynaptic virus in 12 nuclei (VL, VM, CL, MD, Po, PF, ZI, APT, VTA, SC, PAG, and PB). As shown in images obtained from bregma −1.22 to −5.20 (Fig. 2C), the relative number of labeled neurons was significantly different between control and mutant mice, even within a single nucleus. For example, labeled neurons were decreased in the CL, MD, and VTA, but increased in the ZI or unchanged in other nuclei (Fig. 2C).

**Figure 3.**
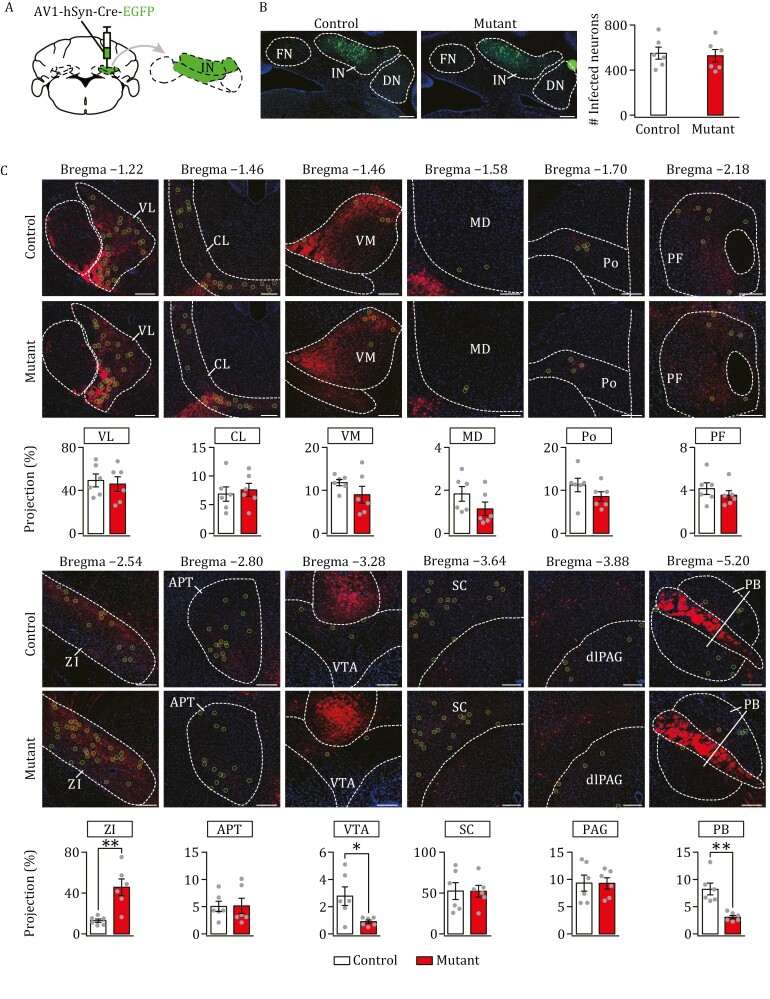
**Differential impact of *Nlgn3*^R451C^ mutation on IN-targeted neurons.** (A) The injection of AAV1-hSyn-Cre-EGFP in IN. (B) IN neurons were selectively labeled with EGFP in control and mutant mice. Scale bars, 200 μm. Numbers of labeled neurons: control: 514 ± 53 (*n *= 6); mutant: 526 ± 123 (*n *= 6); *F *= 7.33, *t* = − 0.20, *P *= 0.85, unpaired *t* test. (C) Images acquired from 12 annotated bregma levels showing IN-recipient zones in various nuclei of control and mutant mice. Projection rate was calculated as the ratio of traced neuron count in each nucleus divided by GFP-labeled neuron count in IN. For statistics, see Table S4. *P < 0.05, unpaired *t* test.

Next, we injected AAV1 into the IN of control (*n *= 6) and mutant (*n *= 6) mice (Fig. 3A). The injection was validated by checking fraction coverage (Fig. S8C), meanwhile the numbers of infected cells followed a normal distribution among investigated animals (Fig. S8D). Again, there was no difference in total infected neurons between control and mutant mice (Fig. 3B). Subsequently, we counted the neurons labeled by the IN injection in thalamic, midbrain, and brainstem nuclei (Fig. 3C). Here too, we found that the number of labeled neurons differed between control and mutant mice in specific nuclei (Fig. 3C). Some, but not all, changes were different from those obtained from the FN-target neurons. For example, labeled neurons were decreased in the VTA and PB, but increased in the ZI (Fig. 3C).

**Figure 4.**
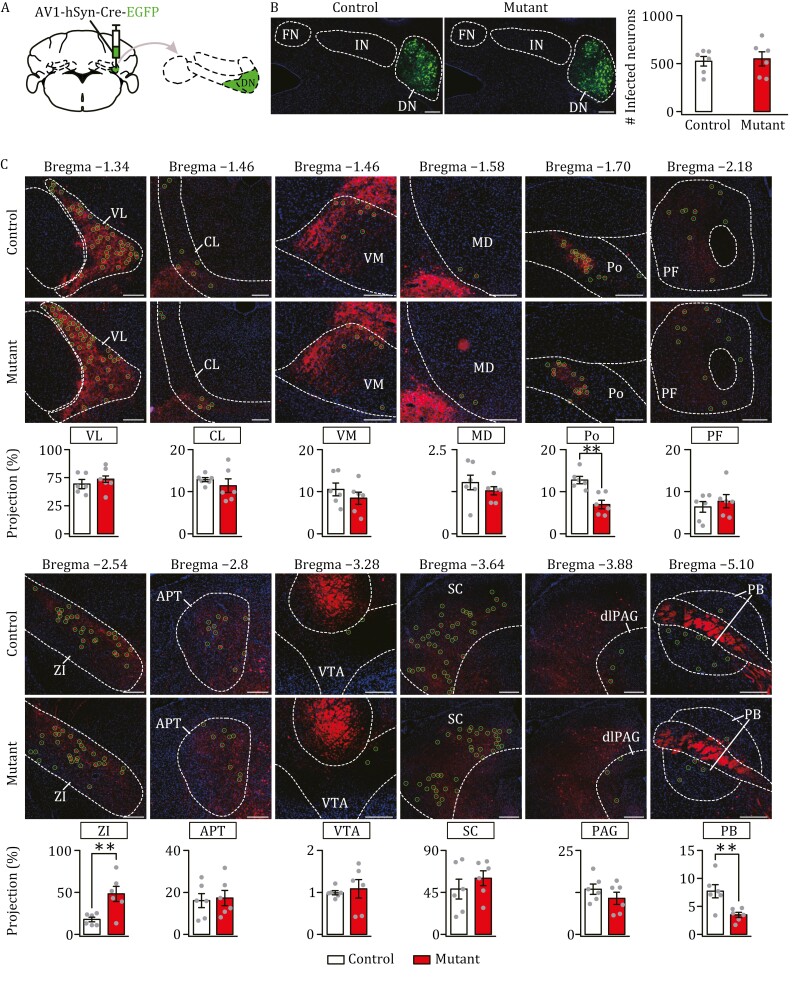
**Differential impact of *Nlgn3*^R451C^ mutation on DN-targeted neurons.** (A) The injection of AAV1-hSyn-Cre-EGFP in DN. (B) DN neurons were selectively labeled with EGFP in control and mutant mice. Scale bars, 200 μm. Numbers of infected neurons: control: 524 ± 112 (*n *= 6); mutant: 549 ± 164 (*n *= 6); *F *= 1.39, *t* = − 0.28, *P *= 0.79, unpaired *t* test. (C) Images acquired from 12 annotated bregma levels showing DN-recipient zones in various nuclei of control and mutant mice. Projection rate was calculated as the ratio of traced neuron count in each nucleus divided by GFP-labeled neuron count in DN. For other statistics, see Table S5. *P < 0.05, unpaired *t* test.

Finally, we injected AAV1-Cre-EGFP into the DN of control (*n *= 6) and mutant (*n *= 6) mice (Fig. 4A). Almost no leakage was found into the FN or IN (Fig. S8E), meanwhile the numbers of infected cells followed a normal distribution among investigated animals (Fig. S8F). There was no difference in total infected neurons between control and mutant mice (Fig. 4B). We counted neurons labeled by the DN injection, and found that the number of labeled neurons was decreased in the PB and Po, increased in the ZI, but unchanged in other nuclei (Fig. 4C). However, *Nlgn3*^R451C^ mutation-induced changes in number of labeled neurons appeared to be more subtle than those neurons receiving projections from the FN or the IN (Fig. 4C).

In summary, we demonstrate that *Nlgn3*^R451C^ mutation changes cerebellar outputs: (i) the synaptic connections were differentially altered in a restricted numbers of thalamic, midbrain and brainstem nuclei (CL, MD, ZI, VTA, Po, and PB), whereas other nuclei were unaltered; and (ii) the mutation-induced change in innervations varied with the FN, IN, and DN, except for a consistent increase in the labeled neurons in the ZI.

### Subregional distributions of FN, IN, and DN projections in recipient nuclei

Next, we investigated the subnuclear distribution of neurons traced following the FN, IN, and DN injections. We recorded 3D coordinates of each transsynaptically labeled neuron from nuclei by registering it with the brain atlas (see Methods for detail). In this way, we created a database containing 50,168 neurons obtained from 18 control and 18 mutant mice (Fig. S9A; For the coordinates of all traced neurons, see online supplementary datasheet), and reconstructed them in 3D space. Based on such analysis, we obtained innervation patterns of the CN outputs onto the thalamic, midbrain, and brainstem nuclei, and were able to uncover how *Nlgn3*^R451C^ mutation affected CN→thalamus, CN→midbrain and CN→brainstem pathways at the subnuclear level. To aid interpretation, we named traced neurons based on the origin and the target of a projection path. For example, the traced neuron of FN→VL pathway was named as VL^FN^ neuron. Although total numbers of VL^FN^, VL^IN^, and VL^DN^ neurons were not affected by *Nlgn3*^R451C^ mutation (Figs. 2–4), ventral VL^FN^ (VL_v_^FN^) neurons at bregma levels from −0.94 to −1.70 and dorsal VL^DN^ (VL_d_^DN^) neurons at bregma levels from −0.94 to −1.34 were increased in mutant mice, suggesting that subpopulations of VL^FN^ and VL^DN^ neurons are differentially affected by *Nlgn3*^R451C^ mutation (Fig. S10 and Table S12; Movies S1–6). We also analyzed subpopulations of ZI^FN^, ZI^IN^, and ZI^DN^ neurons, and found that the number of posterodorsal ZI^IN^ (ZI_pd_^IN^) and ZI^DN^ (ZI_pd_^DN^) neurons at bregma levels from −2.40 to −2.80 was higher, suggesting that ZI neuron subpopulations are also affected by the mutation in a distinct manner (Fig. S11 and Table S12; Movies S7–12).

Using the same method, we analyzed neuronal subpopulations in other nuclei, and found that: (i) MD^FN^ neurons close to the midline (MD_m_^FN^) at bregma levels from−0.94 to −1.94 were increased more than other subpopulations in mutant mice (Fig. S12 and Table S12; Movies S13 and S14); (ii) the reduction in total CL^FN^ neurons in mutant mice was mainly due to the decrease of caudal neurons (CL_c_^FN^) at bregma levels from −1.70 to −1.94 (Fig. S13 and Table S12; Movies S15 and S16); (iii) the decrease of Po^DN^ neurons in mutant mice appeared to be global (Fig. S14; Movies S17 and S18); (iv) VTA^FN^ and VTA^IN^ neurons were much less than traced neurons in other nuclei in control mice. Yet, VTA^FN^ and VTA^IN^ neurons were reduced by the mutation and these changes were also global (Fig. S15; Movies S19–22); and (v) the decrease of PB^IN^ and PB^DN^ neurons was global as well (Fig. S16A–D; Movies S23–26).

Importantly, these results appeared to be consistent among all control and mutant mice we analyzed. To confirm this point, we plotted the numbers of VL^FN^, VL^DN^, CL^FN^, MD^FN^, ZI^FN^, ZI^IN^, and ZI^DN^ neurons from six mutant mice at one Bergma level. It showed that their recipient patterns were similar and statistically there was no difference in homologous blocks between these mice at both mediolateral and dorsoventral directions (Fig. S9B–H). To provide an overview of the subnuclear difference between control and mutant mice, target neurons within 10 nuclei, which showed subregional changes, were plotted together in 3D space (Fig. S17). The subnuclear analysis provides more information about the impact of *Nlgn3*^R451C^ mutation on the spatial aspect of projections.

### Differential effects of *Nlgn3*^R451C^ mutation on FN-, IN-, and DN projections in 36 thalamic, midbrain, and brainstem nuclei

**Figure 5.**
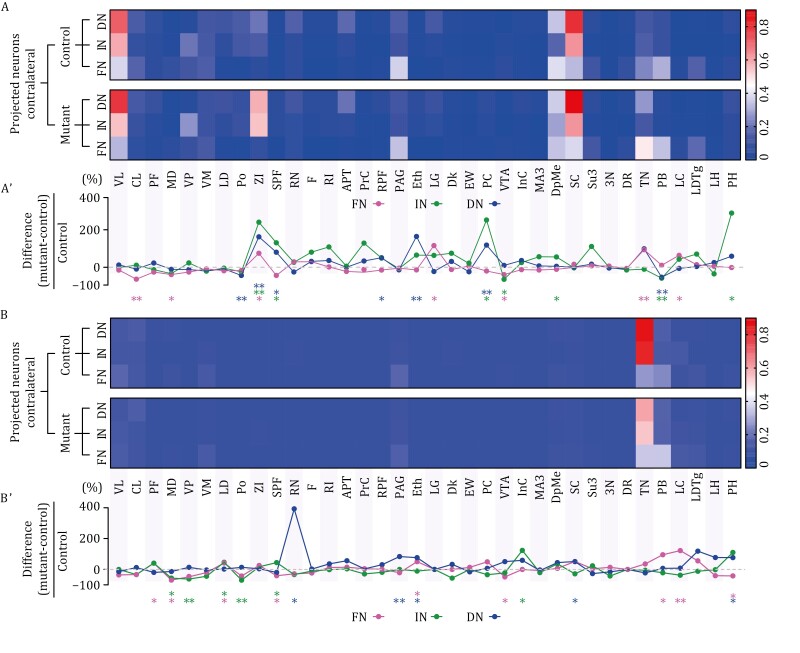
Neuronal counts in 36 nuclei innervated with FN, IN and DN outputs and their differences between control and mutant mice. (A) Heat maps show averaged ratios of traced neuron counts in 36 contralateral nuclei divided by GFP-labeled neuron counts in FN, IN, and DN of control and mutant mice. (Aʹ) The difference for each contralateral nucleus was calculated by subtracting averaged neuron count in mutant mice (*n *= 6) from that in control mice (*n *= 6), and plotted as the percentage change after normalized to neuron count in control mice. (B) Heat maps show the ratios of traced neuron counts in 36 ipsilateral nuclei divided by GFP-labeled neuron counts in FN, IN, and DN of control and mutant mice. (Bʹ) The difference for each ipsilateral nucleus was calculated by subtracting averaged neuron count in mutant mice (*n *= 6) from that in control mice (*n *= 6), and plotted as the percentage change after normalized to neuron count in control mice. For statistics, see Table S6. *P < 0.05. **P < 0.01.

Having demonstrated that *Nlgn3*^R451C^ mutation alters the innervations of the FN, IN, and DN outputs in 12 nuclei, we asked whether other nuclei in the thalamus, midbrain, and brainstem are affected by the mutation as well. Thus, we analyzed the FN-, IN-, and DN-targeted 36 nuclei. Figure 5 shows the numbers of neurons traced by the FN, IN, and DN outputs in nuclei as well as the differences between control and mutant mice, revealing several interesting findings. First, *Nlgn3*^R451C^ mutation exerted specific effects on thalamic, midbrain and brainstem nuclei: it did not affect most nuclei, but greatly altered projections in some, for example contralateral ZI and ipsilateral RN and InC (Fig. 5A and 5B). Second, in several instances, *Nlgn3*^R451C^ mutation differentially affected target neurons located in contralateral *vs* ipsilateral nuclei. To be specific, the mutation: (i) increased target neurons in the contralateral ZI (ZI^FN^, ZI^IN^, and ZI^DN^), CL^FN^, LG^FN^, LC^FN^, TN^FN^, PC^IN,DN^, RPF^DN^, and DpMe^IN^, but hardly affected the same nuclei in the ipsilateral hemisphere; (ii) altered the projections to the ipsilateral PF^FN^, VP^IN^, LD^FN,IN^, InC^IN^, RN^DN^, SC^DN^, and PAG^DN^ neurons with no significant effect on these nuclei in the contralateral hemisphere; or (iii) affected both ipsilateral and contralateral nuclei in a similar manner. For example, both ipsilateral and contralateral MD^FN^ and VTA^FN^ neurons were reduced, whereas both ipsilateral and contralateral SPF^IN^, LC^FN^, and Eth^DN^ neurons were increased in mutant mice (Fig. 5A and 5B). Third, the effects of the mutation on the FN-, IN, and DN-targeted neurons were opposite in some nuclei, such as ipsilateral PH and SPF, but identical in other nuclei, such as contralateral SPF, PB, and ZI (Fig. 5A and 5B).

Next, we asked if the altered neuronal innervations are related to geographical position of the nuclei. To this end, we plotted the 36 nuclei at 7 bregma levels (Fig. S18A), and measured 2D distances between the center point of each nucleus and those of the FN, IN, and DN along both anteroposterior and mediolateral axes. These maps demonstrated that brain regions with increased, decreased or normal FN-projected numbers of FN-targeted neurons were found across all bregma levels, which was also the case for IN- or DN-targeted neurons (Fig. S18A). For an intuitive description, we plotted anteroposterior distances against number changes. No linear function could be defined for the correlation between changed numbers of targeted neurons in a nucleus with 2D distance to the CN (Fig. S19A–C).

How about their relationships along the mediolateral axis? We plotted 36 nuclei at six saggital levels and mapped the number of traced neurons onto the nuclei (Fig. S18B). Similarly, we found that brain regions with increased, decreased, or intact numbers of FN-targeted neurons were present at all mediolateral levels (Fig. S18B). We next plotted the change in labeled neurons per target nucleus against the mediolateral distances of each nucleus to the CN. Here too, we found no correlation of the CN-targeted neurons with the mediolateral distances (Fig. S19D–F). These observations suggest that the changes of CN-targeted neurons are independent of the distance of the nuclei from the CN. It should be noted that the distances were roughly estimated, because 3D straight line distances between the CN and the nuclei cannot be acquired by 2D atlas.

### Identification of VL^IN^ and ZI^IN^ neurons in control and mutant mice

**Figure 6.**
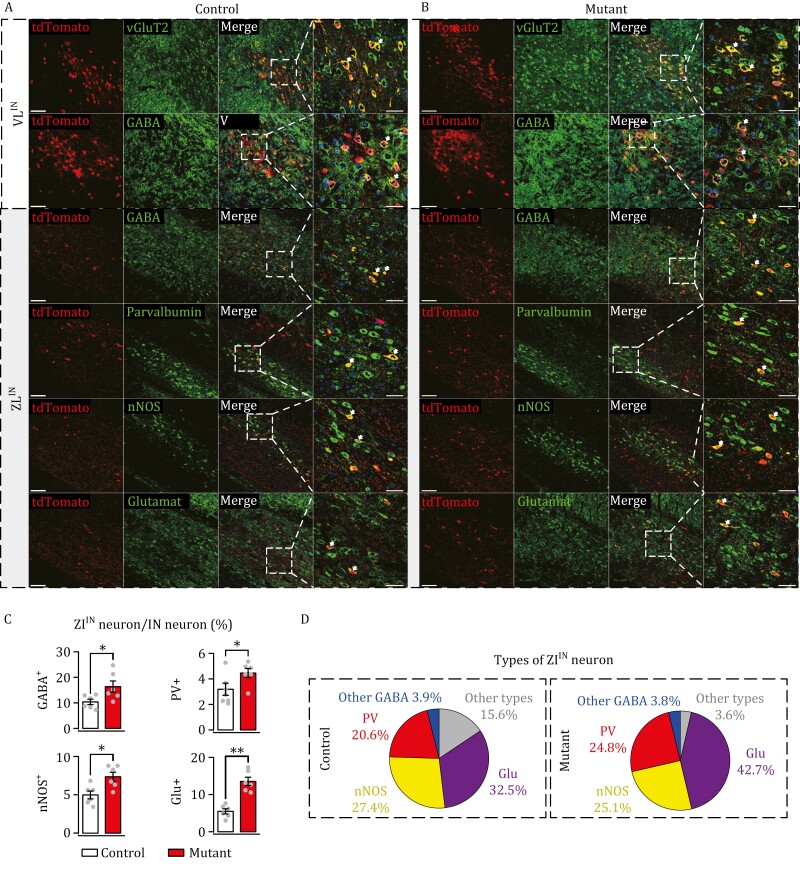
Types of ZI neurons anterogradely traced by IN outputs. (A) tdTomato^+^ VL^IN^ and ZI^IN^ neurons in control mice were immunostained by antibodies against vGluT2, GABA, parvalbumin, nNOS, and glutamate. Most right panel is the higher magnification of the third panel “merge”. Scale bars: 100 μm (3 columns on the left); 50 μm (right column). Arrowheads show dual-color-labeled neurons. *n *= 6 control mice. (B) tdTomato^+^ VL^IN^ and ZI^IN^ neurons in mutant mice were immunostained by antibodies against vGluT2, GABA, parvalbumin, nNOS, and glutamate. Scale bars: 100 μm (3 columns on the left); 50 μm (right column). *n *= 6 mutant mice. (C) Numbers of GABA^+^, glutamate^+^, PV^+^, and nNOS^+^ ZI^IN^ neurons were normalized to the number of GFP-labeled IN neurons. (D) Percentages of glutamate (Glu)^+^, parvalbumin (PV)^+^, nNOS^+^, GABA^+^ (besides PV and nNOS), and other types of neurons among all ZI^IN^ neurons. For statistics, see Table S7. *P < 0.05. **P < 0.01.

To examine the contrasting effects of the mutation between target nuclei, we analyzed the types of target neurons in the VL and ZI. *Nlgn3*^R451C^ mutation did not alter the population of VL^FN^, VL^IN^, and VL^DN^ neurons, whereas contralateral ZI^FN^, ZI^IN^, and ZI^DN^ neurons were increased in mutant mice (Figs. 2C, 3C, and 4C). Immunostaining was performed with antibodies against cell-type markers following anterograde tracing of VL^IN^ and ZI^IN^ neurons, and both of them expressed Cre-driven fluorescent tdTomato following the injection of AAV1-Cre-EGFP in the IN (Fig. 6A and 6B). In VL, immunostaining revealed that tdTomato^+^ neurons expressed vesicle glutamate transporter 2 (vGluT2) or γ-aminobutyric acid (GABA) (Fig. 6A and 6B). Next, immunostaining for GABA, glutamate, parvalbulmin, and neuronal nitric oxide synthase (nNOS) was conducted in the ZI of control and mutant mice following IN injection with AAV1-Cre-EGFP. Similar to VL^IN^ neurons, ZI^IN^ neurons were positive to GABA, glutamate, parvalbulmin, or nNOS (Fig. 6A and 6B), indicating that ZI neurons receiving IN output are either excitatory or inhibitory. To investigate which type of ZI^IN^ neurons is affected by *Nlgn3*^R451C^ mutation, we calculated the ratios of GABA^+^, glutamate^+^, parvalbulmin^+^, and nNOS^+^ ZI^IN^ neurons divided by starter neurons. Our results showed that there was an increase in all types of ZI^IN^ neurons in mutant mice (Fig. 6C). To examine relative proportions of increased ZI^IN^ neurons, we calculated the percentages of GABA^+^, glutamate^+^, parvalbulmin^+^, and nNOS^+^ neurons, and found that glutamate^+^ cells increased most, followed by parvalbulmin^+^ cells, resulting in increased proportions of these types of cells (Fig. 6D). The proportions of nNOS^+^ cells and other GABA^+^ (PV^−^ and nNOS^−^) cells were affected minimally, whereas the proportion of ZI^IN^ neurons not labeled by either glutamate or GABA was reduced significantly (Fig. 6D).

### Retrograde tracing of ZI and VL to CN

In the CN, different types of neurons send ascending fibers to the cerebellar cortex and descending fibers to the nuclei in other brain regions (Fujita et al., 2020). To identify neuronal subtypes in the CN responsible for the projections to ZI and VL, we injected retrograde AAV2 virus containing a vector encoding wheat germ agglutinin Cre fusion protein (EGFP-WGACre) (Yoshihara et al., 1999) into the VL or ZI, and then calculated the number of neurons that were retrogradely and transsynaptically labeled in the FN, IN, and DN (Fig. S20A). ZI neurons were labeled with EGFP, while retrogradely traced neurons in the FN, IN, and DN (termed as FN^ZI^, IN^ZI^, and DN^ZI^) expressed Cre-driven native tdTomato, upon EGFP-WGACre injection in the ZI (Fig. S20B). By calculating the ratios of FN^ZI^, IN^ZI^, and DN^ZI^ neurons to EGFP-labeled ZI neurons, we found that IN^ZI^ and DN^ZI^ neurons were increased significantly, whereas FN^ZI^ neurons were not affected in mutant mice (Fig. S20Bʹ and Table S13). It should be noted that the unaltered number of FN^ZI^ neurons may be attributed to their limited quantity, which could be masked by the inefficiency of retrograde AAV2. Similarly, EGFP-WGACre was injected into the thalamic VL nucleus. When VL neurons were labeled with EGFP, neurons in the FN, IN and DN (termed as FN^VL^, IN^VL^, and DN^VL^) expressed fluorescent tdTomato (Fig. S20C). By calculating the ratios of FN^VL^, IN^VL^, and DN^VL^ neurons to EGFP-labeled VL neurons, we found that none of FN^VL^, IN^VL^, and DN^VL^ neurons was affected in mutant mice (Fig. S20Cʹ and Table S13). These results are in consistent with anterograde tracing showing that CN→ZI pathway, but not the CN→VL pathway, was affected in mutant mice. To specify the types of FN^ZI^, IN^ZI^, and DN^ZI^ neurons, we performed immunostaining using antibodies against vGluT2 or GABA. Our results showed that FN^ZI^, IN^ZI^, and DN^ZI^ neurons could be labeled by vGluT2 or GABA (Fig. S20D, S20E and Table S13). By calculating the ratios of GABA^+^ or vGluT2^+^ FN^ZI^, IN^ZI^, and DN^ZI^ neurons to EGFP-labeled ZI neurons, we found that GABA^+^ or vGluT2^+^ IN^ZI^ and DN^ZI^ neurons were increased significantly in mutant mice (Fig. S20D, S20E and Table S13).

A previous study showed that AAV2 may cause a minor contamination to axonal terminals (Tervo et al., 2016), which might affect our conclusion. To exclude this possibility, we utilized another retrograde tracing technique by injecting RV-EnVA-ΔG-EGFP, rAAV-oRVG, and rAAV-TVA-mCherry, which do not contaminate axonal terminals, into the ZI (Fig. S21A). In this case, infected ZI neurons were also labeled with mCherry and EGFP, whereas FN^ZI^, IN^ZI^, and DN^ZI^ neurons expressed EGFP (Fig. S21B). The calculation showed that the ratios of IN^ZI^ and DN^ZI^ neurons to infected ZI neurons were increased in mutant mice, while the ratio of FN^ZI^ neurons was not changed (Fig. S21C and Table S14).

In addition, we reconstructed spatial distributions of FN^VL^, IN^VL^, DN^VL^, FN^ZI^, IN^ZI^, and DN^ZI^ neurons. We found that the distribution patterns of FN^VL^ (Fig. S22A and S22B), IN^VL^ (Fig. S23A and S23B), and DN^VL^ (Fig. S24A and S24B) neurons were similar between control and mutant mice (Movies S27, S28, S31, S32, S35, and S36). Conversely, the distribution patterns of IN^ZI^ and DN^ZI^ neurons were altered, consistent with the increases in total counts (Fig. S20Bʹ). IN^ZI^ neurons located in posterodorsal IN were increased more than other subpopulations (Fig. S23C and S23D; Movies S33 and S34). DN^ZI^ neurons displayed a pattern different from IN^ZI^ neurons: *Nlgn3*^R451C^ mutation induced more increase in neurons located at the posteroventral DN (Fig. S24C and S24D; Movies S37 and S38). In addition, the number of FN^ZI^ neurons was lower than those of IN^ZI^ and DN^ZI^ neurons (Fig. S22C and S22D; Movie S29). Figure S25 summarized 3D distribution of FN^VL^, IN^VL^, DN^VL^, FN^ZI^, IN^ZI^, and DN^ZI^ neurons in representative control and mutant mice. Interestingly, *Nlgn3*^R451C^ mutation-affected IN^ZI^ and DN^ZI^ neurons were located within a narrow anteroposterior area (bregma −6.36 to −6.50), suggesting that the output of this subpopulation may exert a prominent role in regulating ZI functions.

### Inhibition of ZI^IN^ and ZI^DN^ neurons rescues social defect of *Nlgn3*^R451C^ mice

Next, we wondered about the potential pathological implication of increased ZI^IN^ and ZI^DN^ neurons. Since the prominent feature of *Nlgn3*^R451C^ mice is social novelty (Tabuchi et al., 2007), it was intriguing to ask whether ZI^IN^ and ZI^DN^ neurons are associated with this defect using chemogenetics to inhibit ZI^IN^ and ZI^DN^ neurons in mutant mice.

**Figure 7.**
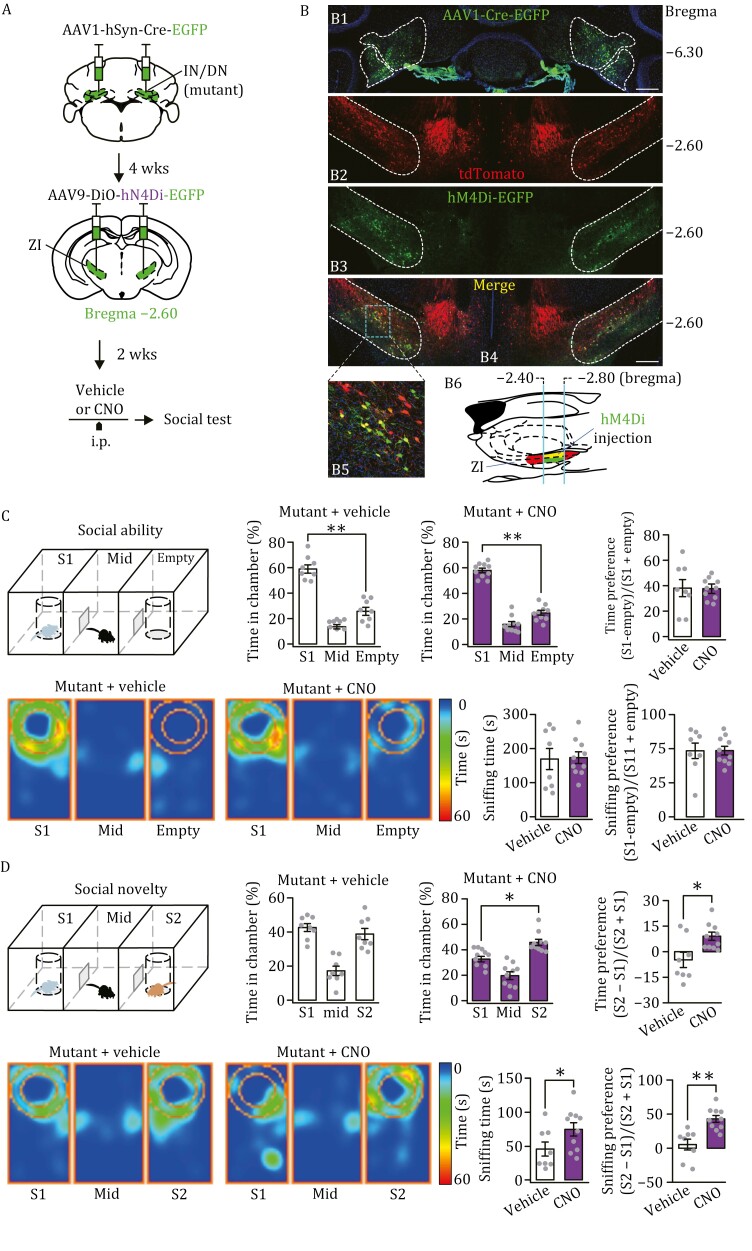
ZI^IN^ and ZI^DN^ neurons contribute to social novelty. (A) Experimental design in mutant mice using DREADD. AAV1-Cre-EGFP was injected into bilateral IN and DN of mutant mice at P21-25. Dio-hM4Di-EGFP was injected to the ZI of same mice four weeks later. Following a 2-weeks break, these mice were intraperitoneally injected with vehicle or CNO. (B) The expression of EGFP and Dio-hM4Di-EGFP. (B1) Bilateral IN and DN neurons (bregma −6.1) were infected by AAVI-Cre-EGFP. Scale bar, 200 μm. (B2) tdTomato^+^ neurons in bilateral ZI (bregma − 6.1). Scale bar, 200 μm. (B3) EGFP^+^ neurons in bilateral ZI (bregma −6.1), indicating that these neurons express hM4Di. Scale bar, 200 μm. (B4) The merge of (B2) and (B3). Boxed region is enlarged in (B5), and (B6) shows the injection site of hM4Di-EGFP in the ZI. (C) Social ability was examined in mutant mice treated with either vehicle or CNO. There was no difference between vehicle and CNO groups in example movement traces, spent time with S1, sniffing time onto S1, and sniffing preference (S1-empty). (D) Social novelty was examined in mutant mice treated with either vehicle or CNO. As shown by example movement traces, spent time with S2, sniffing time onto S2, and sniffing preference (S2–S1). Mutant mice treated with CNO exhibited more interest to S2 compared to mutant mice treated with vehicle. For statistics, see Table S8. *P < 0.05.

As shown by experimental scheme (Fig. 7A), mutant male mice were first microinjected bilaterally in the IN and DN with AAV1-hSyn-Cre-EGFP, and then microinjected bilaterally in posterodorsal ZI with a Cre-dependent inhibitory hM4Di designer receptor with an EGFP reporter (DIO-hM4Di-EGFP) 4 weeks later. After another 2-weeks recovery, these mice were injected with vehicle or the designer receptor agonist clozapine N-oxide (CNO; 1 mg/kg, i.p.) and subjected to electrophysiological and behavioral tests. Microinjections yielded strong EGFP signals in both IN and DN as well as mixed yellow signals mainly in posterodorsal ZI region (bregma −2.40 to −2.80), indicating that ZI^IN^ and ZI^DN^ neurons express hM4Di driven by Cre recombinase (Fig. 7B). In brain slices, CNO application hyperpolarized and decreased the firing frequency of hM4Di-expressing ZI^IN^/ZI^DN^ neurons, whereas control saline exerted no effect (Fig. S26A and S26B). We next evaluated whether chemogenetic inhibition of ZI^IN^ and ZI^DN^ neurons could rescue social defect in mutant mice. In the social approach test, mutant mice transduced with either vehicle or hM4Di did not differ in the preference to S1 (Fig. 7C). In the social novelty test, vehicle-treated mutant mice still demonstrated impaired social interaction, as shown by low preference to S2 (Fig. 7C). Introducing hM4Di significantly recovered the response of mutant mouse to social novelty, as shown by increased preference to S2 (Fig. 7D). The resident-intruder test was also performed in mutant mice receiving vehicle or CNO injection. We found a significant recovery in the exploration time for mutant mice with CNO injection in trial 2 (Fig. S5C and Table S10), whereas they showed no difference with mutant mice with vehicle injection in interacting with the novel mouse (Fig. S5D and Table S10). These results indicate that inhibiting ZI^IN^ and ZI^DN^ neurons rescues the defect in social novelty task of mutant mice. Furthermore, we subjected the mice to the open field test, and found that the inhibition of ZI^IN^ and ZI^DN^ neurons by transducing hM4Di did not affect mouse behavior in that test (Fig. S26B and Table S14). We investigated the consumption of sweet and regular foods as well as body weight in mutant mice with hM4Di expression and vehicle injection, compared to mutant mice with hM4Di expression and CNO injection. The results revealed no significant difference between the two groups (Fig. S3).

To further demonstrate the role of the IN/DN→ZI pathway in social behavior, we chemogenetically activated ZI^IN^ and ZI^DN^ neurons in control mice using DIO-hM3Dq-EGFP. As predicted, our results showed that chemogenetic activation of ZI^IN^ and ZI^DN^ neurons by CNO injection affected mouse behavior in the social novelty and resident-intruder tests. In the social approach test, control mice with activated ZI^IN^ and ZI^DN^ neurons had normal preference to S1 (Fig. S27A–C and Table S15). In the social novelty test, however, these mice demonstrated impaired social novelty, as shown by a lower preference to S2 (Fig. S27D–F and Table S15). In the resident-intruder test, we found a significant difference in the exploration time for the familiar mouse in control mice with CNO injection in trial 2, compared to vehicle injection mice (Fig. S28A and Table S15), whereas the same mice showed no difference in exploration time for the novel mouse (Fig. S28B and Table S15). Meanwhile, control mice with activated ZI^IN^ and ZI^DN^ neurons behaved normal in grooming test (Fig. S28C and Table S15).

To verify the functionality of the IN/DN→ZI pathway, we performed simultaneous optical fiber stimulation and microelectrode array recording at its axonal terminals (Fig. S29A). We injected anterograde and nontranssynaptic AAV2/9-hSyn-oChIEF-tdTomato into the IN/DN, and performed *in vivo* recordings three weeks later (Fig. S29B). By recording the local field potential (LFP), we found that light stimulation caused a robust activation of the ZI (Fig. S29C). Consistent with behavioral observations, *in vivo* recordings demonstrated that the LFP amplitude was increased in mutant mice compared to control mice (Fig. S29D). CNO, but not vehicle, significantly reduced the LFP amplitude of mutant mice expressing hM4Di (Fig. S29D).

Finally, to map axonal targets of ZI^IN^ and ZI^DN^ neurons, we expressed anterograde transsynaptic tracer AAV1-hSyn-Cre-EGFP in the IN and DN and another anterograde nontranssynaptic tracer (AAV9-DIO-EGFP) in the posterodorsal ZI of control mice (Fig. S30). We observed that ZI^IN^ and ZI^DN^ neurons projected to several cortical and striatal regions (caudate putamen, secondary somatosensory cortex, and ectorhinal cortex), thalamus (parafascicular thalamic nucleus, posterior thalamic nuclei and superior colliculus), PAG, and pontine reticular nucleus (Fig. S30). Notably, some regions receiving projections from ZI^IN^ and ZI^DN^ neurons are implicated in social functions, including the caudate putamen (Adorjan et al., 2017), ectorhinal cortex (Leung et al., 2018), and PAG (Chen et al., 2021; Tschida et al., 2019), which may explain why ZI^IN^ and ZI^DN^ neurons impact social novelty.

## Discussion

Many forms of autism have been associated with genetic mutations and/or deficits in cerebellar development, yet it remains to be elucidated whether and how autism genes can change the structural connectivity between brain regions, including that between the cerebellum and the cerebral cortex. In fact, “to what extent and how does the cerebellum play a role in nonmotor functions?” becomes a central question in the field of cerebellar research. Using large-scale transsynaptic tracing, we uncover that the pathways from the CN to the thalamus, midbrain and brainstem can be prominently affected by *Nlgn3*^R451C^ mutation. Moreover, the manipulation of CN→ZI pathway that aims at restoring the balance of neuronal activity improves the social deficit in mutant mice. These findings may have wide-spread implications for our insights on pathogenesis of both syndromic and nonsyndromic forms of autism.

### Aberrations in interregional connectivity as an underlying mechanism of cerebellar cognitive affective syndrome (CCAS)

*Nlgn3* ^R451C^ mutation may alter synaptic connections, including changed synaptic number or synaptic strength. For example, neuroligin 3 deficiency increases the number of climbing fiber-PC synapses (Baudouin et al., 2012). Furthermore, the changes in synaptic connections may affect the extent of transsynaptic infection (Hoche et al., 2018). Therefore, we are able to observe the alteration in the number of target neurons.

For the pathogenesis of autism, most mechanistic studies have been focusing on two respects: (i) how heritable or *de novo* mutations can affect synaptic development and function; and (ii) which brain region and how neuronal activity may be implicated in social disorders. From another point of view, our data show that the structural alteration of the cerebellar outputs may be paramount and with widespread differences in *Nlgn3*^R451C^ mice, and manipulating the activity in part of these pathways turns out to affect social behavior.

Given the protracted developmental timeline of the cerebellum, Sathyanesan et al. (2019) have proposed that abnormal cerebellar development may contribute to neurodevelopmental disorders, including not only intellectual disability, attention-deficit hyperactivity disorder and Down syndrome but also autism. More specifically, they advocate that over- or under-connectivity of the cerebellum with efferent targets may lead to high- or low-functioning forms of autism, respectively. The potential clinical relevance of such bidirectional alterations in structural connectivity are also supported by the studies in patients suffering from CCAS (Hoche et al., 2018): structural MRI studies reveal that their cerebello-cerebral connections can be either increased (Khan et al., 2015; Pierce et al., 2004) or decreased (Jack and Morris, 2014; Mostofsky et al., 2009).

Our work suggests that structural changes in the output of the cerebellum have functional implications in social behaviors. This notion is supported by studies using optogenetic stimulation of the cerebellum in combination with field recording of cerebral cortex (Lindeman et al., 2021; Witter et al., 2013), as well as by functional MRI studies revealing a coupling between the cerebellum and the cerebral cortex at rest and/or during sensorimotor or cognitive control (Abdelgabar et al., 2019; Buckner, 2013; Stoodley and Schmahmann, 2009). Hence, it appears possible that structural aberrations in the development of cerebellar outputs will lead to functional deficits observed in autism (Wang et al., 2014). On one hand, we observed that ZI^IN^ neurons project to nuclei associated with social interaction, such as the striatum, entorhinal cortex, and PAG, which may account for abnormal ZI^IN^ neuronal activity leading to abnormal social interaction. Therefore, future research should examine the projections of ZI^CN^→Cpu/Ect/PAG in *Nlgn3*^R451C^ mice. On the other hand, we also found that *Nlgn3*^R451C^ mutation altered CN→VTA projection, which has been linked to social behaviors (Carta et al., 2019). All these pathways may be involved in the circuit mechanisms underlying how aberrant cerebellar outputs lead to social deficits.

It will be also interesting to find out whether the aberrations in cerebellar outputs are present in cerebellar cell-specific mutations, such as that in *Tsc1*^PC^ mice (Tsai et al., 2012). Recently, Gibson et al. (2023) demonstrate that stimulating the right Crus1 of *Fmr1* knockout mice, a model of syndromic autism (Baudouin et al., 2012), is enough to rescue social defects. This work indicates the potential role of cerebellar outputs in syndromic autism with global genetic mutation, and thereby supports the scientific implications of the present study using *Nlgn3*^R451C^ mice.

### Role of CN projection to posterodorsal ZI in social interaction

Using transsynaptic tracing, we demonstrate that each of the CN projects with a particular density to a specific subpopulation of neurons in the ZI. Interestingly, *Nlgn3*^R451C^ mutation mainly affects a subpopulation aggregated at the posterodorsal part of the ZI. Inhibiting ZI^IN^ and ZI^DN^ neurons turns out to be sufficient to rescue impaired social novelty-seeking behavior in *Nlgn3*^R451C^ mice. Possibly, this subpopulation of cells partly coincides with inhibitory tachykinin-expressing ZI neurons that are recently found to instigate novelty-seeking behavior (Ahmadlou et al., 2021). Our disynaptic tracing shows that ZI^IN^ and ZI^DN^ neurons project to the contralateral striatum, somatosensory cortex, entorhinal cortex, thalamic nuclei, superior colliculus, peri-aquaductal gray and/or pontine reticular nucleus, several of which may be involved in control of social behavior (Adorjan et al., 2017; Chen et al., 2021; Chou et al., 2018; Leung et al., 2018; Tschida et al., 2019; Wang et al., 2019). To explore potential new approach to treat the social aspects of autism, future studies will have to identify which of these downstream targets of ZI are critical for the pathogenesis of these symptoms.

### Differential effects of *Nlgn3*^R451C^ mutation on CN efferents

*Nlgn3* ^R451C^ mutation differentially affects the projections from the FN, IN, and DN to thalamus, midbrain, and brainstem in that the densities of the projections are different in 36 nuclei. Which mechanisms may contribute to these differential effects? First, neuroligin 3 is a cell adhesion molecule situated in the postsynaptic membrane of both excitatory and inhibitory inputs (Tabuchi et al., 2007). CN-targeted neurons in the thalamus, midbrain and brainstem may differentially express neuroligin 3 in a brain region-dependent manner (Rothwell et al., 2014; Uchigashima et al., 2021). Thus, neuroligin 3 may act both in an anterograde manner at the dendritic sites of CN neurons and in a retrograde manner at the terminal sites (Futai et al., 2013; Südhof, 2017). Second, neuroligin 3 may be involved in translational processes. On one hand, neuroligin 3 is subject to posttranslational modifications, which in turn affect the specification of synaptic contacts and thereby the organization of the network (Uchigashima et al., 2021). On the other hand, neuroligin 3 itself may regulate the translation of protein signaling (Hornberg et al., 2020). Finally, neuroligin 3 may also remodel neuronal circuits by acting on the turnover of spines. For example, *Nlgn3*^R451C^ mice show enhanced dynamics of PSD95-positive spines in the somatosensory cortex (Isshiki et al., 2014) and impaired elimination of redundant climbing fiber-PC synapses (Lai et al., 2021). However, our data show that the *Nlgn3*^R451C^ mutation does not affect the morphology of PCs and CN neurons, implying differentiation in the possible roles of neuroligin 3 in the development of distinct brain regions. Therefore, there are ample potential molecular and cellular mechanisms that may contribute to the differential changes of CN connectivity in *Nlgn3*^R451C^ mouse model.

### Implications of the present study on ASD and other neurodevelopmental disorders

In summary, we uncover structural aberrations in the CN→thalamic, the CN→midbrain and the CN→brainstem pathways in *Nlgn3*^R451C^ mice, which promote our understanding of circuitry mechanisms by which cerebellar dysfunction leads to the defects in social interactions. Understanding cerebello-cerebral topographies in the context of neurodevelopmental disorders will enable targeted applications through both pharmacological and stimulation-based interventions. Future research should proceed to investigate structural changes in cerebellar outputs in other syndromic and nonsyndromic autism mouse models.

The diverse neural circuit disorders in different subtypes of autism must be acknowledged. Previous research has demonstrated impaired CN→VM circuit in *Tsc1*^PC^ mice (Kelly et al., 2020), but this alteration is not observed in *Nlgn3*^R451C^ mice. Therefore, understanding structural changes in the brains of individuals with autism is crucial for developing effective treatment strategies. The development of neural circuits involves processes such as neuronal proliferation, differentiation, and migration. It is evident that environmental and genetic factors can disrupt normal development of neural circuits, leading to neurodevelopmental disorders. For example, E12.5 marks the final stage of PC generation (Inouye and Murakami, 1980). However, exposure to valproic acid (VPA) at this time point leads to the loss of PCs (Al Sagheer et al., 2018; Wang et al., 2018) and increases the risk of autistic-like behaviors in mouse offspring (Zarate-Lopez et al., 2024), underscoring the significance of disruptions in cerebellar-related circuits in neurodevelopmental disorders. In conclusion, large-scale circuit tracing can be applied to neurodevelopmental disorders, revealing at a cellular level the changes in circuits that underlie these diseases and their heterogeneity.

## Supplementary Material

pwae040_suppl_Supplementary_Figures

pwae040_suppl_Supplementary_Tables

pwae040_suppl_Supplementary_File

pwae040_suppl_Supplementary_Movies

## Data Availability

Due to upload limitations, we have submitted the coordinates of all traced neurons (a zipped file containing 36 excel files), all movie files, and uncompressed figures for Figure 1 onto an eternal and public web storage. Please contact the corresponding author for detailed information. Other source data supporting the findings of this study are available from the lead contact upon reasonable request.
