# Supplementary material for: Aberrant outputs of cerebellar nuclei and targeted rescue of social deficits in an autism mouse model
Source: Protein Cell. 2024 Jul 27;15(12):872–88. doi: 10.1093/procel/pwae040 (PMC11637611; doi:10.1093/procel/pwae040)
Supplement: pwae040_suppl_Supplementary_Figures [file pwae040_suppl_supplementary_figures.pdf]

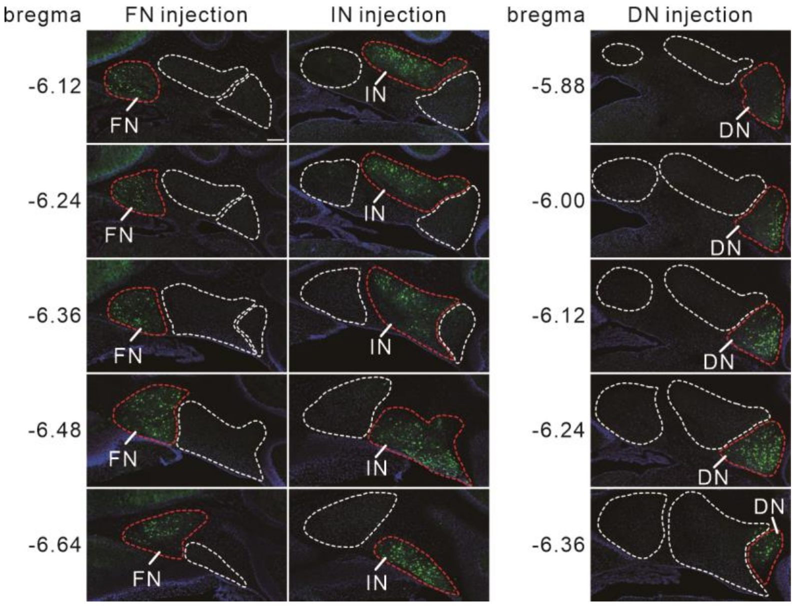


**Figure S1. Infection specificity in FN, IN and DN.** Slice imaging at annotated bregma levels show labeled neurons ($GFP^+$) following AAV1 injection in FN, IN and DN, indicating that neurons were specifically labeled in each nuclues. Scale bar, 200 μm.

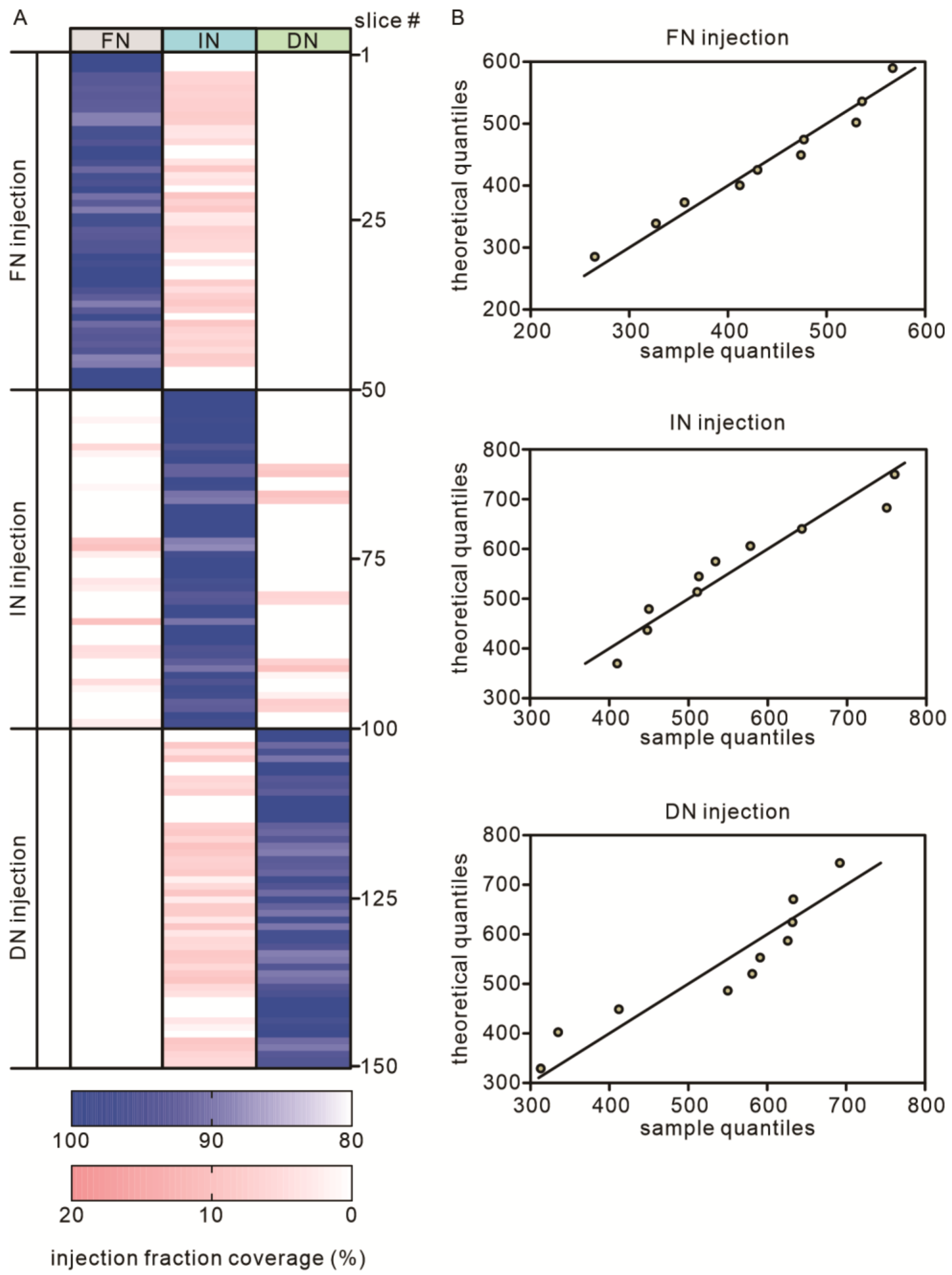


**Figure S2. Specificity and consistency of viral injection in Ai9 mice.** (A) Heat map derived from 150 slices (30 mice at 5 bregma levels) shows the percentages of infection coverage (infected area/total nuclear area) in FN, IN and DN. FN injection: 95.1 ± 0.5% (FN), 4.7 ± 0.5% (IN), 0 (DN). IN injection: 1.3 ± 0.4% (FN), 96.9 ± 0.5% (IN), 1.7 ± 0.5% (DN). DN injection: 0 (FN), 5.1 ± 0.5% (IN), 94.7 ± 0.6% (DN). (B) Q-Q plots indicate the normal distribution of data points. Statistical analysis using the Shapiro-Wilk test: FN, $P$ = 0.76 ($n$ = 10); IN, $P$ = 0.25 ($n$ = 10) and DN, $P$ = 0.07 ($n$ = 10).

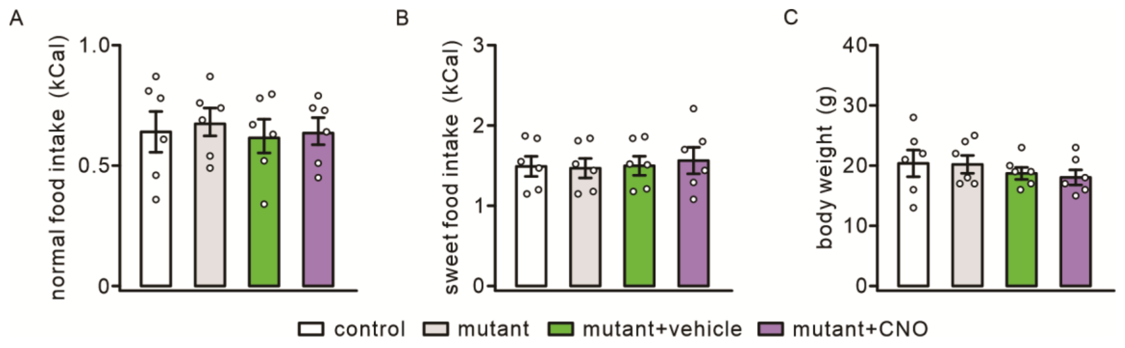


**Figure S3. Manipulation of IN/DN→ZI pathway does not affect food intake**. (A) *Nlgn3*$^{R451C}$ mutation or chemogenetic inhibition of IN/DN→ZI pathway did not alter intake of regular food over 1 hour. control: 0.65 ± 0.07 kCal (*n* = 6; *P* = 0.38 compared to mutant; *P* = 0.41 compared to mutant+vehicle; *P* = 0.48 compared to mutant+CNO). mutant: 0.68 ± 0.05 kCal (*n* = 6; *P* = 0.27 compared to mutant+vehicle; *P* = 0.32 compared to mutant+CNO). mutant+vehicle: 0.62 ± 0.06 kCal (*n* = 6; *P* = 0.41 compared to mutant+CNO). mutant+CNO: 0.64 ± 0.05 kCal (*n* = 6). One-way Anova test. (B) *Nlgn3*$^{R451C}$ mutation or chemogenetic inhibition of IN/DN→ZI pathway did not alter intake of sweet food over 1 hour. control: 1.51 ± 0.11 kCal (*n* = 6; *P* = 0.45 compared to mutant; *P* p = 0.49 compared to mutant+vehicle; *P* = 0.37 compared to mutant+CNO). mutant: 1.49 ± 0.10 kCal (*n* = 6; *P* = 0.43 compared to mutant+vehicle; *P* = 0.32 compared to mutant+CNO). mutant+vehicle: 1.52 ± 0.10 kCal (*n* = 6; *P* = 0.37 compared to mutant+CNO). mutant+CNO: 1.59 ± 0.14 kCal (*n* = 6). One-way Anova test. (C) *Nlgn3*$^{R451C}$ mutation or chemogenetic inhibition of IN/DN→ZI pathway did not affect body weight. control: 20.7 ± 1.9 g (*n* = 6; *P* = 0.48 compared to mutant; *P* = 0.25 compared to mutant+vehicle; *P* = 0.19 compared to mutant+CNO). mutant: 20.5 ± 1.3 g (*n* = 6; *P* = 0.21 compared to mutant+vehicle; *P* = 0.15 compared to mutant+CNO). mutant+vehicle: 19.0 ± 0.9 g (*n* = 6; *P* = 0.34 compared to mutant+CNO). mutant+CNO: 18.3 ± 1.1 g (*n* = 6).

One-way Anova test.

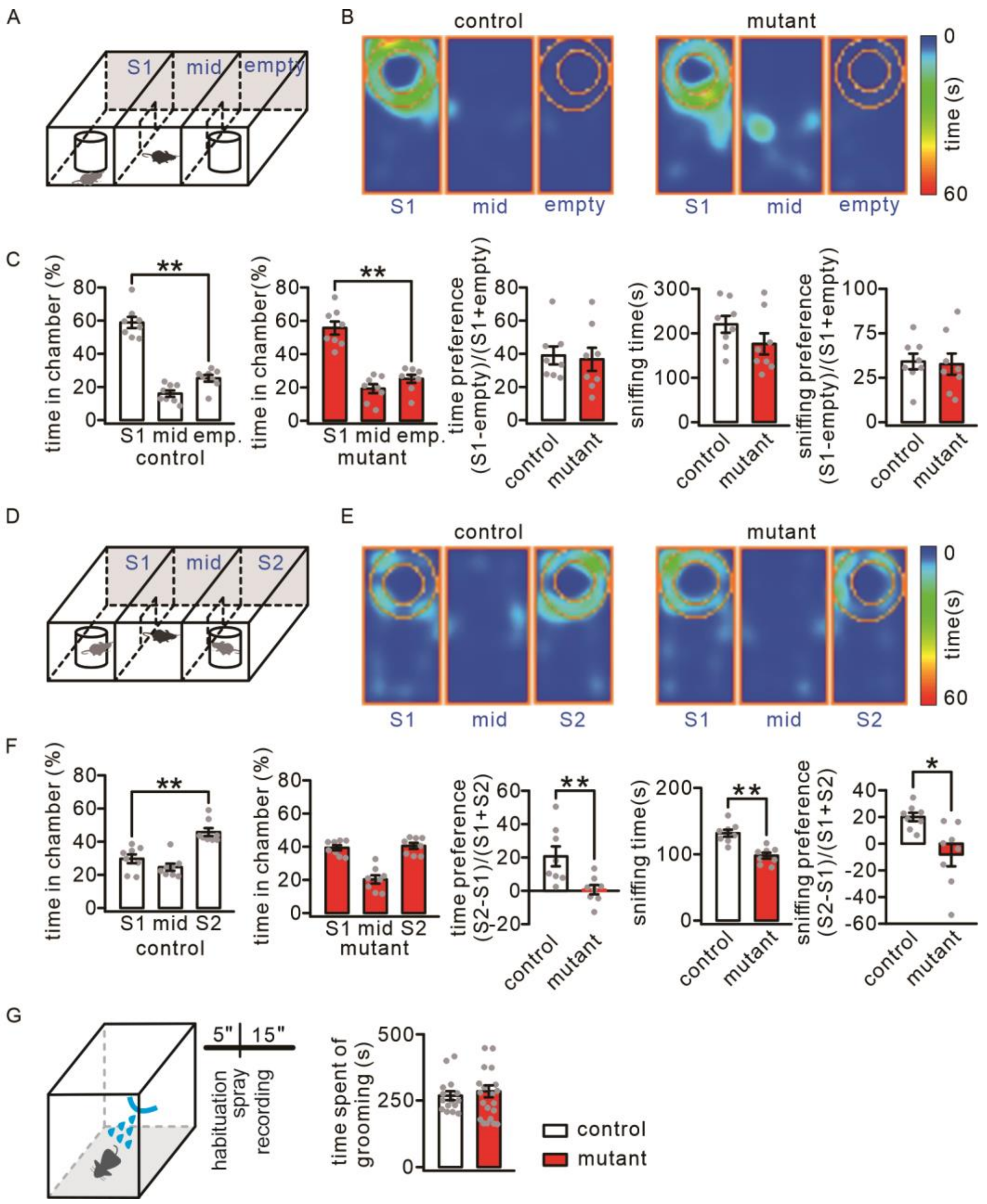


**Figure S4. Impaired social novelty in *Nlgn3*$^{R451C}$ mutant mice.** (A) Configuration of a three-chamber social interaction. (B) Heat maps showing movement traces of control and mutant mice. (C) The averages of spent times in S1, middle (mid), and empty chambers. emp.: empty. (D) Social novelty test following the introduction of a second stranger (S2). (E) Heat maps showing movement traces of control and mutant mice after the introduction of S2. (F) Statistics show that Mutant mice showed no interest to S2. *$P < 0.05$. **$P < 0.01$. (G) Cumulative time spent engaged in water spray-induced grooming behavior was

scored over a 15-min session. Mice were allowed a 5-min habituation before the test. Mutant mice displayed no increase in repetitive grooming. For statistics, see Table S9.

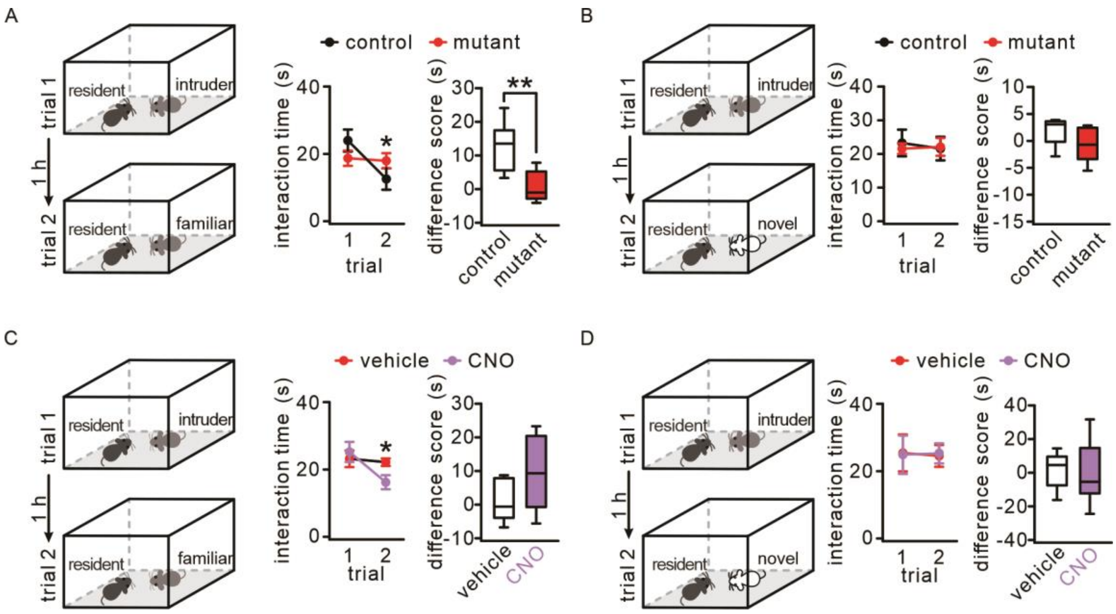


**Figure S5. Impaired social interaction of *Nlgn3*$^{R451C}$ mutant mice in the resident-intruder test.** (A) Intruder mouse was used in two consecutive trials with a familiar mouse. Mutant mice failed to display a decreased investigation time during trial 2. (B) Intruder mouse was used in two trials with a novel mouse. Control and mutant groups explored two different mice similarly. (C) Resident-Intruder tests for mutant mice receiving vehicle or CNO. (F) Vehicle and CNO groups explored two different mice similarly. For statistics, see Table S10.

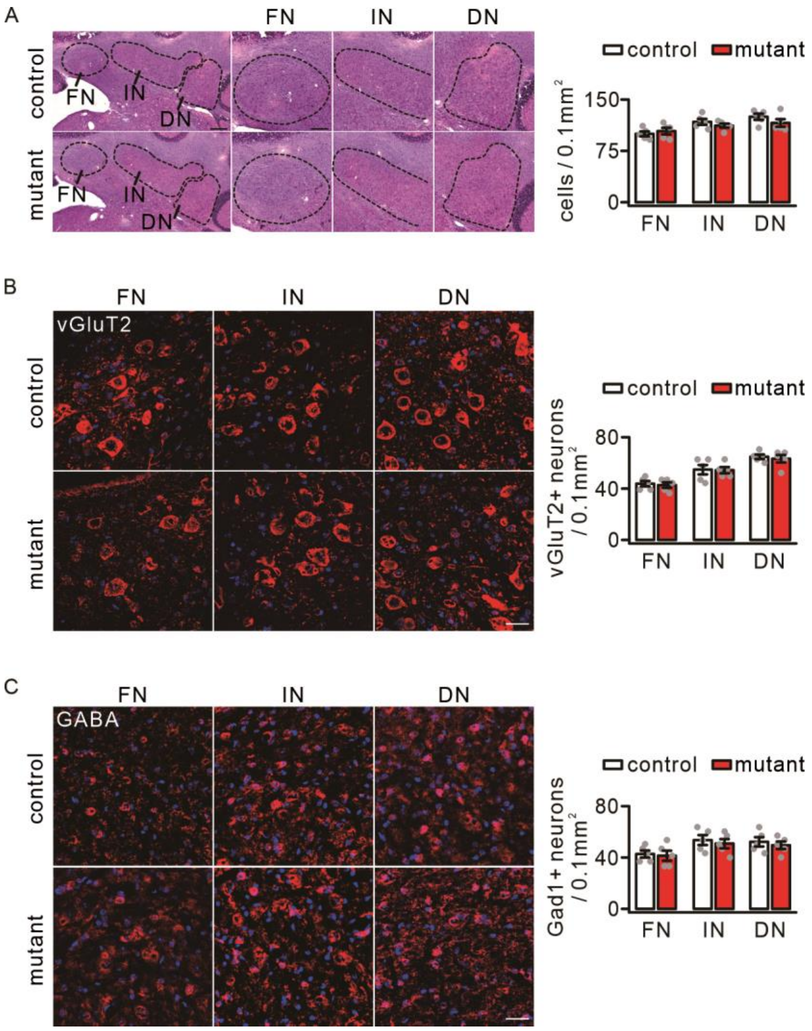


**Figure S6. The *Nlgn3*$^{R451C}$ mutation does not affect number of CN neurons.** (A) H&E staining in the CN from control and mutant mice. Scale bars, 200 μm. (B) Immunostaining for vGluT2, showing that the numbers of vGluT2$^{+}$ neurons were not changed by *Nlgn3*$^{R451C}$ mutation. Scale bars, 25 μm. (C) Immunostaining for GABA, showing that the numbers of GABA$^{+}$ neurons were not changed by *Nlgn3*$^{R451C}$ mutation. Scale bars, 25 μm. For statistics, see Table S11.

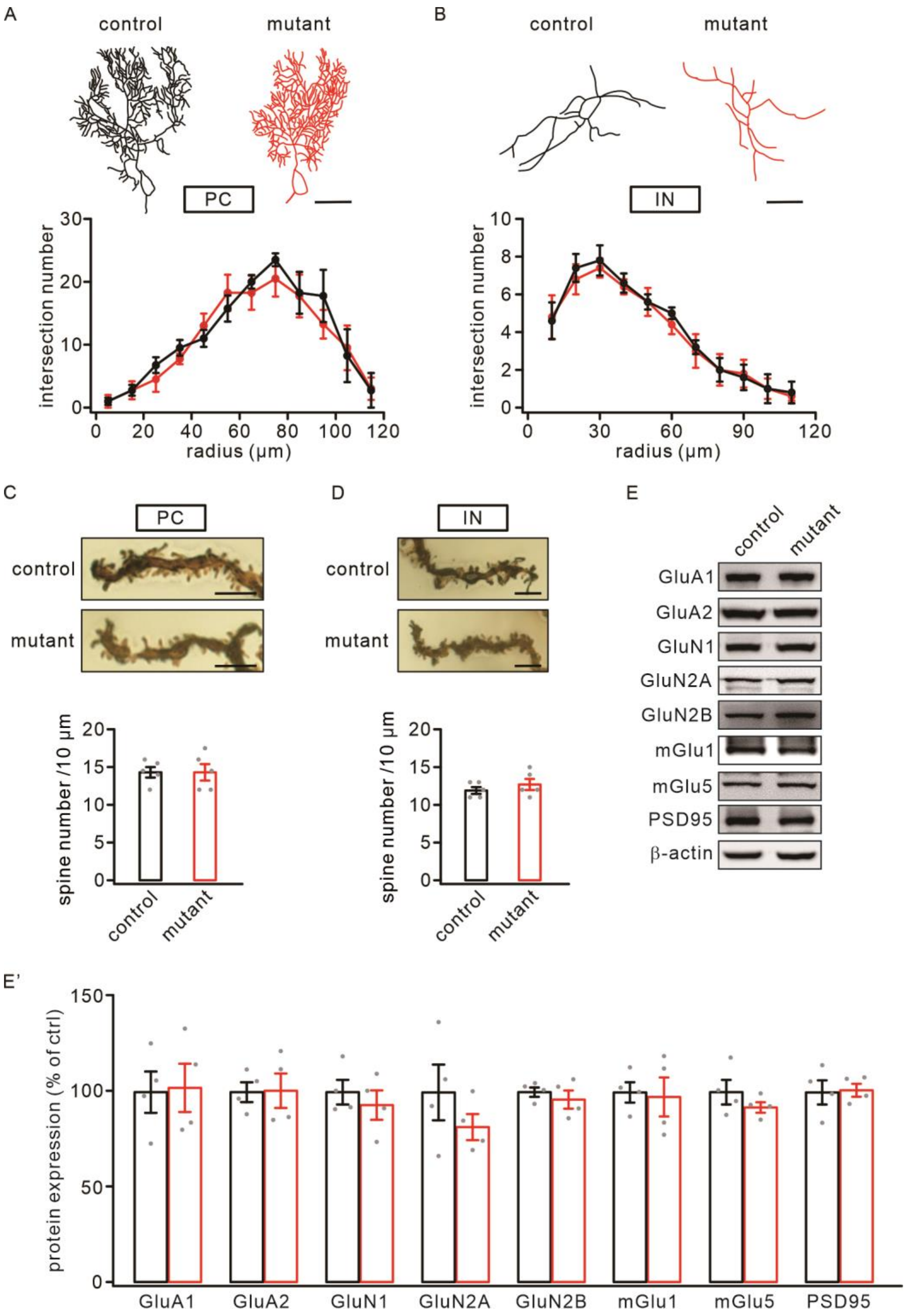


**Figure S7. *Nlgn3*$^{R451C}$ mutation does not affect neuronal morphology in the cerebellum.** (A) Analysis of PC dendritic trees from control and mutant mice. Scale bars, 500 μm. Sholl analysis showed no change in the radius and intersection number in mutant mice. (B) Analysis of PC dendritic trees. Scale bars, 500 μm. Sholl analysis showed no

change in the radius and intersection number in mutant mice. (C) Golgi staining showing apical PC spines in control and mutant mice. (D) Golgi staining showing IN spines in control and mutant mice. (E) Cerebellar PSD fractions from control and mutant mice were probed with antibodies to GluA1, GluA2, GluN1, GluN2A, GluN2B, mGlu1, mGlu5, and PSD95. β-actin was internal the control. (E') Histograms show percentage changes of proteins in mutant mice relative to the control. For statistics, see Table S11.

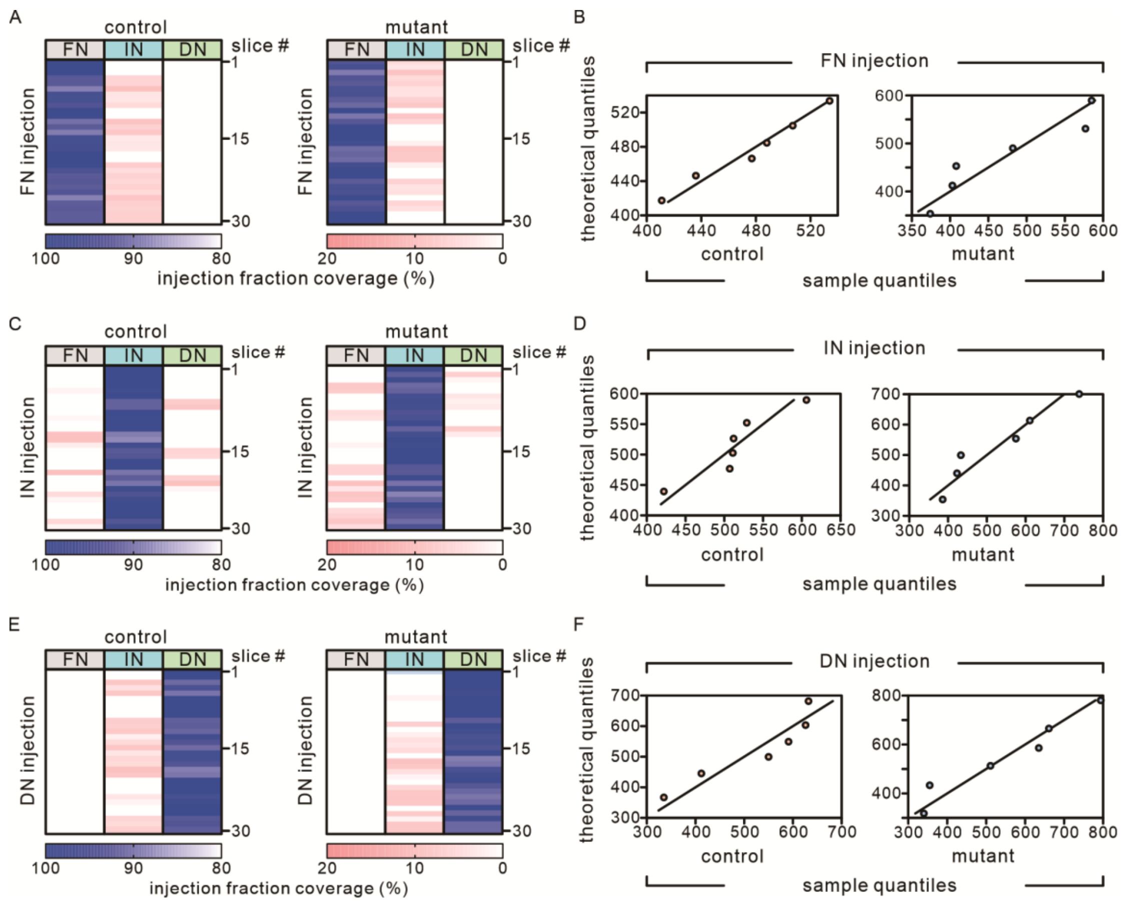


**Figure S8. Specificity and consistency of viral injection in control and mutant mice.**

(A) Percentages of infection coverage following viral injection in the FN. *n* = 30 slices for each group (5 bregma levels). In control, 95.2 ± 0.6% (FN), 5.1 ± 0.6% (IN), 0 (DN). In mutant, 95.7 ± 0.6% (FN), 4.3 ± 0.6% (IN), 0 (DN). (B) Q-Q plots indicate the normal distribution of data points. *P* values were 0.90 (control; *n* = 6) and 0.20 (mutant; *n* = 6). (C) Percentages of infection coverage following the injection in the IN. *n* = 30 slices for each. In control, 1.7 ± 0.6% (FN), 96.7 ± 0.7% (IN), 1.6 ± 0.6% (DN). In mutant, 3.3 ± 0.7% (FN), 95.8 ± 0.6% (IN), 0.9 ± 0.4% (DN). (D) Q-Q plots indicate the normal distribution of data points. *P* values were 0.38 (control; *n* = 6) and 0.44 (mutant; *n* = 6). (E) Percentages of infection coverage following the injection in the DN. *n* = 30 slices for each. In control, 0 (FN), 96.0 ± 0.7% (IN), 0 (DN). In mutant, 0 (FN), 3.8 ± 0.7% (IN), 96.2 ± 0.7% (DN). (F)

Q-Q plots indicate the normal distribution of data points. *P* values were 0.19 (control; *n* = 6) and 0.57 (mutant; *n* = 6).

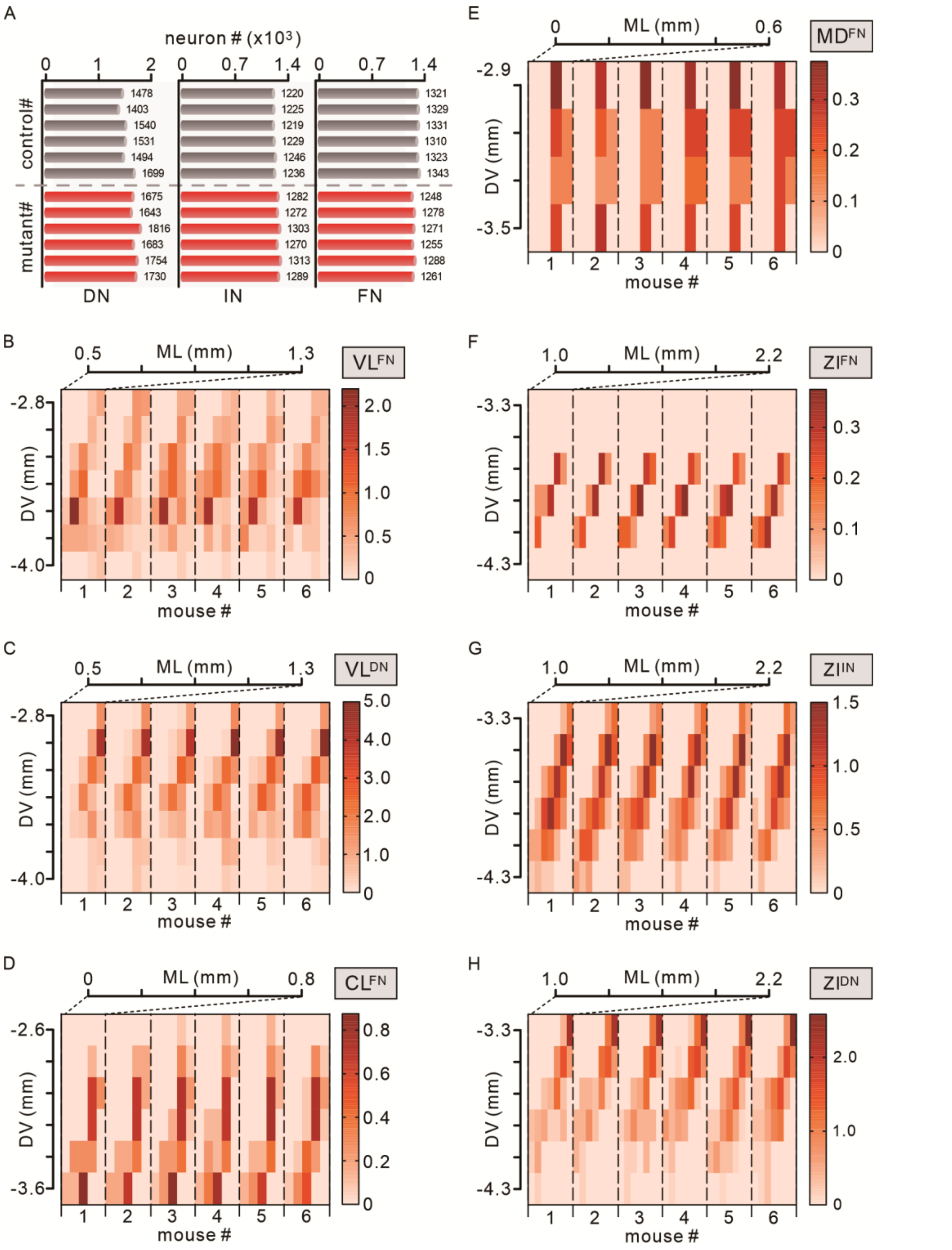


**Figure S9. Consistency of neuronal subpopulation analysis among 6 mutant mice.**

(A) Numbers of neurons used for subpopulation analysis. (B-H) Heat maps showing percentage numbers of $VL^{FN}$ neurons (bregma –1.22). (B), $VL^{DN}$ neurons (bregma –1.22) (C), $CL^{FN}$ neurons (bregma –1.34) (D), $MD^{FN}$ neurons (bregma –1.70) (E), $ZI^{FN}$ neurons (bregma –2.54) (F), $ZI^{IN}$ neurons (bregma –2.54) (G), and $ZI^{DN}$ neurons (bregma –2.54)

(H) in 6 mutant mice, as separated by dashed lines.

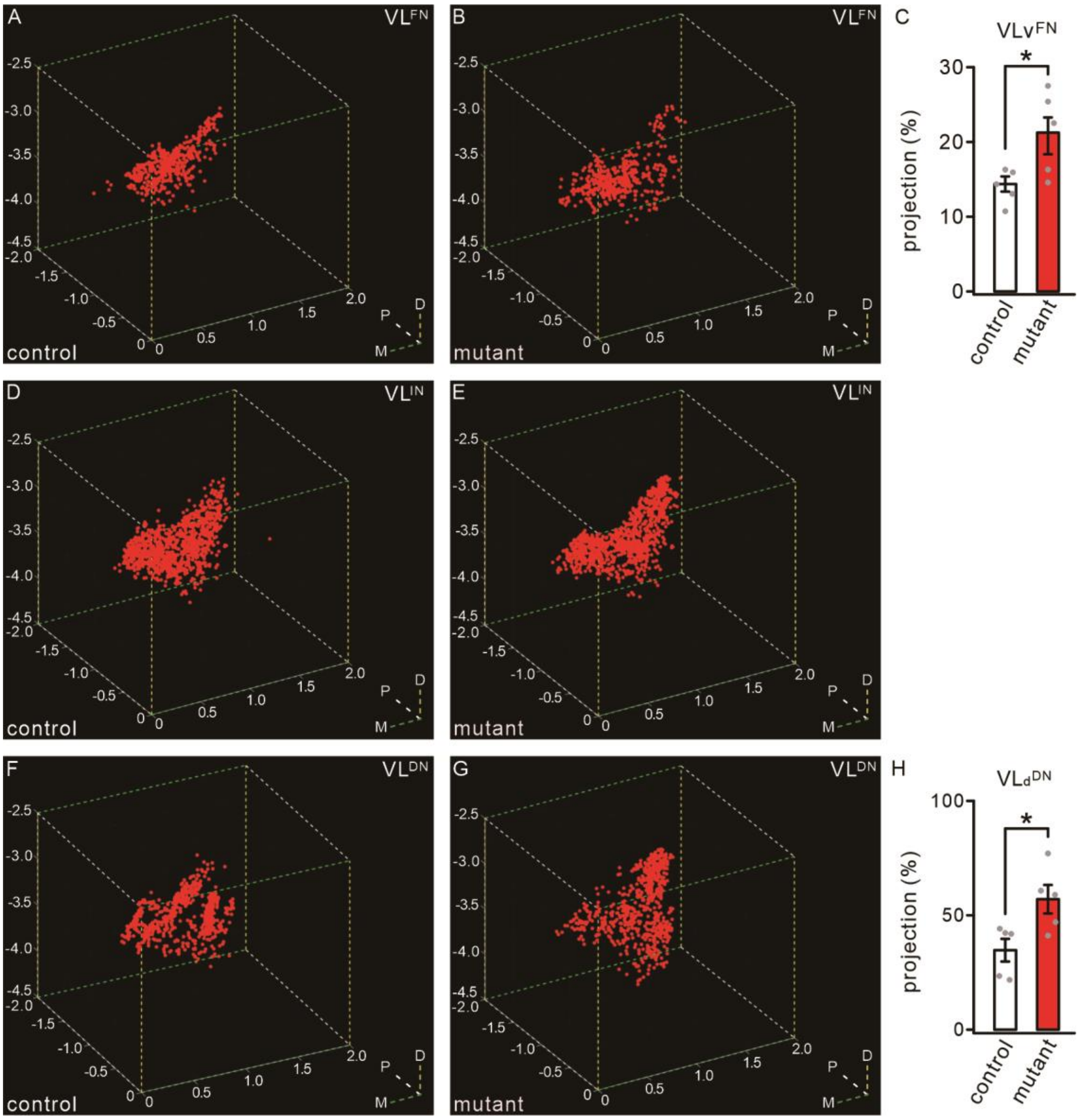


**Figure S10. 3D Distribution of VL$^{FN}$, VL$^{IN}$ and VL$^{DN}$ neurons.** (A, B) VL$^{FN}$ neurons in a control mouse and a mutant mouse. (C) % projections of VL$_v^{FN}$ neurons. (D, E) VL$^{IN}$ neurons in a control mouse and a mutant mouse. (F, G) VL$^{DN}$ neurons in a control mouse and a mutant mouse. (H) % projections of VL$_d^{DN}$ neurons. For statistics, see Table S12.

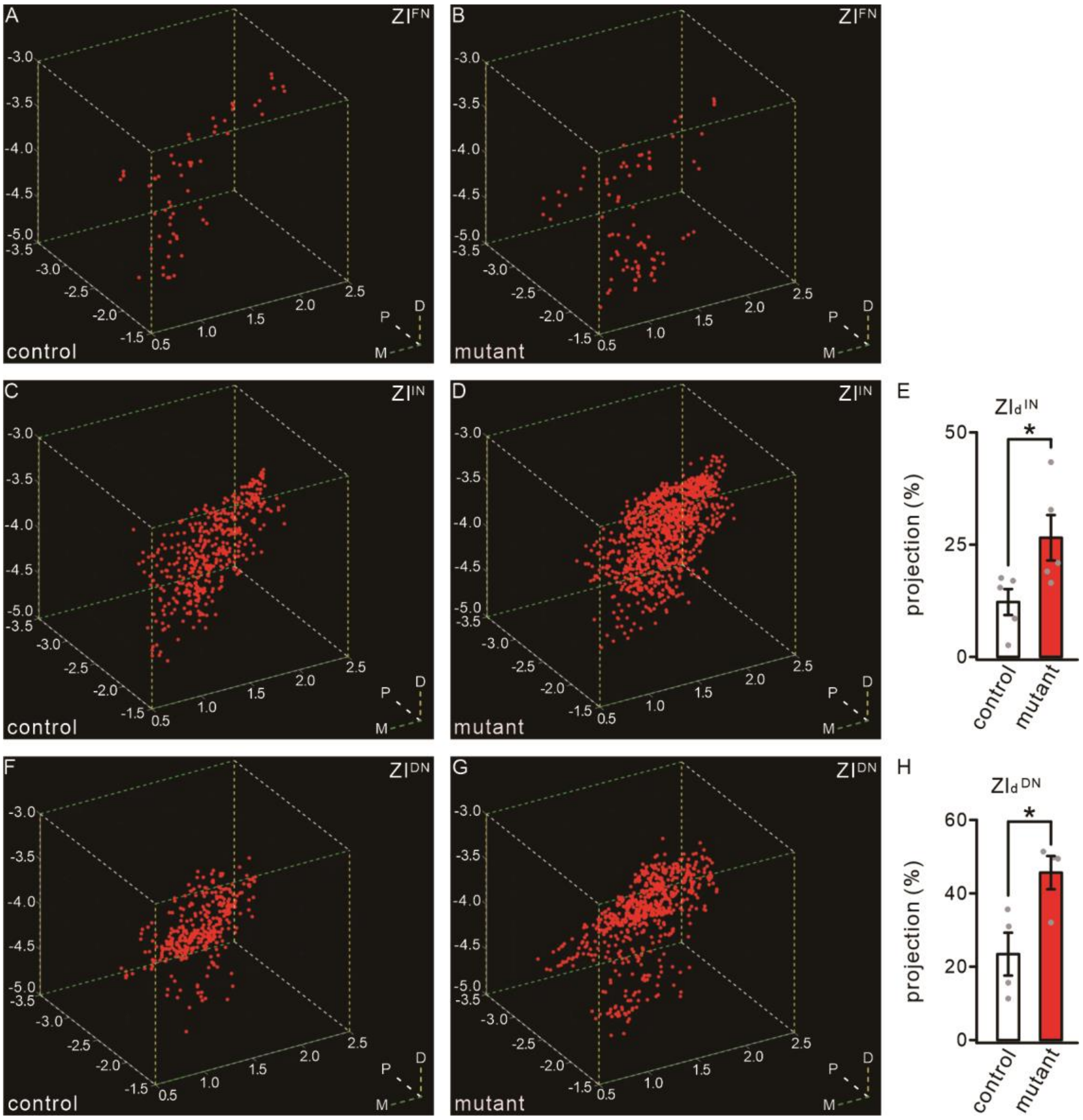


**Figure S11. 3D Distribution of $ZI^{FN}$, $ZI^{IN}$ and $ZI^{DN}$ neurons.** (A, B) $ZI^{FN}$ neurons in a control mouse and a mutant mouse. (C, D) $ZI^{IN}$ neurons in a control mouse and a mutant mouse. (E) % projections of $ZI_{pd}^{IN}$ neurons. (F, G) $ZI^{DN}$ neurons in a control mouse and a mutant mouse. (H) % projections of $ZI_{pd}^{DN}$ neurons. For statistics, see Table S12.

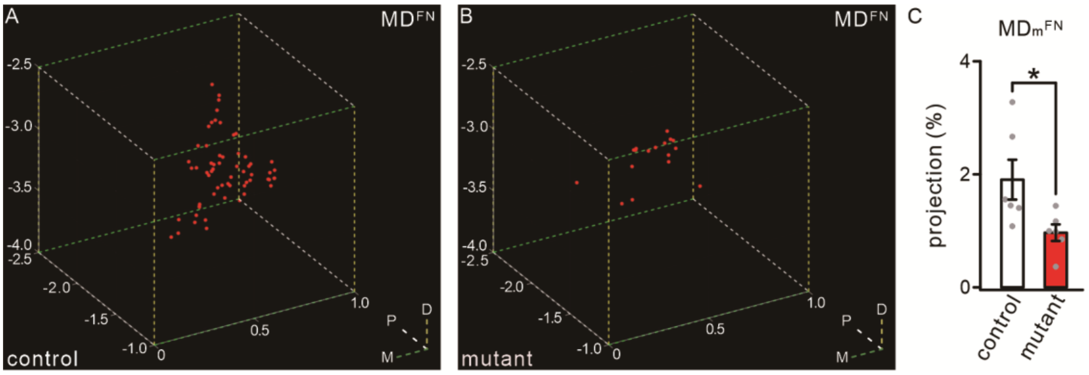


**Figure S12. 3D Distribution of $MD^{FN}$ neurons.** (A, B) $MD^{FN}$ neurons in a control mouse and a mutant mouse. (C) % projections of $MD_m^{FN}$ neurons. For statistics, see Table S12.

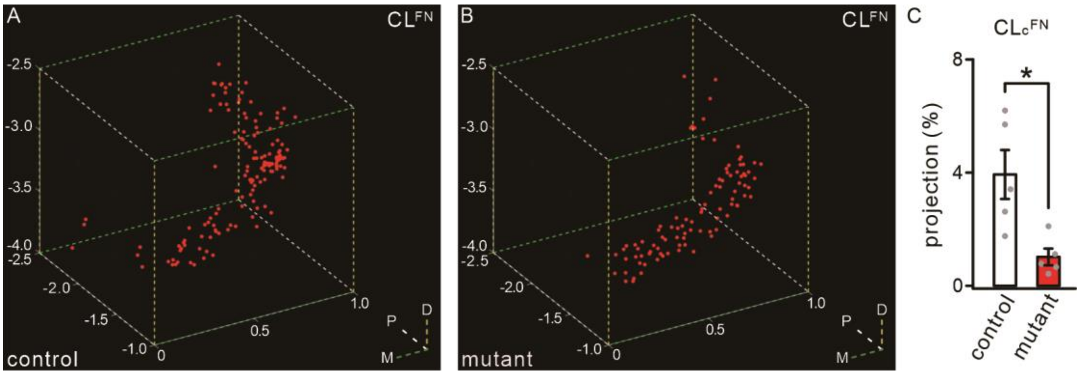


**Figure S13. 3D Distribution of CL$^{FN}$ neurons.** (A, B) CL$^{FN}$ neurons in a control mouse and a mutant mouse. (C) % projections of CL$_c^{FN}$ neurons. For statistics, see Table S12.

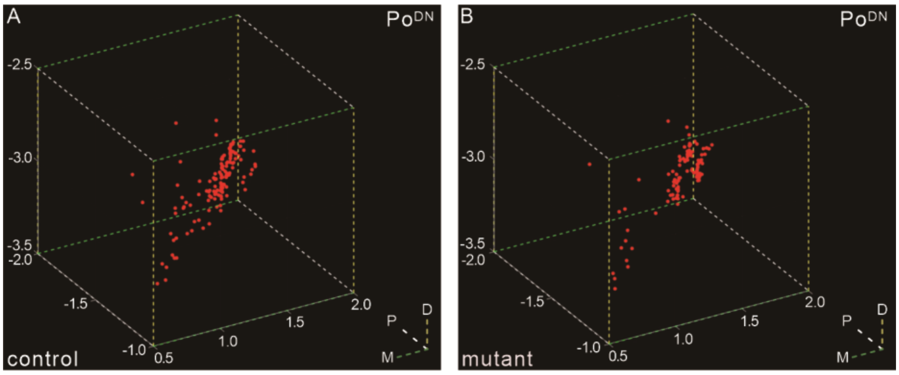


**Figure S14. 3D Distribution of Po$^{DN}$ neurons.** (A) Po$^{DN}$ neurons in a control mouse. (B) Po$^{DN}$ neurons in a mutant mouse.

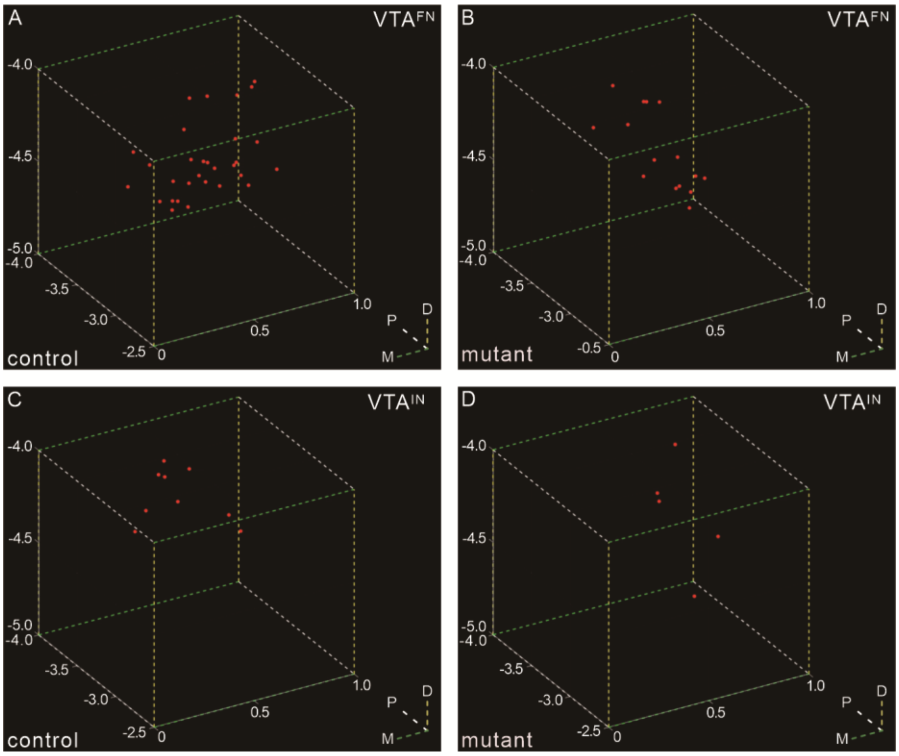


**Figure S15. 3D Distribution of VTA$^{FN}$ and VTA$^{IN}$ neurons.** (A, B) VTA$^{FN}$ neurons in a control mouse and a mutant mouse. (C, D) VTA$^{IN}$ neurons in a control mouse and a mutant mouse.

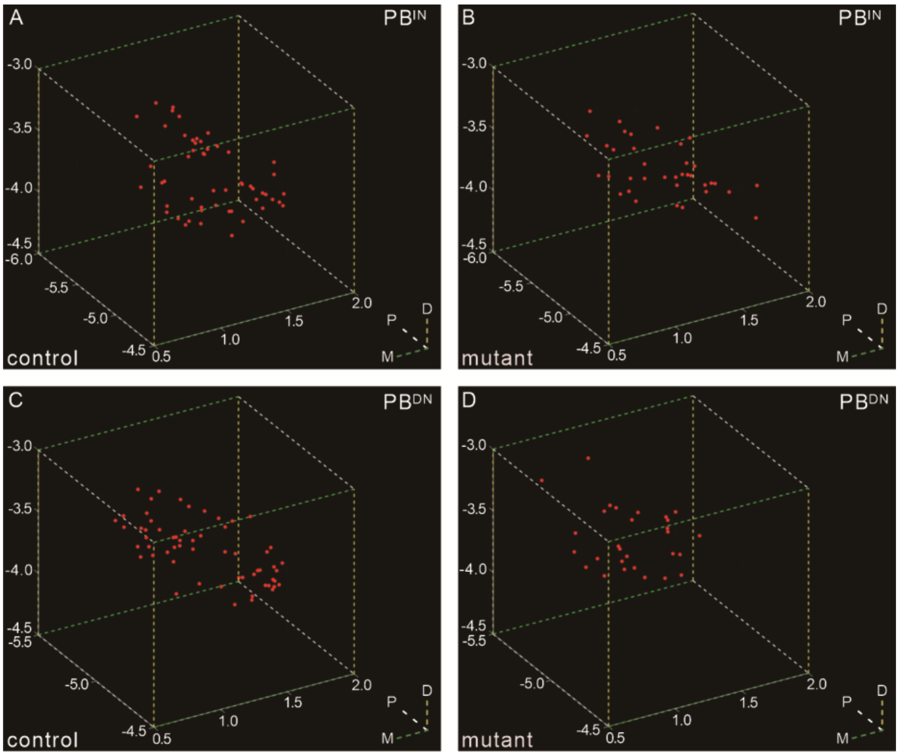


**Figure S16. 3D Distribution of PB$^{IN}$ and PB$^{DN}$ neurons.** (A, B) PB$^{IN}$ neurons in a control mouse and a mutant mouse. (C, D) PB$^{DN}$ neurons in a control mouse and a mutant mouse.

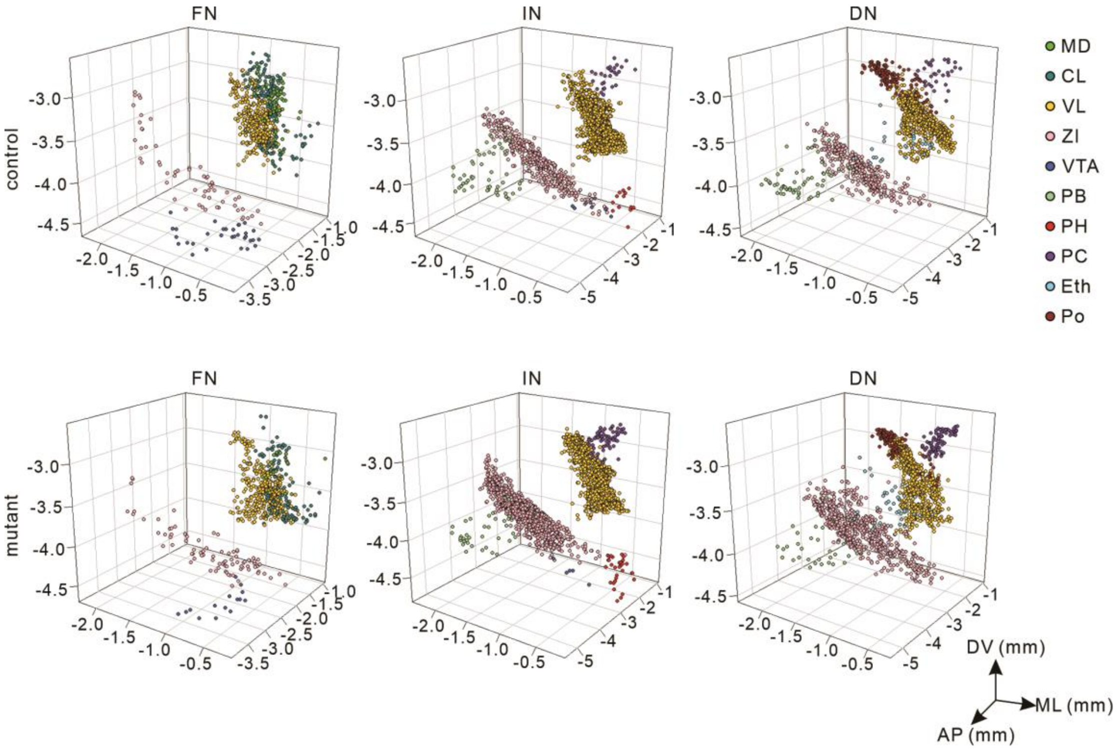


**Figure S17. 3D Subdivisions of anterogradely labeled neurons in 10 nuclei.** The somata of neurons innervated with FN, IN and DN outputs were reconstructed in 3D space. Upper: 3D subdivision of labeled neurons in 10 nuclei (MD, CL, VL, ZI, VTA, PB, PH, PC, Eth, and Po) from a control mouse. This experiment was repeated in 6 control mice. Lower: 3D subdivision of labeled neurons in same 10 nuclei from a mutant mouse. This experiment was repeated in 6 mutant mice. Note that subdivisional changes of labeled neurons caused by *Nlgn3*$^{R451C}$ mutation were different among nuclei.

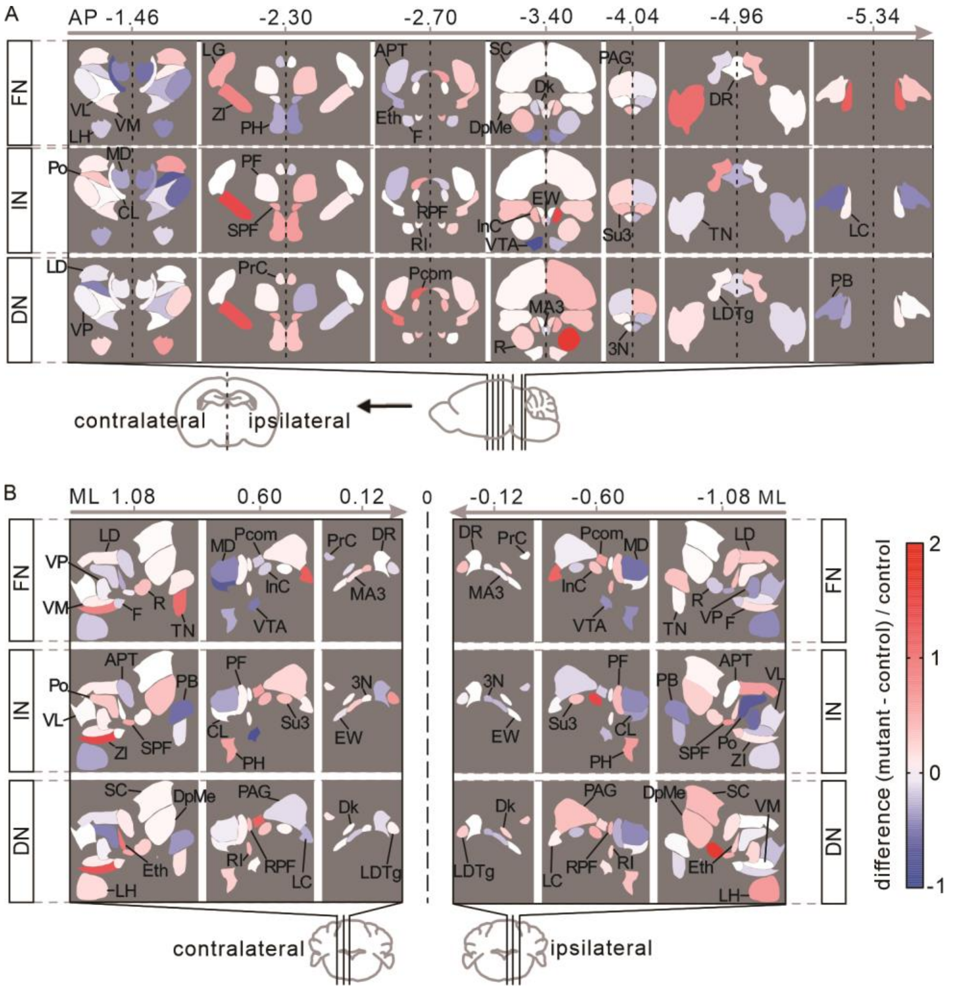


**Figure S18. Mapping differences in traced neuron counts onto nuclei.** (A) Mapping normalized differences ((mutant-control)/control) in neuron counts onto target nuclei depicted at annotated bregma levels. Dashed lines indicate the midline of the brain. (B) Mapping normalized differences onto target nuclei depicted at annotated mediolateral (ML) levels. Left: controlateral. Right: ipsilateral.

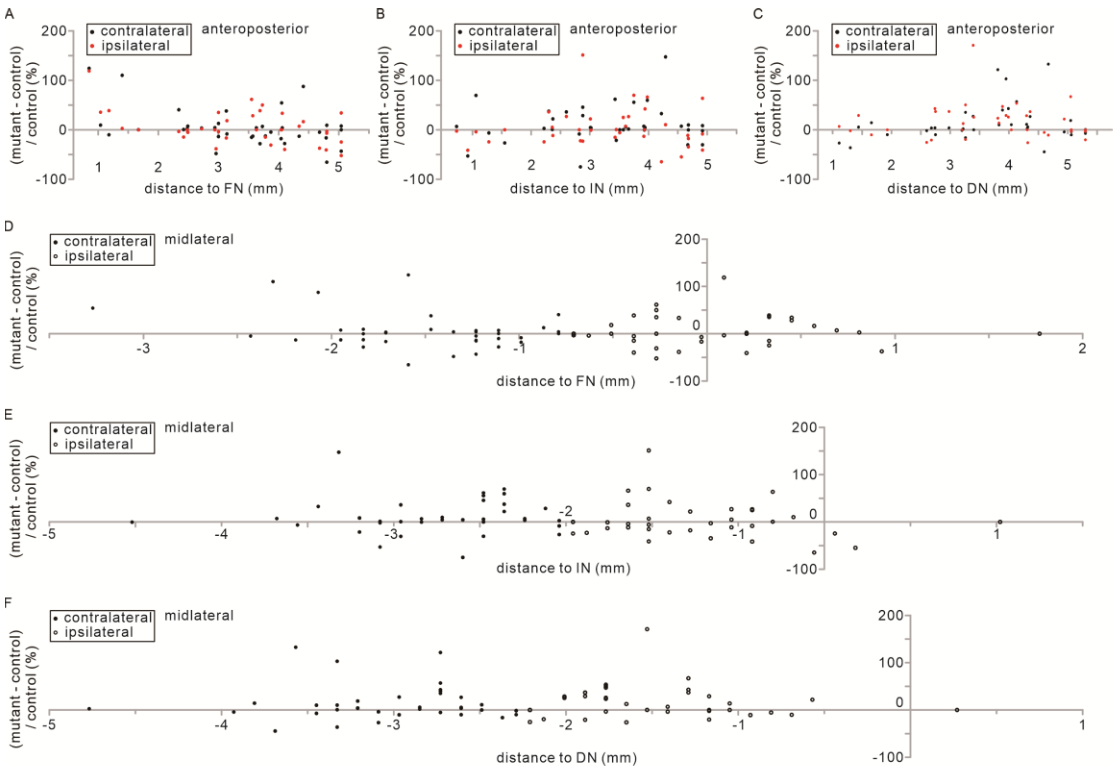


**Figure S19. Relationship analysis between differences in neuron counts and nuclear distances to CN.** (A-C) Normalized differences ((mutant-control)/control) in neuron counts of contralateral (black) and ipsilateral (red) nuclei were scatter plotted to their distances from FN (A), IN (B) and DN (C) in the anteroposterior direction. (D-F) Normalized differences in neuron counts of contralateral (filled) and ipsilateral (unfilled) nuclei were scatter plotted to their distances from FN (D), IN (E), and DN (F) in the mediolateral direction.

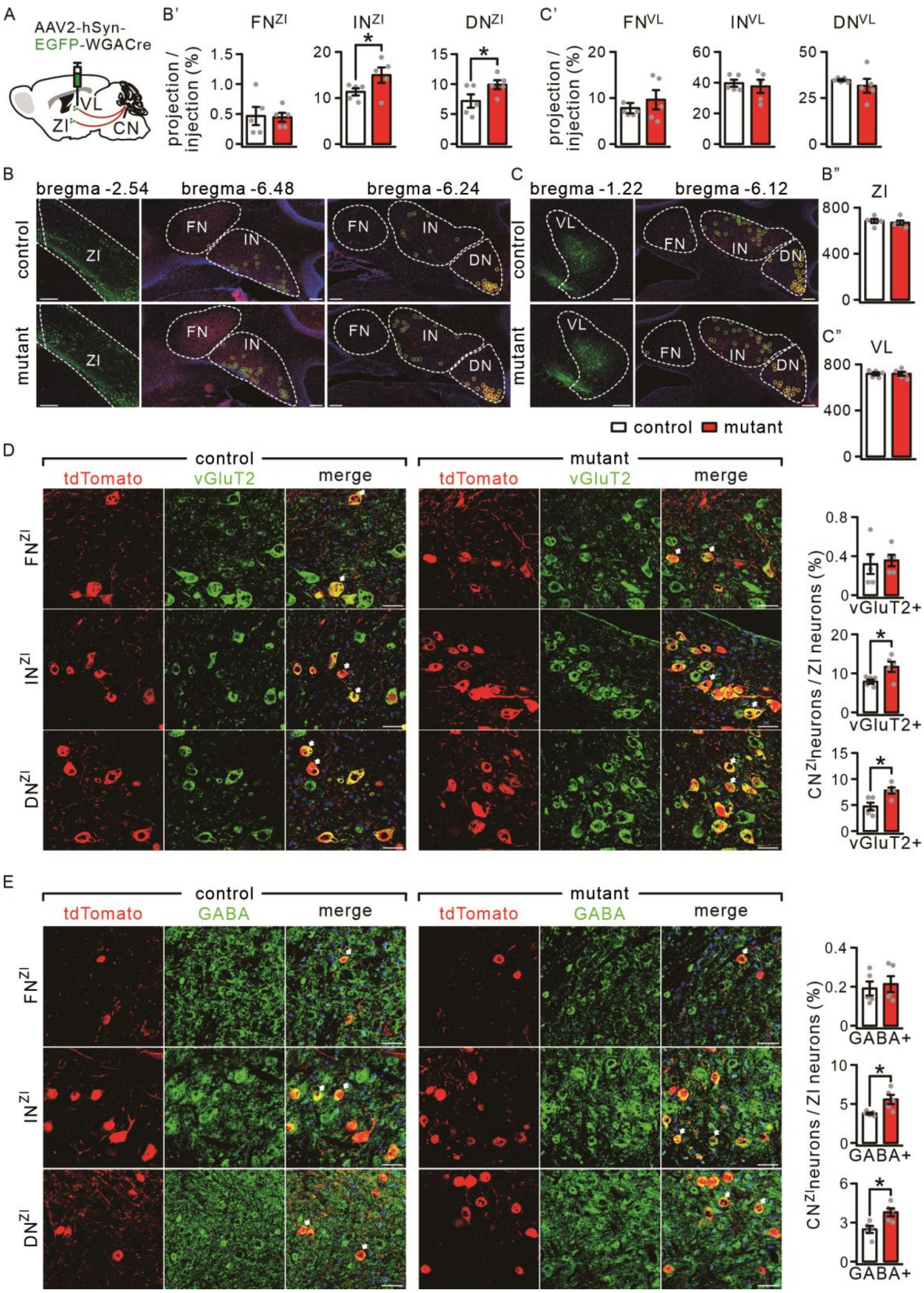


**Figure S20. Retrograde transsynaptic tracing from VL and ZI to CN.** (A) AAV2 containing EGFP-WGA-Cre was injected into VL or ZI. Infected neurons were labeled with EGFP and traced neurons were labeled with tdTomato. (B) Left: EGFP-labeled neurons in

ZI (bregma –2.54). Middle and right: retrogradely traced neurons in FN, IN and DN (bregma –6.48 and –6.24). Scale bars, 200 μm. (B’) Percentages of traced neurons/GFP-labeled ZI neurons. (B”) Numbers of labeled neurons: control: 862 ± 44 ($n$ = 5); mutant: 844 ± 47 ($n$ = 5); $P$ = 0.58, unpaired $t$ test. (C) Left: EGFP-labeled neurons in VL (bregma –1.22). Right: retrogradely traced neurons in FN, IN and DN (bregma –6.12). Scale bars, 200 μm. control: $n$ = 5. mutant: $n$ = 5. (C’) Percentages of traced neurons/GFP-labeled VL neurons. (C”) Numbers of labeled neurons: control: 724 ± 27 ($n$ = 5); mutant: 725 ± 36 ($n$ = 5); $P$ = 0.98, unpaired $t$ test. (D) $FN^{ZI}$, $IN^{ZI}$ and $DN^{ZI}$ neurons (tdTomato$^{+}$) were fluorescently labeled by vGluT2. Scale bars: 40 μm. The bar graphs illustrate the proportions of $FN^{ZI}$, $IN^{ZI}$ and $DN^{ZI}$ neurons (tdTomato$^{+}$ and vGluT2$^{+}$) relative to GFP-labeled ZI neurons. (E) $FN^{ZI}$, $IN^{ZI}$ and $DN^{ZI}$ neurons (tdTomato$^{+}$) were fluorescently labeled by GABA. Scale bars: 40 μm. Bar graphs illustrate the proportions of $FN^{ZI}$, $IN^{ZI}$ and $DN^{ZI}$ neurons (tdTomato$^{+}$ and GABA$^{+}$) relative to GFP-labeled ZI neurons. For statistics, see Table S13. *$P$ < 0.05.

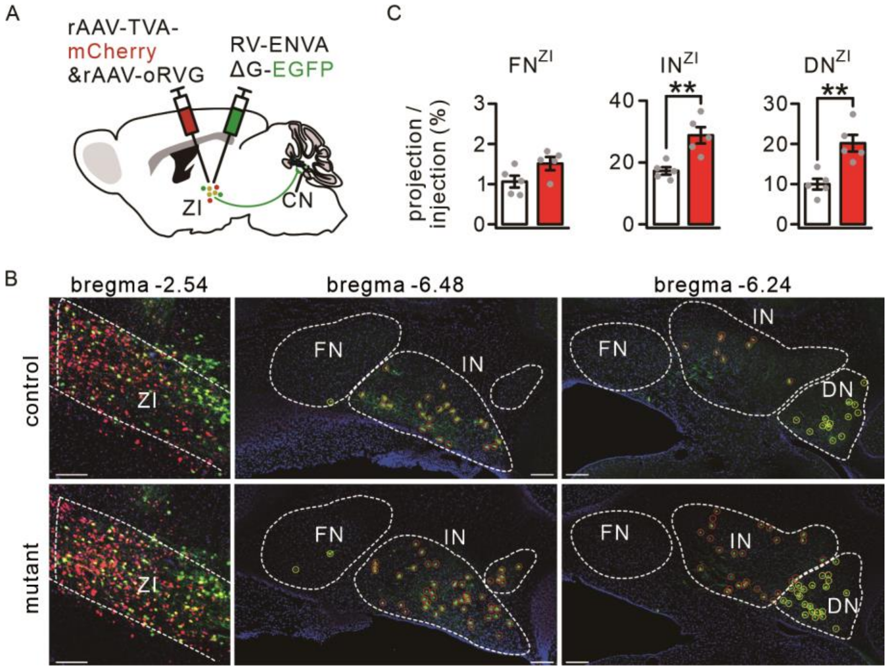


**Figure S21. RV-based retrograde transsynaptic tracing from ZI to CN.** (A) EGFP-containing RV-ENVA-ΔG, rAAV-TVA-mCherry and rAAV-oRVG were injected into ZI. Infected neurons were labeled with EGFP and mCherry, while retrogradely labeled CN neurons were labeled with EGFP. (B) Left: EGFP and tdTomato-labeled neurons in ZI (bregma –2.54). Middle and right: retrogradely traced neurons in FN, IN and DN (bregma –6.48 and –6.24). Scale bars, 200 μm. (C) Percentages of traced neurons/ mCherry-labeled ZI neurons. For statistics, see Table S14. *$P$ < 0.05. **$P$ < 0.01.

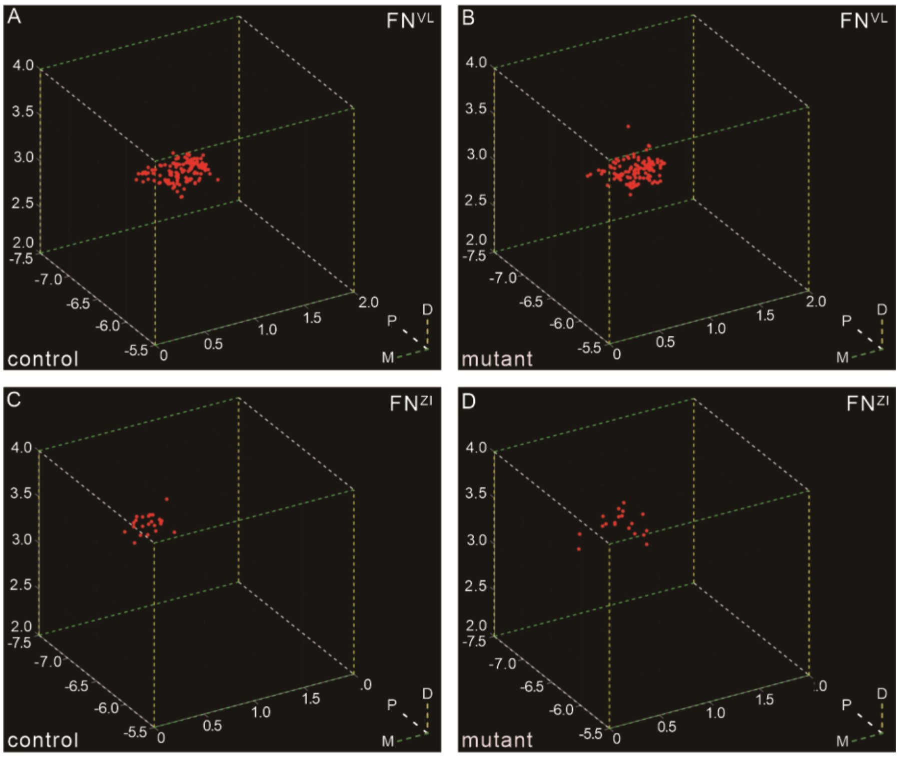


**Figure S22. 3D distribution of retrogradely labeled FN neurons.** (A, B) $FN^{VL}$ neurons in a control mouse and a mutant mouse. (C, D) $FN^{ZI}$ neurons in a control mouse and a mutant mouse.

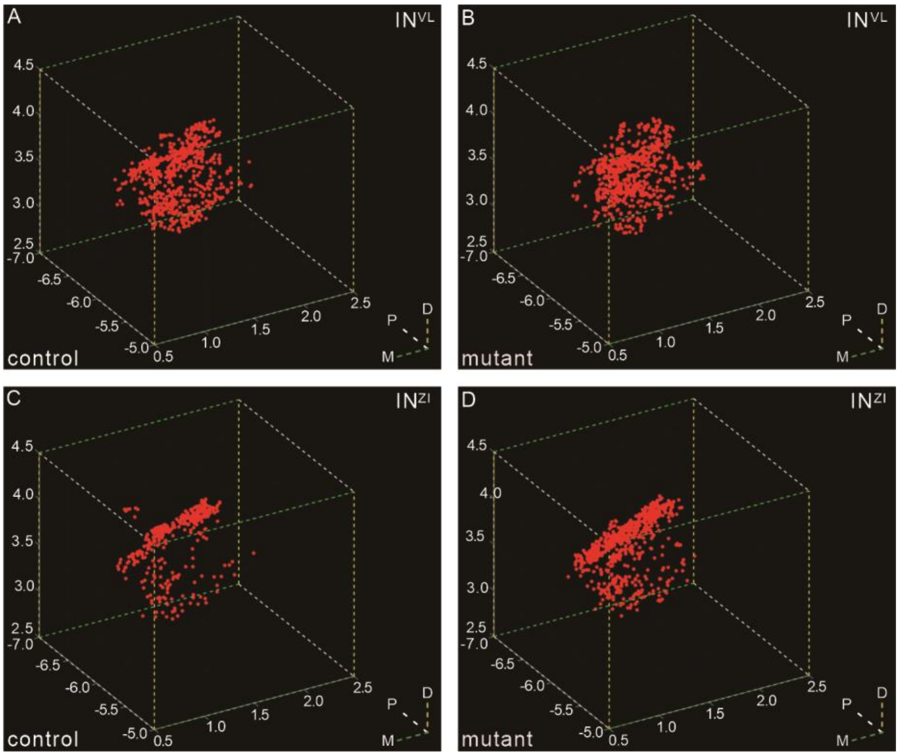


**Figure S23. 3D distribution of retrogradely labeled IN neurons.** (A, B) IN$^{VL}$ neurons in a control mouse and a mutant mouse. (C, D) IN$^{ZI}$ neurons in a control mouse and a mutant mouse.

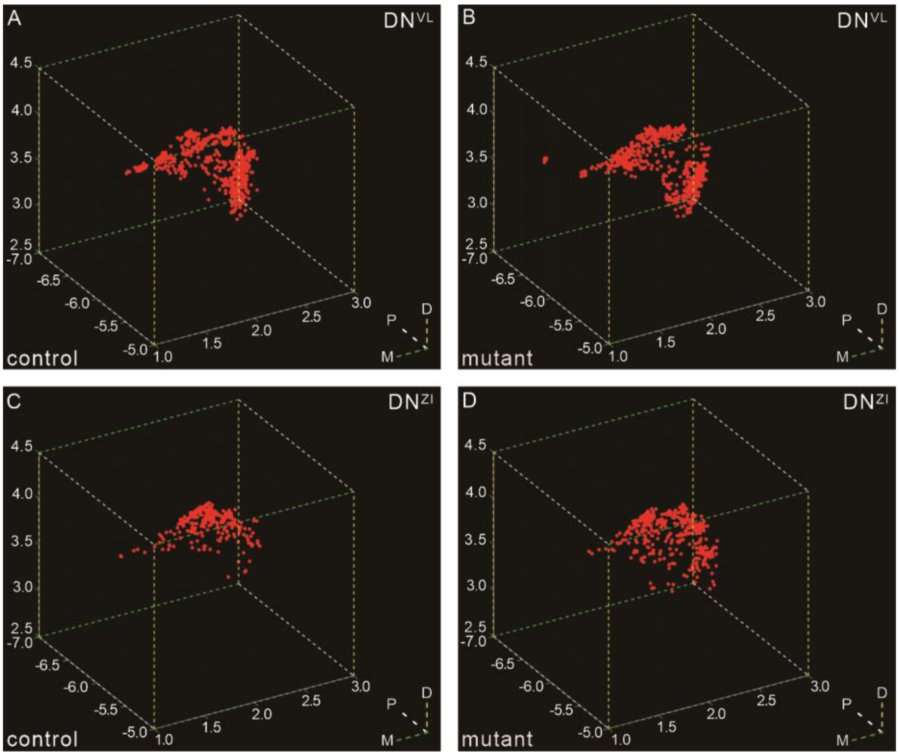


**Figure S24. 3D distribution of retrogradely labeled DN neurons.** (A, B) $DN^{VL}$ neurons in a control mouse and a mutant mouse. (C, D) $DN^{ZI}$ neurons in a control mouse and a mutant mouse.

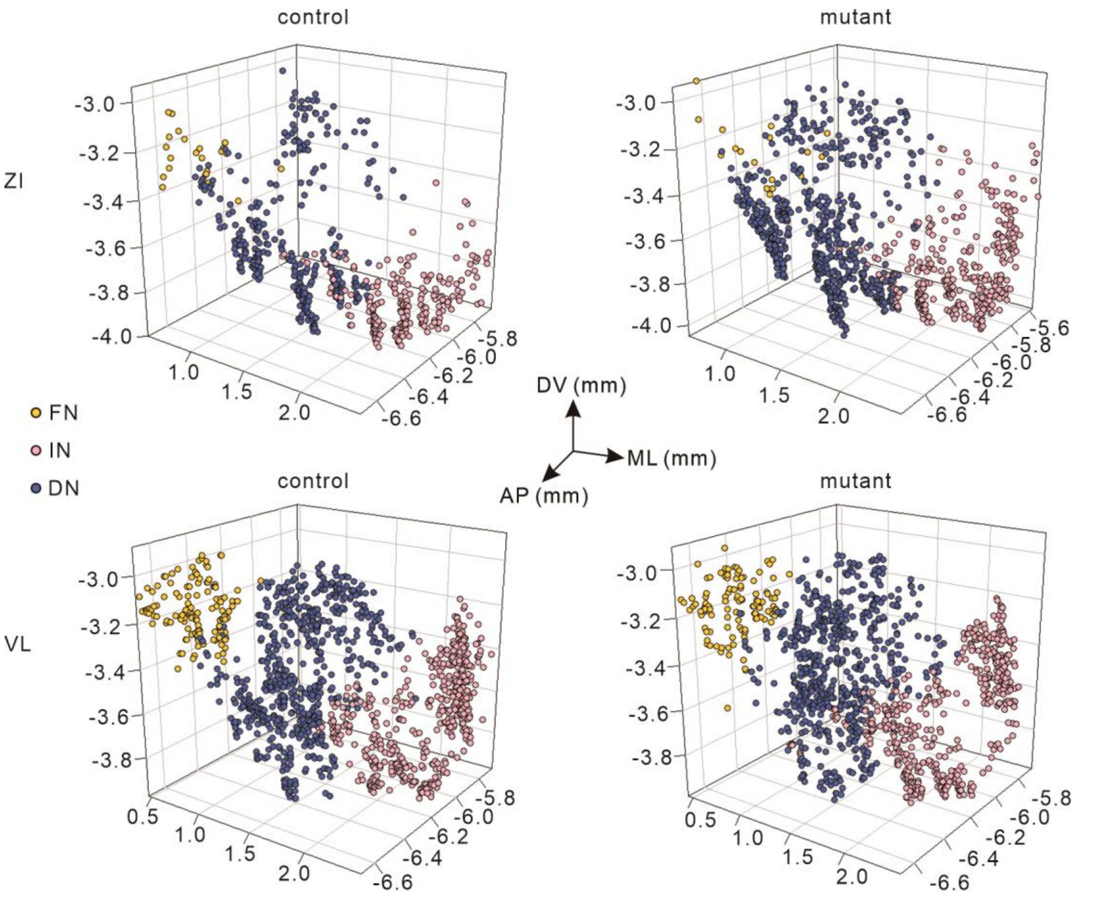


**Figure S25. 3D subdivisions of retrogradely labeled FN, IN and DN neurons**. Retrogradely labeled neurons were distinguished and reconstructed in 3D space. Left: 3D subdivision of FN, IN and DN neurons retrogradely traced from VL and ZI from a control mouse. This experiment was repeated in 6 control mice. Right: 3D subdivision of FN, IN and DN neurons retrogradely traced from VL and ZI from a mutant mouse. This experiment was repeated in 6 mutant mice.

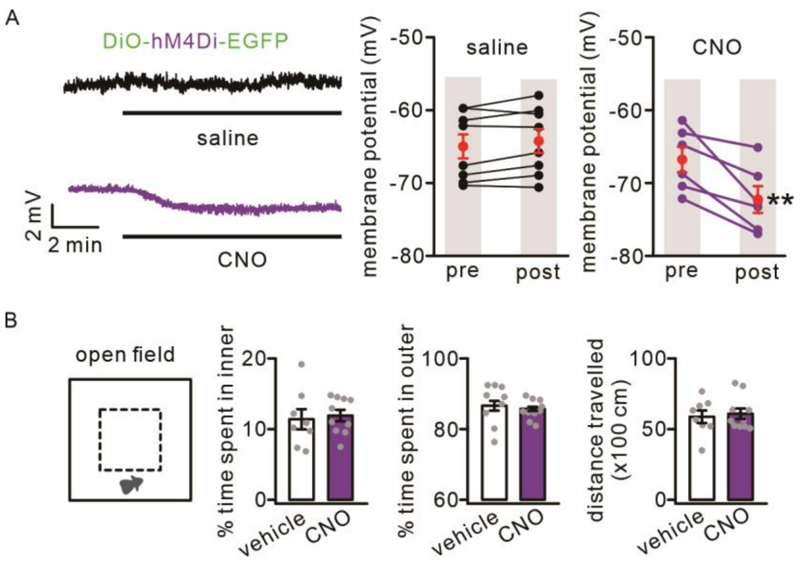


**Figure S26. Effects of chemoinhibition on membrane potential of $ZI^{IN}$ neurons and open field test.** (A) Current-clamp recording traces show that bath application of CNO (2 μM) induced a hyperpolarization of a neuron expressing hM4Di, whereas saline application had no effect. Bar graphs: changes in resting membrane potential of recorded neurons before and after the application of either saline or CNO. Saline: -65.0 ± 1.5 mV (pre) and -64.2 ± 1.5 mV (post), $n$ = 8, $F$ = 0.62, $t$ = 0.74, $P$ = 0.47. CNO: -66.8 ± 1.6 mV (pre) and -72.2 ± 1.7 mV (post), $n$ = 8, $F$ = 7.3, $t$ = 4.3, $P$ = 0.006. Paired $t$ test. **$P$ < 0.01. (B) In the open field test, mutant mice treated with vehicle or CNO showed no differences in time spent in the inner, outer zones, or move distance. For statistics, see Table S14.

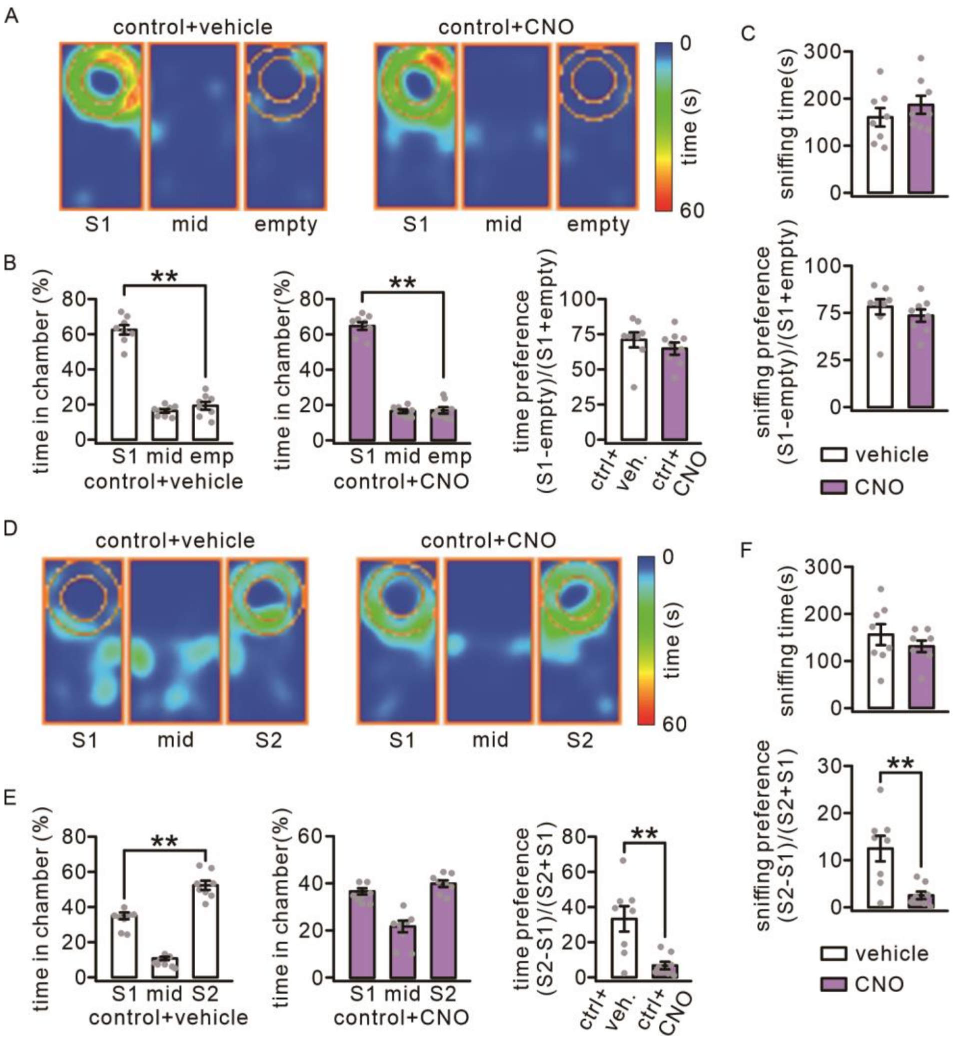


**Figure S27. Impaired aocial novelty in control mice treated with chemogenetic activation.** (A) Heat maps showing movement traces of mutant mice with chemogenetic activation. (B) The averages of spent time in S1, middle (mid), and empty (emp) chambers. (C) The averages of sniffing time and preference index with S1. (D) Heat maps showing movement traces of mutant mice with chemogenetic activation. (E) Mutant mice showed no interest to S2. (F) The averages of sniffing time and preference index with S2. For statistics, see Table S15. $^{**}P < 0.01$.

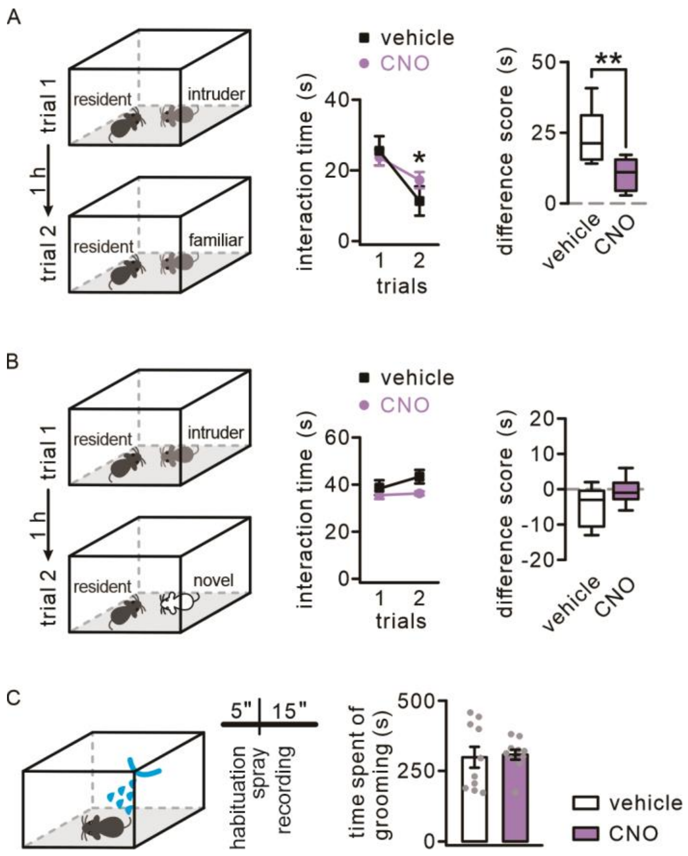


**Figure S28. Resident-intruder and grooming tests in control mice treated with chemogenetic activation.** (A) Intruder mouse was used in two consecutive trials with a familiar mouse. Control mice with CNO injection failed to display a decreased investigation time during trial 2, compared to vehicle injection mice. (B) Intruder mouse was used in two trials with a novel mouse. Vehicle and CNO groups explored two different mice similarly. (C) Cumulative time spent engaged in water spray-induced grooming behavior were scored over a 10-min session. For statistics, see Table S15. $^*P < 0.05$. $^{**}P < 0.01$.

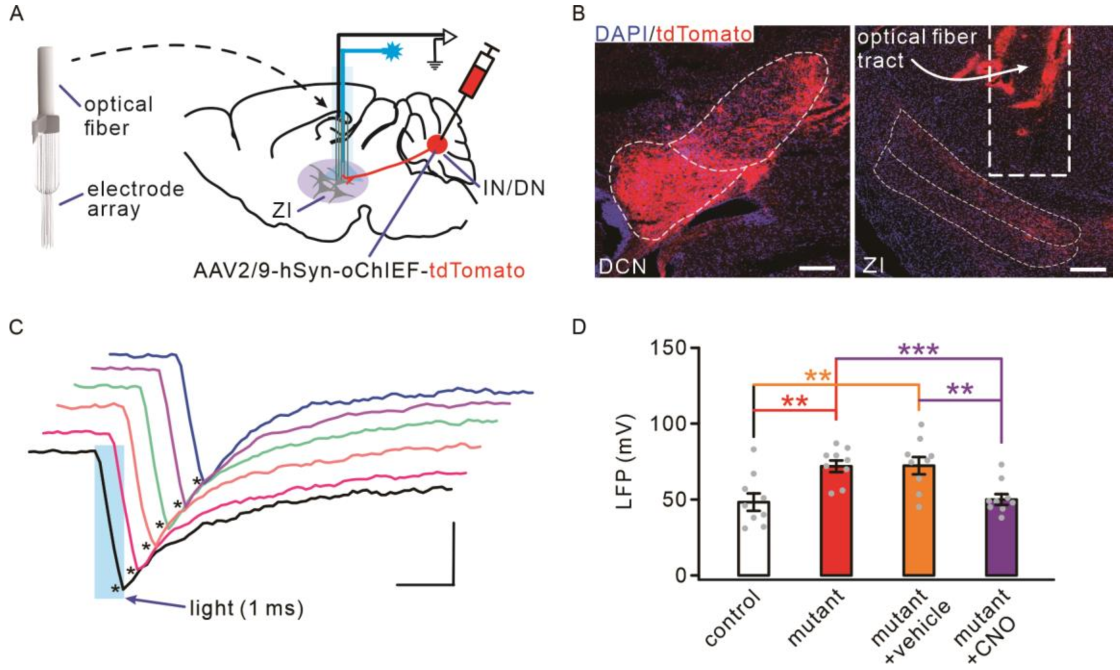


**Figure S29. LFPs evoked by light stimulation *in vivo.*** (A) Schematic of combined microelectrode recording and optogenetic control of axonal terminals of IN/DN→ZI pathway. (B) Viral expression in the IN/DN (left) and projected axon tracts in the ZI (right). An optic fiber was placed above the ZI to stimulate IN/DN axons. Scales: 200 μm. (C) LFPs recorded from 6 individual electrodes upon light stimuli at the terminals of IN/DN→ZI pathway. Scales: 25 mV/2 ms. The asterisks denotate the measurement of LFP peak. (D) Bargraphs show the averages of LFP amplitude. control: 49.4 ± 6.1 mV ($n$ = 9; $P$ = 0.0018 compared to mutant; $P$ = 0.0045 compared to mutant+vehicle; $P$ = 0.40 compared to mutant+CNO). mutant: 73.0 ± 4.1 mV ($n$ = 9; $P$ = 0.48 compared to mutant+vehicle; $P$ = 0.00038 compared to mutant+CNO). mutant+vehicle: 73.4 ± 6.0 mV ($n$ = 9; $P$ = 0.0022 compared to mutant+CNO). mutant+CNO: 51.2 ± 3.8 mV ($n$ = 9). One-way Anova test. **$P < 0.01$. ***$P < 0.001$.

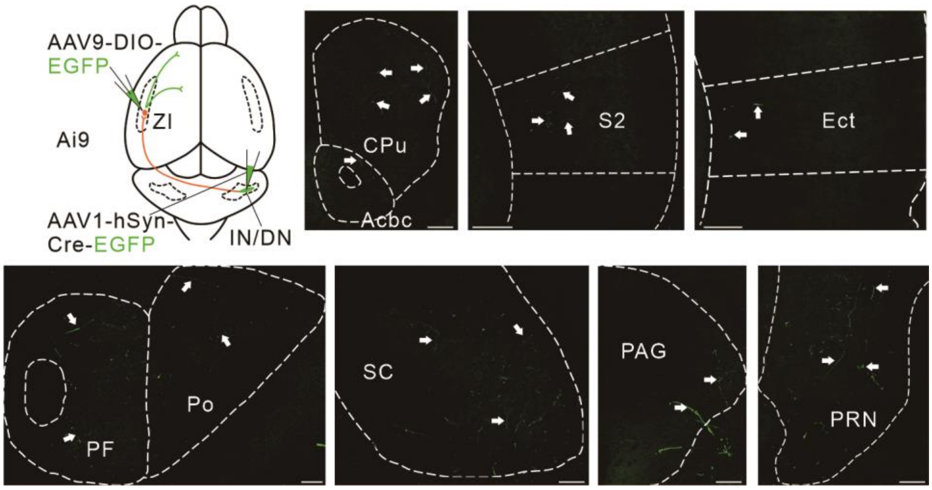


**Figure S30. Cortical and sub-cortical targets of $ZI^{IN}$ neurons.** Top left: stereotaxic injection scheme in Ai9 mice. Other panels: $ZI^{IN}$ neurons projected to several cortical and striatal areas, including Cpu, caudate putamen (striatum), S2, Ect, PF, Po, SC, PAG, and PRN. Scale bars: 200 μm.
