# Supplementary material for: Aberrant outputs of cerebellar nuclei and targeted rescue of social deficits in an autism mouse model
Source: Protein Cell. 2024 Jul 27;15(12):872–88. doi: 10.1093/procel/pwae040 (PMC11637611; doi:10.1093/procel/pwae040)
Supplement: pwae040_suppl_Supplementary_Tables [file pwae040_suppl_supplementary_tables.pdf]

Table S1

Nucleus Abbreviations

Abbreviations	Full name
3N	oculomotor nucleus
APT	anterior pretectal nucleus
CL	centrolateral thalamic nucleus
Dk	nucleus of Darkschewitsch
DpMe	deep mesencephalic nucleus
DR	dorsal raphe nucleus
Eth	ethmoid thalamic nucleus
EW	Edinger-Westphal nucleus
F	nucleus of the fields of Forel
InC	interstitial nucleus of Cajal
LC	locus coeruleus
LD	laterodorsal thalamic nucleus
LDTg	laterodorsal tegmental nucleus
LG	lateral geniculate nucleus
LH	lateral hypothalamic area
m5	motor root of the trigeminal nerve
MA3	medial accessory oculomotor nucleus
MD	mediodorsal thalamic nucleus
Mo5	motor trigeminal nucleus
PAG	periaqueductal gray
PB	parabrachial nucleus

pc	posterior commissure
Pcom	nucleus of the posterior commissure
PF	parafascicular thalamic nucleus
PH	posterior hypothalamic area
Po	posterior thalamic nuclear group
Pr5	principal sensory trigeminal nucleus
PrC	precommissural nucleus
RI	rostral interstitial nucleus of medial longitudinal fasciculus
RN	red nucleus
RPF	retroparafascicular nucleus
SC	superior colliculus
SPF	subparafascicular thalamic nucleus
Su3	supraoculomotor periaqueductal gray
Su5	supratrigeminal nucleus
TN	trigeminal nucleus
VL	ventrolateral thalamic nucleus
VM	ventromedial thalamic nucleus
VP	ventral posterior thalamic nucleus
VTA	ventral tegmental area
ZI	zona incerta

Table S2

Figure 1C-L (*n* = 10/group)

target nuclei	FN (%)	IN (%)	DN (%)
Figure 1D; VL	27.0 ± 4.6	36.6 ± 8.0	75.6 ± 4.2
Figure 1D; VP	17.5 ± 7.5	14.8 ± 3.5	4.7 ± 0.5
Figure 1E; LD	2.5 ± 0.3	3.0 ± 0.3	12.2 ± 2.8
Figure 1E; Po	2.1 ± 0.7	12.9 ± 3.2	17.1 ± 2.5
Figure 1F; VM	16.5 ± 2.8	9.8 ± 1.3	8.1 ± 1.8
Figure 1F; CL	14.6 ±1.7	8.3 ± 1.2	13.4 ± 1.7
Figure 1F; MD	7.0 ± 2.1	1.6 ± 0.6	1.5 ± 0.6
Figure 1G; PF	4.4 ± 0.8	4.3 ± 1.2	6.7 ± 1.4
Figure 1G; SPF	1.1 ± 0.2	1.3 ± 0.6	1.5 ± 0.1
Figure 1H; ZI	3.9 ± 0.3	13.6 ± 4.8	12.2 ± 3.6
Figure 1H; F	0.8 ± 0.2	0.6 ± 0.1	5.0 ± 0.2
Figure 1H; APT	2.7 ± 0.4	9.0 ± 1.0	25.2 ± 4.8
Figure 1H; RPF	2.3 ± 0.4	2.1 ± 0.1	5.7 ± 0.9
Figure 1H; Eth	1.1 ± 0.3	1.3 ± 0.5	1.9 ± 0.1
Figure 1I; VTA	4.6 ± 1.1	1.3 ± 0.6	1.4 ± 0.1
Figure 1I; RN	6.6 ± 2.3	5.3 ± 0.7	7.1 ± 1.0
Figure 1J; PAG	30.3 ± 9.7	11.3 ± 4.8	12.4 ± 5.9
Figure 1J; Dk	5.8 ± 0.5	0.8 ± 0.09	1.1 ± 1.6
Figure 1J; SC	26.0 ± 11.9	59.7 ± 9.2	64.2 ± 13.8
Figure 1J; InC	4.7 ± 0.1	1.5 ± 0.2	4.4 ± 0.4

Figure 1J; EW	1.4 ± 0.3	0.5 ± 0.1	2.0 ± 2.1
Figure 1K; Mo5	5.0 ± 0.4	1.9 ± 0.2	4.5 ± 0.3
Figure 1K; Pr5	4.0 ± 0.02	4.2 ± 0.04	6.1 ± 0.1
Figure 1K; m5	7.4 ± 0.1	6.0 ± 0.5	14.1 ± 2.4
Figure 1K; Su5	7.3 ± 0.1	5.0 ± 0.1	6.3 ± 0.9
Figure 1L; PB	16.5 ± 2.0	8.6 ± 0.2	4.2 ± 0.7
Figure 1L; LC	1.4 ± 0.4	1.5 ± 0.1	1.8 ± 0.1
Figure 1L; LDTg	16.0 ± 2.6	0.6 ± 0.1	1.1 ± 0.1

Table S3

Figure 2C (*n* = 6/group)

%	control		mutant		*F*	*t*	p
	average	SEM	average	SEM			
VL	31.6	2.1	26.8	3.5	2.77	1.07	0.31
VM	13.4	1.3	12.4	1.7	0.89	0.43	0.68
CL	14.6	1.9	6.1	0.9	1.02	3.67	0.004
MD	6.9	0.4	4.1	0.8	0.52	2.87	0.017
PAG	30.8	2.5	28.2	2.5	0.064	0.66	0.53
PF	4.2	0.3	2.4	0.5	1.65	2.66	0.024
Po	1.7	0.3	1.3	0.1	12.79	1.19	0.28
VTA	4.2	0.7	3.0	0.1	9.98	1.74	0.14
ZI	3.9	0.4	7.0	1.0	9.71	-2.56	0.042
APT	2.6	0.2	2.0	0.3	1.86	1.61	0.14
SC	27.3	4.5	31.7	3.7	0.55	-0.70	0.500
PB	25.7	1.6	28.1	3.9	1.72	-0.52	0.61

Table S4

Figure 3C (*n* = 6/group)

%	control		mutant		*F*	*t*	*p*
	average	SEM	average	SEM			
VL	49.3	5.4	46.0	6.2	0.42	0.36	0.72
VM	11.8	0.7	9.0	1.8	6.68	1.34	0.23
CL	6.8	1.2	7.6	1.1	0.001	-0.43	0.67
MD	1.8	0.3	1.1	0.3	0.28	1.50	0.17
PAG	9.3	1.4	9.3	0.9	2.004	0.028	0.98
VTA	2.8	0.6	0.9	0.1	7.70	2.70	0.040
Po	11.2	1.5	8.5	1.0	0.000	1.39	0.20
PF	4.2	0.5	3.5	0.4	0.49	0.89	0.39
ZI	12.9	1.5	45.7	7.5	5.39	-3.92	0.010
APT	5.1	0.9	5.1	1.3	2.51	-0.055	0.96
SC	52.4	9.4	52.2	6.6	2.97	0.019	0.99
PB	8.2	1.0	3.1	0.3	3.26	4.48	0.001

Table S5

Figure 4C (*n* = 6/group)

%	control		mutant		*F*	*t*	*p*
	average	SEM	average	SEM			
VL	59.7	4.2	65.6	5.2	0.044	-0.80	0.44
VM	10.9	1.4	8.8	1.3	0.086	1.0030	0.34
CL	13.0	0.8	11.9	1.2	1.72	0.76	0.47
MD	1.5	0.2	1.3	0.2	0.88	0.67	0.52
PAG	13.5	1.4	10.7	1.7	1.062	1.14	0.28
VTA	1.0	0.1	1.1	0.2	11.39	-0.42	0.69
Po	12.9	0.8	7.2	0.9	0.40	4.29	0.0020
PF	6.6	1.2	7.9	1.4	0.019	-0.66	0.53
ZI	17.9	2.5	48.3	8.3	2.97	-3.21	0.0090
APT	16.1	3.1	17.3	3.3	0.081	-0.25	0.81
SC	51.9	8.9	61.7	7.9	0.077	-0.82	0.43
PB	7.7	1.1	3.5	0.4	1.33	3.34	0.007

Table S6

Figure 5A, contralateral FN outputs

target nuclei	control		mutant		*F*	*t*	p
	average	SEM	average	SEM			
VL	31.6	2.1	26.8	3.5	2.77	1.07	0.31
CL	14.6	1.9	6.1	0.9	1.02	3.67	0.0040
PF	4.2	0.7	3.0	0.1	9.98	1.74	0.14
MD	6.9	0.4	4.1	0.8	0.52	2.87	0.017
VP	10.5	2.9	7.5	1.9	5.39	0.77	0.46
VM	13.4	1.3	12.4	1.7	0.89	0.43	0.68
LD	2.3	0.2	1.8	0.1	0.56	2.21	0.052
Po	1.7	0.3	1.3	0.1	12.79	1.19	0.28
ZI	3.9	0.4	7.0	1.0	9.71	-2.56	0.042
SPF	1.0	0.3	0.6	0.1	4.83	1.28	0.23
RN	4.8	1.1	6.4	1.8	1.66	-0.68	0.51
F	0.7	0.1	1.0	0.3	3.02	-0.8	0.44
RI	0.8	0.2	0.9	0.2	0.041	-0.26	0.80
APT	2.6	0.2	2.0	0.3	1.86	1.61	0.14
PrC	0.4	0.1	0.4	0.1	0.11	0.45	0.66
RPF	1.8	0.3	1.6	0.4	0.029	0.45	0.66
PAG	30.8	2.5	28.3	2.5	0.064	0.66	0.53
Eth	1.0	0.1	0.8	0.2	2.098	0.48	0.64
LG	0.3	0.1	0.7	0.1	3.78	-2.45	0.034

Dk	2.4	0.4	2.1	0.4	0.0080	0.48	0.64
EW	1.1	0.1	1.1	0.1	0.55	-0.46	0.65
PC	1.7	0.2	1.4	0.3	1.27	0.80	0.45
VTA	4.2	0.3	2.4	0.5	1.65	2.66	0.024
InC	2.1	0.3	1.9	0.3	0.014	0.60	0.56
MA3	0.5	0.1	0.5	0.1	0.46	1.03	0.33
DpMe	32.7	4.2	28.5	7.3	2.018	0.45	0.66
SC	27.3	4.5	31.7	3.7	0.55	-0.70	0.50
Su3	10.3	2.0	11.1	2.8	0.15	-0.20	0.84
3N	0.5	0.1	0.6	0.1	0.086	-0.30	0.77
DR	4.3	0.2	4.0	0.3	5.66	0.94	0.38
TN	20.2	1.3	40.2	3.4	9.63	-5.02	0.0020
PB	25.7	1.6	28.1	3.9	1.72	-0.52	0.61
LC	2.4	0.2	4.0	0.5	1.75	-3.005	0.013
LDTg	14.1	1.3	16.0	1.8	1.04	-0.75	0.47
LH	1.4	0.2	1.5	0.1	1.04	-0.22	0.83
PH	4.5	1.0	4.4	0.8	1.52	0.09	0.93

Figure 5A, contralateral IN outputs

target nuclei	control		mutant		*F*	*t*	p
	average	SEM	average	SEM			
VL	49.3	5.4	46.0	6.2	0.42	0.36	0.72
CL	6.8	1.2	7.6	1.1	0.0010	-0.43	0.67
PF	4.2	0.5	3.5	0.4	0.49	0.89	0.39
MD	1.8	0.3	1.1	0.3	0.28	1.50	0.17
VP	18.0	1.3	22.1	2.9	3.33	-1.17	0.27
VM	11.8	0.7	9.0	1.8	6.68	1.34	0.23
LD	5.1	1.4	4.8	0.7	5.53	0.15	0.89
Po	11.2	1.5	8.5	1.0	0	1.39	0.20
ZI	12.9	1.5	45.7	7.5	5.39	-3.92	0.010
SPF	1.6	0.3	3.8	0.8	7.07	-2.46	0.049
RN	5.1	0.6	6.4	1.5	5.45	-0.74	0.49
F	1.4	0.3	2.7	0.7	5.32	-1.51	0.18
RI	1.7	0.1	3.7	1.2	16.13	-1.59	0.17
APT	5.1	0.9	5.1	1.3	2.51	-0.055	0.96
PrC	0.5	0.1	1.1	0.2	2.79	-2.16	0.056
RPF	0.9	0.2	1.4	0.4	8.99	-1.09	0.32
PAG	9.3	1.4	9.3	0.9	2.00	0.028	0.98
Eth	2.0	0.4	3.4	0.9	6.73	-1.28	0.24
LG	3.5	1.2	5.7	0.9	0.051	-1.37	0.20
Dk	1.2	0.2	2.2	0.7	17.37	-1.17	0.29
EW	0.3	0.0	0.4	0.0	0.043	-1.18	0.27

PC	1.0	0.2	3.6	1.0	4.67	-2.27	0.047
VTA	2.8	0.6	0.9	0.1	7.70	2.70	0.040
InC	1.6	0.6	2.0	0.6	0.20	-0.47	0.65
MA3	0.5	0.0	0.8	0.1	3.72	-1.88	0.090
DpMe	15.1	2.4	23.8	2.0	0.0040	-2.54	0.029
SC	52.4	9.4	52.2	6.6	2.97	0.019	0.99
Su3	1.1	0.2	2.3	0.8	10.73	-1.35	0.23
3N	0.5	0.1	0.5	0.2	1.24	0.0050	1.00
DR	0.7	0.0	0.6	0.1	2.28	1.33	0.21
TN	13.3	0.4	11.4	2.2	5.39	0.79	0.46
PB	8.2	1.0	3.1	0.3	3.26	4.48	0.0010
LC	0.8	0.3	1.2	0.6	1.81	-0.51	0.62
LDTg	0.8	0.1	1.4	0.3	1.88	-1.72	0.12
LH	0.7	0.1	0.4	0.1	0.0090	1.92	0.084
PH	1.1	0.2	4.2	0.9	13.96	-3.08	0.025

Figure 5A, contralateral DN outputs

target nuclei	control		mutant		*F*	*t*	p
	average	SEM	average	SEM			
VL	59.7	4.2	65.6	5.2	0.044	-0.80	0.44
CL	13.0	0.8	11.9	1.2	1.72	0.76	0.49
PF	6.6	1.2	7.9	1.4	0.019	-0.66	0.53
MD	1.5	0.2	1.3	0.2	0.88	0.67	0.52
VP	3.2	0.4	2.8	0.4	0.049	0.68	0.51
VM	10.9	1.4	8.8	1.3	0.086	1.00	0.34
LD	10.2	2.2	9.3	1.3	1.94	0.31	0.76
Po	12.9	0.8	7.2	0.9	0.40	4.29	0.0020
ZI	17.9	2.5	48.3	8.3	2.97	-3.21	0.0090
SPF	2.0	0.5	3.7	0.5	0.0040	-2.22	0.049
RN	14.2	3.0	10.6	0.9	8.96	1.07	0.32
F	2.8	0.7	3.8	0.7	0.058	-0.91	0.38
RI	3.0	0.7	4.2	0.4	0.58	-1.35	0.21
APT	16.1	3.1	17.3	3.3	0.081	-0.25	0.81
PrC	1.6	0.3	2.1	0.2	0.0020	-1.52	0.16
RPF	2.8	0.6	4.7	0.5	0.055	-2.27	0.046
PAG	13.5	1.4	10.7	1.7	1.06	1.14	0.28
Eth	1.6	0.1	4.2	0.4	6.43	-6.09	0.0010
LG	11.7	1.0	8.7	2.0	0.55	1.20	0.26
Dk	2.4	0.5	3.1	0.5	0.0010	-1.07	0.31
EW	1.4	0.2	1.1	0.1	0.032	0.93	0.37

PC	2.3	0.5	5.2	0.4	0.14	-4.47	0.0010
VTA	1.0	0.1	1.1	0.2	11.39	-0.42	0.69
InC	3.1	0.2	4.3	0.4	1.48	-2.22	0.051
MA3	2.5	0.4	2.6	0.5	0.021	-0.22	0.83
DpMe	28.7	6.1	30.0	3.3	5.14	-0.17	0.87
SC	67.0	5.4	73.4	4.0	0.58	-0.88	0.40
Su3	4.0	0.6	4.6	0.5	0.92	-0.69	0.50
3N	0.7	0.1	0.7	0.2	0.28	-0.038	0.97
DR	1.9	0.2	1.8	0.2	0.46	0.42	0.68
TN	11.1	2.4	22.5	6.8	1.78	-1.44	0.18
PB	7.7	1.1	3.5	0.4	1.33	3.34	0.0070
LC	0.7	0.1	0.7	0.2	0.18	0.26	0.80
LDTg	1.1	0.1	1.2	0.2	3.24	-0.42	0.69
LH	0.6	0.2	0.7	0.2	0.037	-0.57	0.58
PH	4.9	0.8	7.8	1.3	0.50	-1.75	0.11

Figure 5A, ipsilateral FN outputs

target nuclei	control		mutant		*F*	*t*	*p*
	average	SEM	average	SEM			
VL	10.2	1.7	6.5	2.2	0.77	1.20	0.26
CL	4.5	0.7	3.0	0.7	0.000	1.39	0.120
PF	0.5	0.1	0.7	0.1	0.64	-2.36	0.040
MD	2.4	0.6	0.7	0.1	3.03	2.54	0.029
VP	0.5	0.2	0.3	0.0	9.35	1.20	0.28
VM	5.9	0.9	4.7	1.4	1.71	0.67	0.52
LD	0.4	0.1	0.6	0.0	0.98	-2.53	0.030
Po	0.5	0.2	0.3	0.1	2.12	0.88	0.40
ZI	0.5	0.1	0.6	0.1	4.92	-1.31	0.22
SPF	0.3	0.0	0.2	0.0	4.00	2.99	0.013
RN	0.7	0.1	0.5	0.1	0.012	1.25	0.24
F	0.2	0.0	0.2	0.0	0.20	0.40	0.70
RI	0.3	0.1	0.4	0.1	3.07	-0.80	0.44
APT	0.4	0.1	0.4	0.1	0.086	-0.37	0.72
PrC	0.0	0.0	0.0	0.0	-	-	-
RPF	0.0	0.0	0.0	0.0	-	-	-
PAG	10.5	2.0	8.2	1.2	1.82	0.91	0.38
Eth	0.1	0.0	0.2	0.0	0.31	-2.46	0.034
LG	0.0	0.0	0.0	0.0	-	-	-
Dk	0.5	0.1	0.5	0.1	1.40	0.052	0.96

EW	0.7	0.1	0.8	0.1	0.083	-1.10	0.30
PC	0.2	0.0	0.3	0.1	8.69	-1.37	0.22
VTA	0.8	0.1	0.4	0.1	1.60	2.32	0.042
InC	0.3	0.1	0.3	0.0	0.23	-0.070	0.95
MA3	0.3	0.1	0.3	0.1	0.14	0.44	0.67
DpMe	2.4	0.4	2.4	0.2	3.40	-0.032	0.98
SC	1.6	0.3	2.3	0.5	3.33	-1.16	0.27
Su3	0.8	0.2	0.9	0.2	0.45	-0.11	0.91
3N	0.3	0.0	0.3	0.0	2.09	-0.65	0.53
DR	2.2	0.1	1.9	0.2	3.90	1.05	0.32
TN	20.4	3.6	27.6	3.2	0.042	-1.35	0.21
PB	16.4	0.8	31.7	6.0	3.94	-2.32	0.043
LC	3.3	0.7	7.4	0.9	1.40	-3.28	0.0080
LDTg	4.3	0.7	6.5	1.2	0.36	-1.49	0.17
LH	0.6	0.1	0.4	0.1	0.25	2.04	0.069
PH	1.5	0.1	0.9	0.2	0.36	2.96	0.014

Figure 5A, ipsilateral IN outputs

target nuclei	control		mutant		*F*	*t*	*p*
	average	SEM	average	SEM			
VL	4.3	0.4	3.9	1.1	3.36	0.27	0.79
CL	5.3	0.9	3.5	0.3	1.70	1.83	0.097
PF	0.4	0.1	0.5	0.1	0.92	-1.23	0.25
MD	0.7	0.1	0.3	0.1	0.15	2.81	0.019
VP	0.4	0.1	0.2	0.0	2.56	4.24	0.0020
VM	1.5	0.3	0.8	0.2	0.073	1.80	0.79
LD	0.1	0.0	0.2	0.0	1.21	-2.24	0.049
Po	0.5	0.1	0.2	0.0	3.38	5.95	0.0010
ZI	0.5	0.1	0.6	0.2	0.50	-0.38	0.71
SPF	0.1	0.0	0.2	0.0	0.15	-2.34	0.041
RN	0.6	0.1	0.4	0.1	0.72	1.09	0.30
F	0.2	0.0	0.2	0.0	0.10	0.35	0.73
RI	0.2	0.0	0.2	0.0	0.42	0.12	0.90
APT	0.3	0.0	0.3	0.1	2.35	-0.099	0.92
PrC	0.2	0.0	0.1	0.0	0.0040	1.96	0.078
RPF	0.2	0.0	0.2	0.0	0.0010	1.23	0.25
PAG	2.3	0.6	2.3	0.4	0.70	0.000	1.00
Eth	0.1	0.0	0.1	0.0	0.0020	0.45	0.66
LG	0.0	0.0	0.0	0.0	-	-	-
Dk	0.8	0.2	0.3	0.1	9.22	1.53	0.17
EW	0.3	0.0	0.2	0.1	<0.001	0.39	0.71

PC	0.4	0.1	0.3	0.1	0.40	1.64	0.13
VTA	0.2	0.0	0.2	0.0	2.01	1.68	0.12
InC	0.1	0.0	0.3	0.1	2.95	-2.43	0.035
MA3	0.2	0.0	0.2	0.0	0.012	1.01	0.34
DpMe	0.6	0.2	0.8	0.3	0.68	-0.61	0.55
SC	1.8	0.2	1.3	0.3	8.38	1.48	0.17
Su3	0.4	0.1	0.5	0.0	0.94	-1.17	0.27
3N	0.4	0.1	0.2	0.0	1.41	2.11	0.061
DR	0.5	0.0	0.4	0.0	0.029	1.30	0.30
TN	71.7	1.3	67.2	1.7	0.97	1.97	0.078
PB	11.3	2.4	8.9	2.3	0.11	0.66	0.52
LC	5.0	1.1	3.2	0.8	0.33	1.30	0.22
LDTg	2.4	0.7	2.1	0.5	0.45	0.26	0.80
LH	0.1	0.0	0.1	0.0	0.29	0.035	0.97
PH	0.3	0.0	0.7	0.1	6.99	-2.45	0.056

Figure 5A, ipsilateral DN outputs

target nuclei	control		mutant		*F*	*t*	*p*
	average	SEM	average	SEM			
VL	3.9	0.7	3.3	0.3	2.50	0.69	0.51
CL	5.5	0.9	6.2	1.2	0.62	-0.40	0.70
PF	0.8	0.1	0.6	0.1	1.43	1.22	0.25
MD	0.6	0.2	0.5	0.1	2.44	0.41	0.69
VP	0.3	0.1	0.3	0.1	0.030	-0.38	0.72
VM	0.7	0.1	0.7	0.1	0.025	0.15	0.88
LD	0.0	0.0	0.0	0.0	-	-	-
Po	0.4	0.1	0.4	0.1	0.58	-0.51	0.62
ZI	0.3	0.1	0.4	0.1	1.74	-0.14	0.89
SPF	0.2	0.0	0.2	0.0	0.86	0.71	0.49
RN	0.3	0.0	1.5	0.4	5.41	-2.58	0.049
F	0.0	0.0	0.0	0.0	-	-	-
RI	0.2	0.0	0.2	0.0	1.62	-0.41	0.69
APT	0.3	0.0	0.5	0.2	2.96	-1.08	0.30
PrC	0.2	0.0	0.2	0.0	0.037	-0.13	0.90
RPF	0.2	0.0	0.3	0.0	1.35	-1.32	0.22
PAG	1.4	0.3	2.6	0.1	10.081	-3.85	0.0090
Eth	0.1	0.0	0.2	0.0	1.99	-2.64	0.025
LG	0.0	0.0	0.0	0.0	-	-	-
Dk	0.2	0.0	0.3	0.0	0.015	-1.02	0.33
EW	0.9	0.1	0.7	0.1	0.032	0.93	0.37

PC	0.2	0.0	0.2	0.0	0.90	-0.25	0.81
VTA	0.2	0.0	0.4	0.1	2.85	-1.46	0.17
InC	0.2	0.0	0.3	0.1	1.11	-1.58	0.15
MA3	0.3	0.0	0.2	0.0	2.14	0.22	0.83
DpMe	1.7	0.1	2.4	0.3	2.79	-2.19	0.054
SC	1.4	0.2	2.2	0.2	0.24	-2.26	0.047
Su3	0.4	0.1	0.3	0.1	0.43	0.86	0.41
3N	0.2	0.0	0.2	0.0	1.23	0.67	0.52
DR	1.2	0.1	1.1	0.2	0.13	0.32	0.76
TN	70.4	7.8	57.6	4.1	2.15	1.32	0.22
PB	10.8	1.3	11.6	1.7	0.47	-0.33	0.75
LC	1.4	0.2	1.5	0.3	0.66	-0.36	0.72
LDTg	2.0	0.1	4.3	1.4	4.51	-1.56	0.15
LH	0.2	0.0	0.3	0.1	2.20	-1.62	0.14
PH	0.6	0.1	1.1	0.2	0.95	-2.46	0.034

Figure 5A', 5B'

target nuclei	contralateral			ipsilateral		
	FN	IN	DN	FN	IN	DN
VL	-0.2	-0.1	0.1	-0.4	-0.1	-0.2
CL	-0.6	0.1	-0.1	-0.3	-0.4	0.1
PF	-0.3	-0.2	0.2	0.4	0.4	-0.2
MD	-0.4	-0.4	-0.1	-0.7	-0.6	-0.1
VP	-0.3	0.2	-0.1	-0.5	-0.6	0.1
VM	-0.1	-0.2	-0.2	-0.2	-0.5	0.0
LD	-0.2	-0.1	-0.1	0.5	0.4	0.0
Po	-0.2	-0.2	-0.5	-0.4	-0.7	0.1
ZI	0.8	2.5	1.7	0.3	0.2	0.0
SPF	-0.5	1.4	0.9	-0.5	0.4	-0.2
RN	0.3	0.3	-0.3	-0.2	-0.3	3.9
F	0.3	0.9	0.4	-0.1	-0.1	0.0
RI	0.1	1.2	0.4	0.3	0.0	0.1
APT	-0.2	0.0	0.1	0.1	0.0	0.6
PrC	-0.1	1.4	0.3	0.0	-0.3	0.0
RPF	-0.1	0.6	0.7	0.0	-0.2	0.3
PAG	-0.1	0.0	-0.2	-0.2	0.0	0.8
Eth	-0.1	0.7	1.7	0.5	-0.1	0.8
LG	1.2	0.7	-0.3	0.0	0.0	0.0
Dk	-0.1	0.8	0.3	0.0	-0.6	0.3

EW	0.1	0.2	-0.3	0.1	-0.1	-0.2
PC	-0.2	2.7	1.2	0.5	-0.4	0.1
VTA	-0.4	-0.7	0.1	-0.5	-0.2	0.5
InC	-0.1	0.3	0.4	0.0	1.2	0.6
MA3	-0.2	0.6	0.1	-0.1	-0.2	0.0
DpMe	-0.1	0.6	0.0	0.0	0.3	0.4
SC	0.2	0.0	0.1	0.5	-0.3	0.5
Su3	0.1	1.2	0.2	0.0	0.2	-0.2
3N	0.1	0.0	0.0	0.1	-0.4	-0.2
DR	-0.1	-0.2	-0.1	-0.1	-0.1	-0.1
TN	1.0	-0.2	1.0	0.4	-0.1	-0.2
PB	0.1	-0.6	-0.6	0.9	-0.2	0.1
LC	0.7	0.4	-0.1	1.2	-0.4	0.1
LDTg	0.1	0.7	0.1	0.5	-0.1	1.2
LH	0.0	-0.4	0.3	-0.4	0.0	0.8
PH	0.0	3.1	0.6	-0.4	1.1	0.8

Table S7

Figure 6C

%		other GABA	Glu	PV	nNOS
**control (*n* = 6)**	**average**	10.6	5.1	3.2	5.7
	**SEM**	1.0	0.5	0.4	0.6
**mutant (*n* = 6)**	**average**	16.6	7.5	4.5	13.7
	**SEM**	2.0	0.5	0.3	1.0
F		3.85	0.49	0.43	1.79
t		-2.43	-3.047	-2.13	-6.098
**p**		0.036	0.012	0.059	0.0010

Figure 6D

%		other types	nNOS	PV	other GABA	Glu
**control (*n* = 6)**	**average**	15.6	27.4	20.6	3.9	32.5
	**SEM**	1.4	2.0	1.4	0.6	2.2
**mutant (*n* = 6)**	**average**	3.6	25.1	24.8	3.8	42.7
	**SEM**	0.5	1.6	2.9	0.9	3.2
F		3.92	0.12	1.28	1.23	1.83
t		8.21	0.13	-0.60	0.22	-2.72
**p**		0.001	0.90	0.56	0.83	0.022

Table S8

Figure 7C

		S1 (%)	middle (%)	object (%)	time preference (%)	sniffing time in S1 (s)	sniffing preference (%)
vehicle (*n* = 8)	average	59.5	14.2	26.3	38.7	171.3	65.4
	SEM	3.1	1.6	2.8	6.3	29.0	7.0
CNO (*n* = 10)	average	58.5	16.3	25.2	38.3	175.4	65.6
	SEM	1.7	1.9	1.8	2.5	16.3	4.1
F		2.10	0.14	1.24	4.64	8.62	1.057
t		0.29	-0.79	0.32	0.061	-0.12	-0.023
p		0.78	0.44	0.75	0.95	0.91	0.982

Figure 7D

		S1 (%)	middle (%)	S2 (%)	preference index (%)	sniffing time in S2 (s)	sniffing preference (%)
vehicle (*n* = 8)	average	43.1	17.7	39.3	-5.2	77.6	6.5
	SEM	2.2	2.6	3.1	4.2	16.3	7.4
CNO (*n* = 10)	average	33.3	20.3	46.4	9.6	128.9	44.1
	SEM	2.1	2.9	2.3	2.3	14.3	4.8
F		0.19	0.81	1.18	2.51	0.019	1.742
t		3.042	-0.62	-1.78	-3.078	-2.23	-4.142
p		0.0080	0.55	0.094	0.0070	0.040	0.001

Table S9

Figure S4C

		S1 (%)	middle (%)	object (%)	time preference (%)	S1 sniffing time (s)	sniffing preference
**control (*n* = 8)**	**average**	58.3	16.3	25.4	38.9	217.0	46.3
	**SEM**	3.1	1.7	1.9	6.0	17.3	5.5
**mutant (*n* = 8)**	**average**	55.3	19.5	25.3	36.6	173.8	44.3
	**SEM**	3.6	2.5	2.3	6.4	21.7	7.5
F		0.78	0.70	0.21	0.66	0.30	0.657
t		0.60	-0.98	0.034	0.26	1.45	0.208
**p**		0.56	0.34	0.97	0.80	0.17	0.838

Figure S4F

		S1 (%)	middle (%)	S2 (%)	time preference (%)	S2 sniffing time (s)	sniffing preference
**control (*n* = 8)**	**average**	29.7	24.8	45.5	21.3	130.6	20.8
	**SEM**	2.4	2.0	2.2	5.6	4.8	3.0
**mutant (*n* = 8)**	**average**	39.2	20.5	40.3	1.3	97.4	-7.3
	**SEM**	1.4	2.3	1.7	2.6	4.0	7.9
F		1.86	0.28	0.38	6.37	0.070	5.032
t		-3.13	1.30	1.79	3.036	4.99	3.126
**p**		0.0070	0.22	0.096	0.013	0.0010	0.012

Figure S4G

	control (*n* = 15)		mutant (*n* = 20)		*F*	*t*	*p*
	average	SEM	average	SEM			
time spent of grooming (s)	265.9	16.0	279.0	21.0	2.88	-0.46	0.65

Table S10

Figure S5A, S5B

		Figure S5A			Figure S5B		
		familiar trial-1	familiar trial-2	familiar index	novel trial-1	novel trial-2	novel index
control (*n* = 7)	average	24.0	12.6	12.8	26.1	24.5	1.7
	SEM	3.3	3.2	2.7	2.8	2.7	1.0
mutant (*n* = 5)	average	18.7	18.0	0.8	21.6	22.1	-0.5
	SEM	2.3	2.3	2.1	1.6	2.6	1.5
F		1.99	0.53	1.04	0.79	0.092	0.22
t		1.20	-1.27	3.24	1.25	0.60	1.29
p		0.26	0.23	0.0090	0.24	0.56	0.23

Figure S5C, S5D

		Figure S5C			Figure S5D		
		familiar trial-1	familiar trial-2	familiar index	novel trial-1	novel trial-2	novel index
control (*n* = 7)	average	23.2	22.2	1.0	25.4	24.5	0.9
	SEM	2.5	1.1	2.9	5.5	3.2	5.0
mutant (*n* = 5)	average	25.2	16.2	9.0	24.9	25.3	-0.4
	SEM	3.0	2.1	4.0	5.7	3.0	7.2
F		1.50	14.51	0.66	0.26	0.14	2.35
t		-0.49	2.52	-1.50	0.064	-0.17	0.13
p		0.64	0.033	0.16	0.95	0.87	0.90

Table S11

number / 0.1 mm^2		Figure S6A			Figure S6B (vGluT2)			Figure S6C (GABA)		
		FN	IN	DN	FN	IN	DN	FN	IN	DN
control (n = 5)	average	101.0	118.5	126.0	44.2	55.3	65.3	43.4	54.2	52.9
	SEM	3.2	3.9	4.0	1.9	3.5	1.6	2.7	3.9	3.7
mutant (n = 5)	average	105.0	113.0	117.0	43.2	54.7	63.7	42.0	51.5	50.2
	SEM	4.30	2.68	4.97	1.91	2.16	2.62	3.95	3.62	2.92
F		1.28	0.73	0.18	0.031	3.29	0.82	1.02	0.04	0.50
t		-0.67	1.05	1.26	0.35	0.12	0.46	0.28	0.51	0.58
**p**		0.52	0.33	0.24	0.74	0.91	0.66	0.78	0.62	0.58

Figure S7C, S7D

	control		mutant		***F***	***t***	**p**
	average	SEM	average	SEM			
PC	14.4	0.7	14.4	1.1	1.47	0	1.00
IN	12.0	0.4	12.8	0.7	2.67	-0.93	0.38

Figure S7E

%	control		mutant		***F***	***t***	**p**
	average	SEM	average	SEM			

**GluA1**	100.0	10.9	102.4	12.6	0.56	-0.14	0.89
**GluA2**	100.0	5.1	100.8	9.0	6.24	-0.078	0.94
**GluN1**	100.0	6.4	93.3	7.6	0.15	0.67	0.53
**GluN2A**	100.0	14.6	81.8	6.8	1.48	1.13	0.30
**GluN2B**	100.0	2.4	96.2	4.8	5.67	0.72	0.50
**mGlu1**	100.0	5.3	97.6	10.2	7.08	0.21	0.84
**mGlu5**	100.0	6.4	92.1	2.8	1.58	1.14	0.30
**PSD95**	100.0	6.4	101.0	3.4	0.90	-0.14	0.89

Table S12

Figure S10C, S10H

%	control (n = 5)		mutant (n = 5)		F	t	p
	average	SEM	average	SEM			
VL_v^{FN}	14.6	0.9	21.5	2.3	7.094	-2.536	0.050
VL_d^{DN}	34.9	4.4	56.8	5.5	0.032	-2.807	0.023

Figure S11E, S10H

%	control (n = 5)		mutant (n = 5)		F	t	p
	average	SEM	average	SEM			
ZI_d^{IN}	12.7	2.6	27.2	4.6	2.81	-2.45	0.040
ZI_d^{DN}	24.1	5.1	46.6	4.0	1.38	-3.00	0.024

Figure S12C

%	control (n = 6)		mutant (n = 6)		F	t	p
	average	SEM	average	SEM			
MDm^{FN}	2.0	0.3	1.0	0.1	6.84	2.49	0.044

Figure S13C

%	control (n = 6)		mutant (n = 6)		F	t	p
	average	SEM	average	SEM			

CLc^{FN}	4.1	0.8	1.1	0.3	9.75	3.19	0.025

Table S13

Figure S20B’, S20C’

%		Figure S20C’			Figure S20B’		
		FN^{VL}	IN^{VL}	DN^{VL}	FN^{ZI}	IN^{ZI}	DN^{ZI}
**control (*n* = 5)**	**average**	7.96	41.06	35.06	0.48	11.50	7.30
	**SEM**	1.00	2.09	0.48	0.13	0.69	1.00
**mutant (*n* = 5)**	**average**	9.78	38.96	32.04	0.46	15.68	10.42
	**SEM**	1.88	3.98	3.36	0.070	1.07	0.43
F		5.28	5.11	3.15	2.18	0.63	7.07
t		-0.77	0.42	0.80	0.12	-2.93	-2.57
**p**		0.47	0.69	0.45	0.91	0.019	0.046

Figure S20D, S20E

%		vGluT2			GABA		
		**FN**	**IN**	**DN**	**FN**	**IN**	**DN**
**control (*n* = 5)**	**average**	0.33	8.07	4.84	0.19	3.85	2.52
	**SEM**	0.09	0.48	0.66	0.04	0.12	0.28
**mutant (*n* = 5)**	**average**	0.36	11.87	7.91	0.22	5.65	3.84
	**SEM**	0.05	1.18	0.53	0.04	0.59	0.31
F		1.35	1.82	1.06	0.32	7.40	0.20
t		-0.30	-2.67	-3.25	-0.40	-2.99	-3.19
**p**		0.78	0.028	0.012	0.71	0.04	0.01

Table S14

Figure S21C

%		FN	IN	DN
**control (*n* = 5)**	**average**	1.09	17.59	10.22
	**SEM**	0.13	1.08	1.20
**mutant (*n* = 5)**	**average**	1.53	29.11	20.42
	**SEM**	0.13	2.35	1.86
F		0.13	3.30	1.28
t		-2.19	-3.98	-4.12
**p**		0.060	0.0040	0.0030

Figure S26B

		time spent in inner (%)	time spent in outer (%)	distance travelled (x100 cm)
**vehicle (*n* = 8)**	**average**	86.9	11.6	59.5
	**SEM**	1.9	1.3	4.2
**CNO (*n* = 10)**	**average**	86.0	12.1	61.7
	**SEM**	0.8	0.8	3.5
F		4.64	0.80	0.012
t		0.41	-0.35	-0.37
**p**		0.69	0.74	0.72

Table S15

Figure S27B, S27C

		Figure S27B				Figure S27C	
		S1(%)	middle	empty (%)	time preference	S1 sniffing time (s)	sniffing preference
vehicle (n = 8)	average	63.1	16.9	19.9	71.8	81.13	71.8
	SEM	2.5	1.0	2.1	5.1	9.13	5.1
CNO (n = 8)	average	65.3	17.1	17.6	65.5	94.38	65.5
	SEM	2.0	1.0	1.7	4.1	8.96	4.1
F		0.236	0.528	0.445	0.015	0.007	0.023
t		-0.687	-0.161	0.916	0.881	-0.969	0.899
p		0.503	0.875	0.375	0.393	0.349	0.384

Figure S27E, S27F

		Figure S27E				Figure S27F	
		S1 (%)	middle (%)	S2 (%)	time preference	S2 sniffing time (s)	sniffing preference
vehicle (n = 8)	average	35.6	11.4	53.0	42.4	31.63	42.4
	SEM	1.8	0.9	2.5	8.4	4.17	8.4
CNO (n = 8)	average	37.2	22.3	40.5	9.2	26.63	9.2
	SEM	1.2	2.3	1.3	2.5	2.30	2.5
F		0.66	4.95	2.61	8.13	3.87	8.007

t	-0.68	-4.04	4.12	3.54	0.98	0.013
**p**	0.51	0.0030	0.0010	0.0070	0.34	0.007

Figure S28A, S28B

		**Figure S28A**			**Figure S28B**		
		**familiar trial-1**	**familiar trial-2**	**familiar index**	**novel trial-1**	**novel trial-2**	**novel index**
**vehicle (*n* = 5)**	**average**	25.6	10.2	22.0	40.0	42.8	-2.8
	**SEM**	3.9	2.0	4.0	3.3	2.9	1.6
**CNO (*n* = 6)**	**average**	22.5	17.4	10.5	36.6	38.1	-1.5
	**SEM**	1.9	1.5	1.6	1.5	1.5	1.0
F		1.64	0.001	1.10	0.031	18.84	0.42
t		0.80	-2.88	3.12	-0.66	3.86	-0.68
**p**		0.443	0.015	0.01	0.52	0.033	0.51

Figure S28C

**s**	**vehicle (*n* = 10)**		**CNO (*n* = 10)**		***F***	***t***	**p**
	**average**	**SEM**	**average**	**SEM**			
**time spent of grooming**	291.3	31.2	301.0	18.4	7.47	-0.27	0.79
