# Supplementary material for: Aberrant outputs of cerebellar nuclei and targeted rescue of social deficits in an autism mouse model
Source: Protein Cell. 2024 Jul 27;15(12):872–88. doi: 10.1093/procel/pwae040 (PMC11637611; doi:10.1093/procel/pwae040)
Supplement: pwae040_suppl_Supplementary_File [file pwae040_suppl_supplementary_file.pdf]

Methods

Animals

All experiments were carried out in a strict compliance with protocols approved by the Animal Care and Use Committee at Zhejiang University (ZJU) School of Medicine. Mice were kept under temperature-controlled conditions on a 12:12 h light/dark cycle with food and water ad libitum at the animal facility at ZJU. Original Nlgn3R451C mice (no. 008475) and Ai9 mice (no. 007909) were obtained from the Jackson Laboratory (Bar Harbor, ME). The resulting offspring were genotyped using PCR of genomic DNA (*Nlgn3*^{R451C}: WF, 5'-CTG GGC TGG ATG TAA GGC GAG G -3'; WR, 5'- CAC CAT TTT ATG TTT TCT GCA AGG GC -3'; MR, 5'- GTT CCA TGT CAC TAC ATG CTC TGA CCC -3'; Ai9: WF, 5'-AAG GGA GCT GCA GTG GAG TA-3'; WR, 5'-CCG AAA ATC TGT GGG AAG TC-3'; MF, 5'-GGC ATT AAA GCA GCG TAT CC-3'; MR: CTG TTC CTG TAC GGC ATG G-3'). Both Nlgn3R451C mice and Ai9 mice were in the C57BL/6J genetic background, as they were backcrossed to C57BL/6J mice for 10 generations. All experiments were performed in age-matched male mice.

Antibodies

Anti-vGluT2 antibody was a gift from Masahiko Watanabe (Hokkaido University, Sapporo, Japan). Antibodies against GABA (A2052; RRID: AB_477652) and glutamate (G6642; RRID: AB_259946) were from Sigma (St. Louis, MO). The antibody against parvalbumin (pv27; RRID: AB_2631173) was from Swant (Burgdorf, Switzerland). The antibody against nNOS (61-7000; RRID: AB_2313734) was from Thermo-Fisher (Waltham, MA). The antibody against SMI32 (SMI-32R-100; RRID: AB_509997) was from Covance (Princeton, CT). Antibodies against GluA1 (04-855, RRID: AB_10015249), GluA2 (MAB397, RRID: AB_2113875), GluN1 (17-312, RRID: AB_390251) and GluN2A (06-313, RRID: AB_2314980) were from Millipore (Billerica, MA). Antibody against GluN2B (14544, RRID: AB_2798506) were from CST (Danvers, MA). Anti-mGlu1 antibody was from BD Biosciences (San Jose, CA). Anti-mGlu5 (nb100-79995, RRID: AB_1109321) was from Novus Biologicals (Littleton, CO). Anti- PSD95 (ab2723, RRID: AB_303248) was from Abcam (Cambridge, UK). Anti-β-actin (sc-47778, RRID: AB_626632) was from Santa Cruz (Santa Cruz, CA). 4',6-diamidino-2-phenylindole (DAPI) and Alexa Fluor-conjugated secondary antibodies were from Invitrogen (Carlsbad, CA).

Recombinant virus and in vivo injection

AAV1-hSyn-Cre-EGFP (6.68E13 FFU/ml) and AAV9-hSyn-DIO-EGFP (2.3E12 FFU/ml) virus were from Sunbio Medical Biotech (Shanghai, China). AAV2-hSyn-EGFP-IRES-WGA-Cre (1.67E13 FFU/ml), AAV9-hSyn-DIO-hM4Di(Gi)-EGFP (1.96E13 FFU/ml) and AAV9-hSyn-DIO-hM3Dq(Gq)-EGFP (0.79E13 FFU/ml) virus were from Taitool Bioscience (Shanghai, China). RV-ENVA-ΔG-EGFP (2E8 FFU/ml), rAAV-hSyn-mCherry-F2A-TVA-WPRE-hGH-pA (5E12 FFU/ml) and rAAV-hSyn-oRVG-WPRE-hGH-pA (5E12 FFU/ml) virus were from BrainVTA (Hubei, China). The viruses used in the present work did not discriminate excitatory or inhibitory neurons.

Mice were anesthetized with sodium pentobarbital (0.7%) via intraperitoneal injection

and viruses were injected into brain nuclei at a rate of 20 nl/min using a glass pipette and a stereotaxic equipment (World Precision Instruments, FL). For anterograde tracing, AAV1-hSyn-Cre-EGFP solution (100 nl) was unilaterally injected into the FN (ML: 0.74; AP: –6.30; DV: –3.25; from bregma), the IN (ML: 1.45; AP: –6.24; DV: –3.32; from bregma), and the DN (ML: 2.15; AP: –6.12; DV: –3.42; from bregma). For anterograde tracing from ZICN neurons, AAV9-hSyn-DIO-EGFP viral solution (80 nl) was injected into the ZI (ML: 1.60 mm; AP: –2.54 mm; DV: –3.98 mm; from bregma). For retrograde tracing, AAV9-hSyn-WGACre-EGFP solution (60 nl) was injected into the VL (ML: 1.10 mm; AP: –1.10 mm; DV: –3.50 mm; from bregma) or the ZI (ML: 1.60 mm; AP: –2.54 mm; DV: –3.98 mm; from bregma). Solution of RV-helpers was injected into the VL and ZI (200 nl), and then RV-ENVA-ΔG-EGFP was injected into VL and ZI after two weeks (300 nl). The coordinates of VL and ZI were the same as mentioned above. For designer receptors exclusively activated by designer drug (DREADD) experiment, AAV1-hSyn-Cre-EGFP solution (100 nl) was bilaterally injected into the IN and DN (ML: ±1.45 mm; AP: –6.24 mm; DV: –3.32 mm; from bregma). After 4 weeks, AAV9-hSyn-DIO-hM4Di(Gi)-EGFP or AAV9-hSyn-DIO-hM3Dq(Gq)-EGFP solution (70 nl) was injected into the ZI (ML: ±1.60 mm; AP: –2.54 mm; DV: –3.98 mm; from bregma). For LFP recording, AAV2/9-hSyn-oChIEF-tdTomato-WPRE-pA (150 nl) was injected into the IN and DN (ML: -1.45 mm; AP: –6.24 mm; DV: –3.32 mm; from bregma). AAV1-hSyn-Cre-EGFP solution (100 nl) and AAV2/9-hSyn-oChIEF-tdTomato-WPRE-pA (150 nl) were injected into IN/DN, AAV9-hSyn-DIO-hM4Di(Gi)-EGFP (70 nl) was injected into the ZI three weeks later.

Immunohistochemistry

Mice were deeply anesthetized with sodium pentobarbital and perfused transcardially with phosphate buffer, followed by 4% paraformaldehyde in phosphate buffer. After the post-fixation overnight at 4°C, brains were dehydrated with 30% sucrose. Continuous frozen coronal sections (20 μm) from the forebrain to the cerebellum (bregma 3.08 to –5.50) were obtained using a cryostat microtome (Thermo Fisher Scientific, Waltham, MA) and placed sequentially in 96-well plates. For cell counting, brain slices were sequentially mounted and labeled on gelatin-coated slides and imaged using an Olympus SLIDEVIEW VS120 (Olympus, Tokyo, Japan). For antibody fluorescence, sequential layers of the VL, ZI and CN were transferred in blocking solution for 1 hr at room temperature (RT). After washing with PBS, sections were incubated with primary antibodies overnight at 4ºC. Primary antibody dilutions used for immunohistochemistry were 1:300 (GABA), 1:500 (vGluT2, parvalbumin, nNOS, and glutamate), and 1:1,000 (SMI32). After rinses with PBS, sections were incubated with secondary antibodies (1:1,000 for dilution) for 2 hrs at RT, washed with PBS, and stained with DAPI. Immunohistochemical images were obtained with an A1R confocal microscope (Nikon, Tokyo, Japan). The parameters used in microscopy were consistent in all experiments.

H&E staining

H&E staining was performed using Hematoxylin and Eosin staining kit. Coronal cerebellar slices (30 μm) were immersed in hematoxylin staining solution for 5-10 min, rinsed with distilled water, and immersed in eosin staining solution for 2 min. The sections were rinsed

with distilled water, dehydrated in ethanol, and cleared in xylene. Images of cerebellar cortex were captured using a BX53 microscope (Olympus, Tokyo, Japan). Cell density was calculated using Fiji for quantification.

Golgi staining and Sholl analysis

Golgi staining was performed using Rapid Golgi Stain Kit according to the standard procedure. PCs at the apical region were imaged using a bright field microscope (Olympus BX61). NeuroLucida software was used to plot soma and dendrites under a manually assisted mode. Sholl analysis was adopted according to previous work56. A series of concentric circles (10 μm interval) were plotted around the soma. The number of intersections of dendrites for each circle and the dendrite length within each 10 μm segment were quantified using NeuroLucida software.

Western blotting

Cerebellar nuclei sections were washed with PBS and lysed with 1% SDS containing protease inhibitors. After protein concentration was determined by BCA protein assay, equal amounts of each group of proteins were separated on SDS-PAGE and transferred to PVDF membranes, incubated with antibodies, and visualized using enhanced chemiluminescence for visualization. Primary antibodies were diluted as follows GluA1 (1:2,000), GluA2 (1:2,000), GluN1 (1:2,000), GluN2A (1:2,000), GluN2B (1:1,000), mGluR1 (1:1,000), mGluR5 (1:1,000), PSD95 (1:5,000), β-actin (1:1,000), and GAPDH (1:5,000) and secondary antibodies (1:10,000). The film signals were quantified and analyzed using Fiji.

Anatomic analysis

To calculate injection coverage, we acquired binary values for each image using Threshold function of Fiji and a baseline value was set based on 5 background pixels. The area with the intensity of at least 1.5-fold of the background value was determined as the actual injection fraction. For counting neurons, all slices were registered with the Allen Mouse Common Coordinate Framework (CCF) to normalize brain slices across investigated mice. In the coronal planes, labeled neurons were defined by the co-localization of tracer signal and DAPI at a fixed signal-to-noise ratio and counted using Cell Counter and Analyze Particles functions of Fiji. To re-construct labeled neurons in 3D space, the coordinates of neurons were recorded after registering them using CCF, by which the animation showing 3D positions of neurons was constructed using Matlab or Rx64 4.0.0. To count neuronal numbers in the nuclei, the levels that contain target nuclei in anterior-posterior or mediolateral direction were selected and the representative shape of each nucleus was drawn in CorelDraw software according to the brain atlas. Subsequently, each nucleus was filled with a color representing the difference between mutant and control mice using Prism 8.0.

Quality control of neuronal counting

Two investigators independently counted neurons for each slice section. The results showed that there was no difference between two investigators for more than 90% of

counts. If there was a difference, we took the average of two counts as the final value. The investigators were blinded for the genotypes of counted mice. A confocal picture of one section and a vector graph of registered brain map were imported into a file of Coreldraw software. The picture and the map were overlapped to determine whether they belong to a same bregma level. Traced neurons were judged based on the staining color and shape. All these procedures were conducted using a large screen computer monitor for the high resolution. We recruited all sections (from the anterior to the posterior) of a nucleus according to the brain map. Thus, all neurons within a nucleus range were counted. All cells within the range of an individual picture were inspected.

Electrophysiology

All chemicals used in electrophysiology were from Sigma (St. Louis, MO). Coronal slices of the ZI (250 μm) were prepared from anesthetic mice using a vibrating tissue slicer (VT1000S, Leica, Germany) and ice-cold standard artificial cerebrospinal fluid (aCSF) containing (in mM): 125 NaCl, 2.5 KCl, 1.25 NaH2PO4, 1 MgCl2, 2 CaCl2, 26 NaHCO3 and 25 D-glucose, bubbled with 95% O2/5% CO2. After recovery for 30 min at 37°C, slices were placed in a submerged chamber that was perfused at 2 ml/min with aCSF. Patch clamp electrodes (3-5 MΩ) were filled with an intracellular solution composed of (in mM) 134 K-gluconate, 6 KCl, 4 NaCl, 10 HEPES, 0.2 EGTA, 4 Na2ATP, 0.3 Na3GTP, and 14 Na2phosphocreatine (pH 7.3, OSM 290) for current-clamp recordings. Neurons were visualized under an upright microscope (BX51, Olympus) with a 40× water-immersion objective and equipped with infrared differential interference contrast enhancement. Whole-cell recordings were obtained with an Axon MultiClamp 700B amplifier (Molecular Devices, CA). Currents were digitized at 10 kHz and filtered at 3 kHz. Offline analysis was conducted using a sliding template algorithm (ClampFit 10, Molecular Devices, CA)57.

LFP recording

LFP recordings were performed according to our previous work (Ma et al., 2021). 3 weeks after injection, mice were anesthetized with urethane (1.25 g/kg, i.p.) and placed in a stereotaxic apparatus (69100, RWD, Shenzhen, China). The scalp was incised and part of the skull was removed to allow for the implantation of the electrodes. For stimulation and LFP recording, a microelectrode (2 × 4) array combined with an optical fiber (KD-MEA-F, KedouBC, Suzhou, China) were placed above the ZI (ML:1.60 mm; AP: –2.54 mm; DV: –3.98 mm; from bregma). With light stimulation at 450 nm, brain signals were sampled by an amplifier (Model 3600, A-M Systems, Sequim, WA) and a PowerLab ML880 16/35 acquisition system (AD Instruments, Colorado Springs, CO). The sampling rate was 20 kHz.

Open field test

Before experiments, mice were handled daily for 3 days and 75% ethanol was used to clean mouse chambers. For chemogenetic stimulation, animals were intraperitoneally administrated with CNO (1 mg/kg) or control vehicle 25-30 min prior to test. Mice were placed in a novel brightly-lit square (46 cm × 46cm × 28 cm, length × width × height) plexiglass chamber for 15 min. The activity of mice was recorded and analyzed by

ANY-maze. During analysis, the arena was subdivided into two concentric zones named inner (23 cm × 23 cm, length × width) and outer zones, respectively. Time and travel distance of animals in each zone were recorded.

Grooming test

Mice were acclimated in an empty clean cage (30 cm × 20 cm × 20 cm, length × width × height) in the bright condition for 5 min and then given a single puff of water spray to induce grooming behavior (Peter et al., 2016; Xu et al., 2023). Mouse activity after receiving water spray was recorded for 15 min using a high-definition camera.

Three chamber test

Three-chamber social test was conducted in mice as described previously (Zhou et al., 2017; Xu et al., 2023). The apparatus consisted of a rectangular plexiglass box (60 cm × 35 cm × 10 cm, length × width × height) evenly divided into three chambers. The test mouse was placed in the central chamber for 10-min habituation. S1 mouse was introduced into a wire cage in one chamber and empty chamber served as an inanimate object with no social valence. The test mouse was allowed to freely explore all three chambers over a 10-min session. Next, S2 mouse was introduced in the other chamber, replacing the inanimate object. As a second examination, the test mouse was again allowed to spend 10 min to explore all three chambers. The time spent in each chamber was recorded, and for the first examination the ratio of (S1-E) to (S1+E) was calculated as the preference index (S1-E) and for the second examination the ratio of (S2-S1) to (S2+S1) was measured as the preference index (S2-S1). Heat maps were automatically generated using ANY-maze.

Resident-intruder test

Male mice (4 weeks old) were used as stimulus mice. The test was performed in a home cage of a test mouse and began when a stimulus mouse was introduced to the cage. The test mouse was allowed to explore stimulus mouse for 5 min (trial 1), then the stimulus mouse was removed. After an inter-trial interval of 1 h, the test mouse was re-exposed original (the stimulus mouse in trial 1) or a novel stimulus mouse for another 5 min (trial 2). All social behaviours (body sniffing, anogenital and nose-to-nose sniffing, following, and allogrooming) initiated by test subject in the first 1 min in trial 1 and trial 2 was measured. The difference score was calculated by subtracting the time spent in social interaction during trial 2 from the social interaction time during trial 1. The behaviors were recorded using ANY-maze.

Food intake analysis

The mice had ad libitum access to mouse chow in the home cage before and after food intake trials. The 1-h food intake trial was performed for each mouse once a day at the same time (1:00 to 2:00 PM) and continued for 5 days. The average of 5-day intake was accounted to the food intake capacity of a mouse. Regular and sweet food were purchased from the Cooperative Medical Biological Engineering (Suzhou, China).

Normal distribution analysis

To determine whether the data follows a normal distribution, we used Shapiro-Wilk test and Quantile (Q)-quantile (Q) plot (Wang et al., 1998). Shapiro-Wilk test, which is advantageous for small to moderate sample sizes, evaluates whether a dataset significantly deviates from a normal distribution. Q-Q plot compares the quantiles of observed data against the expected quantiles of a theoretical normal distribution. If the data points align closely with the diagonal line, it indicates that the data is normally distributed. In brief, the data were sorted in an ascending order and calculated the empirical quantiles of data using the equation: sample_Q(i) = (i - 0.5)/n, where i was the position of a data point and n was the total number of data points. The theoretical quantiles of a standard normal distribution was calculated using the equation: theoretical_Q(i) = Φ^(-1)((i - 0.5)/n), where Φ^(-1) represented the inverse of the cumulative distribution function of normal distribution. Accordingly, the observed quantiles (sample_Q) on the x-axis against the theoretical quantiles (theoretical_Q) on the y-axis was plotted.

Quantification and statistical analysis

Experimenters who performed experiments and analyses were blinded to the genotypes until the data were integrated. Data were analyzed using Igor Pro 6.0, Graphpad Prism 8.0, SPSS 17.0, MatLab, and Rx64. Pearson's correlation was used to analyze the correlation. Statistical differences were determined using unpaired two-sided Student's t-test for two-group comparison or one-way ANOVA followed by LSD's post hoc test for multiple comparisons. The level for significance was set at $P < 0.05$. "*n*" represents the number of animals, cells, or batches depending on experimental design. Data in the text and figures are presented as the mean ± SEM.
